# Paradoxical regional contrasts in the resilience of marine zooplankton to climatic warming

**DOI:** 10.1038/s42003-026-10618-w

**Published:** 2026-07-17

**Authors:** Stefano Corona, Andrew Hirst, David Atkinson, Angus Atkinson

**Affiliations:** 1 School of Environmental Sciences, Jane Herdman Building, University of Liverpool, Liverpool, UK; 2 Department of Estuarine and Delta Systems, Royal Netherlands Institute for Sea Research, Korringaweg 7, Yerseke, The Netherlands; 3 Institute of Infection, Veterinary and Ecological Sciences, University of Liverpool, Liverpool, UK; 4 Plymouth Marine Laboratory, Prospect Place, The Hoe, Plymouth, UK; 5 Present Address: Stazione Zoologica Anton Dohrn, Villa Comunale, Naples, Italy

**Keywords:** Phenology, Population dynamics, Time series

## Abstract

To investigate the integrated response of plankton to climate warming, we measured simultaneous shifts in abundance, geographic range and phenology of eight copepod taxa across the North Sea and NE Atlantic over 6 decades. Here we show that the North Sea warmed about twice as rapidly as the NE Atlantic, yet its species showed greater resilience, maintaining more stable abundance and ranges. In contrast, the NE Atlantic experienced declining copepod populations and greater range shifts (up to 139 km northwards per decade). Regionally specific conditions (high food abundance and/or advection) in the North Sea, may have contributed to this resilience. Most taxa exhibited consistent seasonal shifts with warming (up to ~39 days earlier °C-1) in both areas. In the North Sea, species with greater range shifts also had more pronounced phenological shifts, suggesting a link between the two responses. Climate – smart marine management needs to incorporate this variable ecosystem resilience under warming, moving beyond temperature-centric models of the stability of ecosystems.

## Abstract (web summary)

Over 60 years, North Sea copepods proved more resilient to rapid warming than NE Atlantic populations, maintaining stable ranges despite faster warming, while both regions shifted seasonal timing earlier as temperatures increased.

## Introduction

In a warming world we urgently need improved projections of species distributions to better manage marine resources and appreciate how ecosystem functions will change. To predict these future patterns, a fundamental assumption of many species distribution models is that the relationship of a species distribution to specific environmental factors is broadly fixed and predictable^1–4^. Thus, a species would be assumed to maintain its distribution, for instance, within the same thermal range. Populations may respond to a warming climate by adjusting their geographic range polewards or by adjusting their seasonal timing (i.e. phenology) to earlier/later in the year. Shifts in range^4–6^ and phenology^7^ are considered to be two of the three “universal” responses to climate warming, alongside reductions in body size^8–10^. While there is often a shift poleward or earlier in the year under warming, these shifts are by no means as smooth or predictable as often suggested^11–13^. In models projecting future ectothermic species distributions, the fundamental assumption of a fixed thermal envelope often goes untested for the systems under study. Complementary responses, including partial resilience to change, need to be better understood for improved climate projections.

A first potential explanation for why distributional responses in space, or time, do not track climate warming is acclimatization and/or genetic adaptation to warmer conditions (niche plasticity)^14–16^. A second, recent, hypothesis^17^ predicts that when food resources increase, both the optimal temperature and breadth of thermal performance also increase, leading to greater thermal tolerance, and hence less pressure to shift range and seasonal timing. In this sense the thermal niche is modified by changing resource abundance (e.g. food), rather than by adapatation or acclimatization. If the species does not track its thermal optimum in space or time, and the two alternative mechanisms providing resilience are not possible, then the species may become increasingly disadvantaged and suffer a regional decline.

To better understand the multiple processes by which species conserve their thermal niche, or display niche plasticity, it is essential to measure distributional and abundance responses across both space and time over large spatial and temporal scales. Marine copepods are excellent candidates for this approach, since they are ectothermic (hence sensitive to thermal changes), multivoltine (multiple generations in a year) and planktonic, hence being rapidly dispersed. As such, their response can be detected temporally and geographically, often rapidly. Furthermore, most planktonic organisms are not commercially exploited, so any long-term change that is potentially relatable to global warming is less prone to being obscured by direct human activities such as fishing^18^. Besides being exemplars of climate change responses, copepods comprise up to 80% of the zooplankton biomass^19^, hence are crucial to the marine trophic web, representing an important energy link between the unicellular primary producers and higher trophic levels^20^.

Even among copepods, however, studies have tended to focus either on shifts in range^5^ or in phenology^21^, and we are only aware of one long-term study in which copepod phenology and range shifts are both assessed together^12^. Such an integrated approach is essential to understand how species keep pace with temperature change, both seasonally and spatially. While range shifts among marine plankton have been demonstrated^5,22,23^, as have phenology shifts^21,24^, most studies have not considered whether a species is fully tracking their thermal optimum (exhibiting thermal niche conservatism) or instead exhibiting some plasticity of their thermal niche.

In this study we analysed six decades of spatial and temporal shifts in abundance of multiple copepod species in one of the most intensively-sampled marine regions in the world. This region comprised two contrasting areas at similar latitudes, namely: (a) the slowly warming (~0.08 °C decade^−1^) North-East Atlantic, and (b) the very rapidly warming (~0.19 °C decade^−1^) North Sea. Comparing multiple copepod species, across two adjacent marine areas with such a different warming rate, provides an excellent natural experiment in climate change responses. By studying geographical range and phenological shifts of a suite of eight ecologically important taxa in these adjacent areas, across a 19° latitudinal range and over 58 years of warming, we aim to understand the mechanisms of thermal adjustment. Our goal is, first, to quantify the absolute change in latitudinal and seasonal distribution of copepod abundance over a multi-decadal period of warming. Second, we test whether the latitudinal distributions of each of the eight copepod taxa keep pace with the northwards shift in isotherms (as predicted by the thermal niche conservation principle) and how these shifts relate to the phenological shift at different latitudes and in adjacent seas. Our results are paradoxical, in that the greatest geographical range shifts were in the more slowly warming NE Atlantic (rather than in the North Sea), and we evaluate alternative hypotheses to explain instances where the thermal niche is not conserved.

## Results

### Warming and isotherm movement

**Fig. 1:**
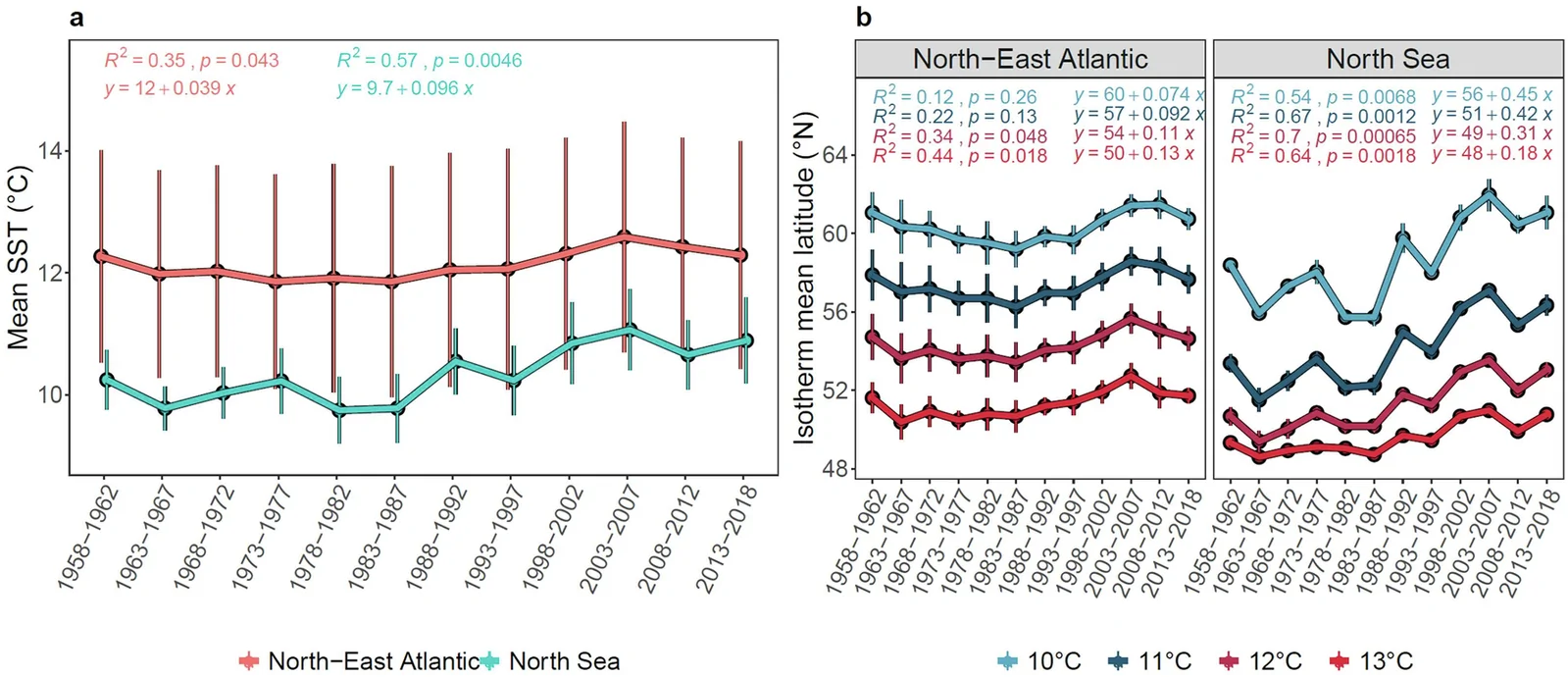
The North Sea has warmed much faster than the NE Atlantic. **a** Mean of the estimated SST values over the twelve five-year periods in the North-East Atlantic (in orange) and the North Sea (in light blue). **b** Mean latitudes of the 10, 11, 12 and 13 °C isotherms (colour indexed as indicated by the legend at the bottom) for each five-year period. The R², *p*-value and the equation from the linear regressions between the temperature on the y-axis (indexed by mean SST (°C) in (**a**) and mean isotherm latitude (°N) in (**b**)) and time on the x-axis (indexed by 5-year groups).

The mean SST of each consecutive five-year period has significantly increased over the years 1958 to 2018 in both the North-East Atlantic (NE Atlantic) and the North Sea (Fig. 1a). The rate of increase in SST in the NE Atlantic (~0.08 °C decade^−1^) has been slower, at around half the rate of the North Sea (0.19 °C decade^−1^). The 10, 11, 12 and 13°C isotherms all moved north in both marine areas (Fig. 1b). In the NE Atlantic, the warmer isotherms (12 and 13 °C) significantly increased in latitude over time, whereas the latitude of colder isotherms (10 and 11 °C) showed an initial reduction, representing cooling, between 1958 and 1987, and then a latitudinal increase, illustrating a warming period (Fig. 1b). In the North Sea, the colder isotherms shifted northward by a greater latitude than did the warmer isotherms: the coldest isotherm (10 °C) shifted northward very rapidly, on average by ~0.09°N latitude degree per year (Fig. 1b).

### Copepod geographic range shift

**Fig. 2:**
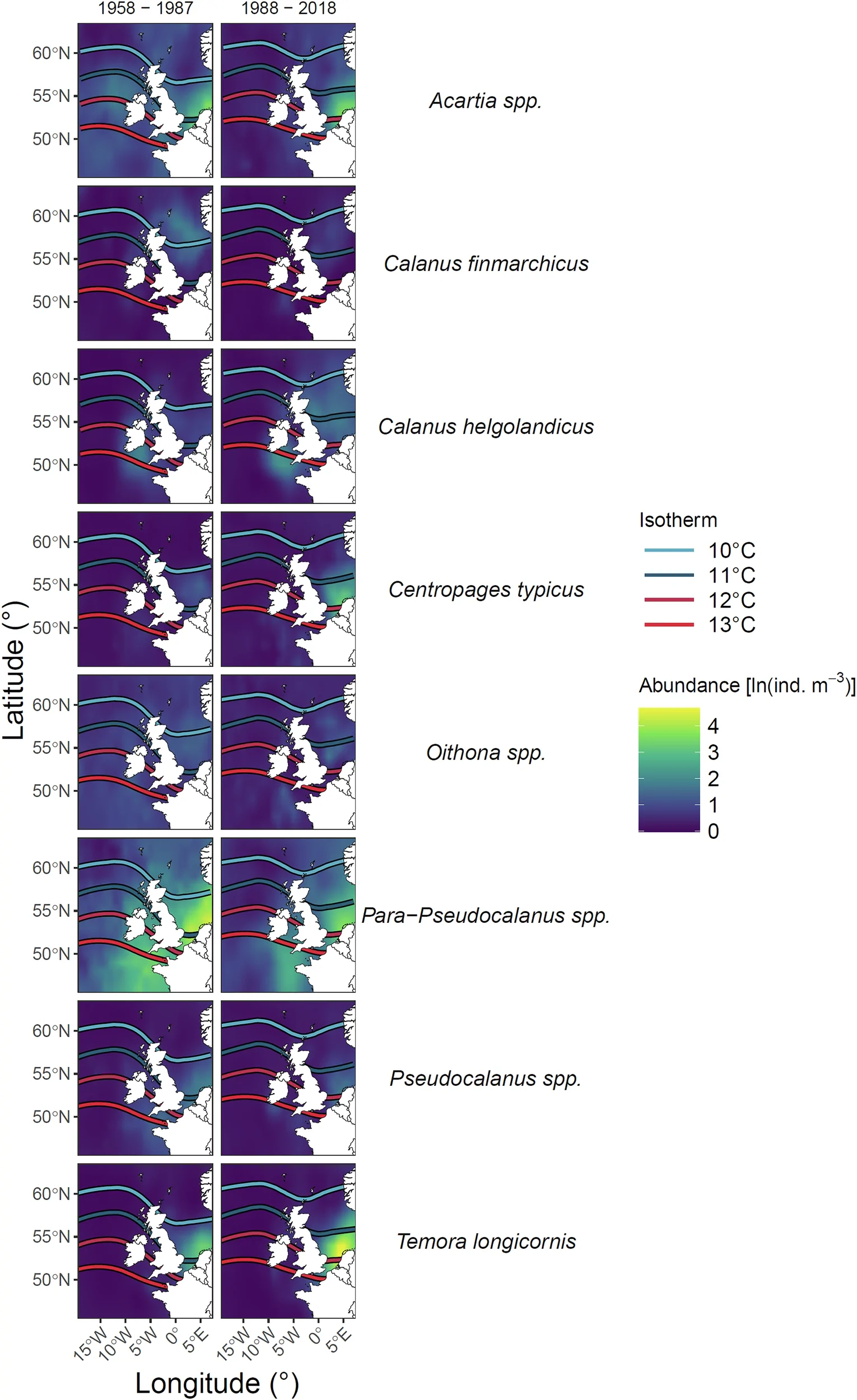
Geographic range shifts vary greatly between taxa. Geographic distribution of each copepod taxon in the whole area during the first (1958–1987) and last (1988–2018) 30 years of the time series. Bright areas (yellows and greens) indicate high copepod abundance (ln(x+1)) as indicated by the legend. Coloured lines indicate the four isotherms at different temperatures (10, 11, 12, 13 °C).

**Fig. 3:**
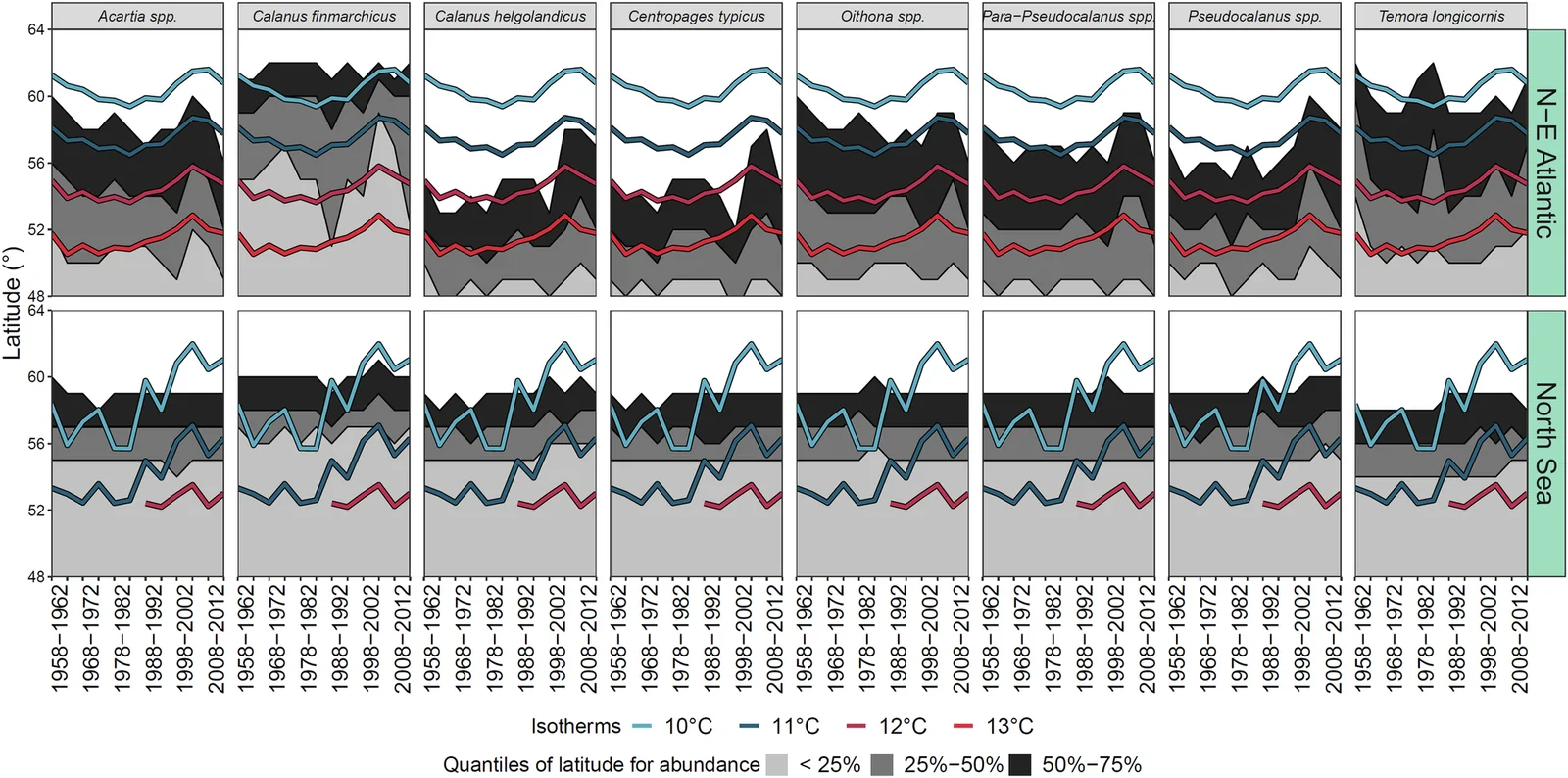
Copepod distributions in the NE Atlantic have shifted with the isotherms, but in the North Sea they have remained relatively fixed despite its faster warming. Long-term trends of the 25%, 50% and 75% quantiles of latitude for copepod abundance indicated by the three coloured bands. Superimposed lines indicate the long-term trend in the latitude of isotherms (colour indexed for four different temperatures as indicated by the legend).

Overall, the main pattern was a stronger and more consistent northward range shift response in the NE Atlantic compared to the more rapidly warming North Sea, where the populations did not track their thermal niche. Superimposed on this main pattern, mean copepod abundance over the whole area tended to decrease significantly over time. Only two taxa (*Centropages typicus* and *Calanus helgolandicus*) showed any significant increase in abundance, and only in the North Sea (see Table S.1). These trends can also be visualized in Fig. 2, which compares the abundance of each taxon between the first and the last half of the whole time-series, with Figs. S.1–S.8 showing more detailed maps. Abundance of the copepod taxa showed different latitudinal shift directions across the time-series in the NE Atlantic; some taxa showed an initial southward shift in the first half of the times-series (*Acartia* spp. and *Oithona* spp.), and some showed a strong northward shift during the last decades (*Calanus helgolandicus*, *Centropages typicus*, and *Pseudocalanus* spp.; Fig. 3), whereas in the North Sea, no clear latitudinal shifts emerge. This trend in the NE Atlantic broadly matches the shifting isotherms with cooling temperatures followed by warming, so we calculated the latitudinal shift against time for each copepod taxon (latitude corresponding to low, medium and high abundance: quantified as the 25th, 50th and 75th quantiles respectively) during the cooling period, from 1958–1962 to 1978–1982, and during the warming period, from 1983–1987 to 2003–2007 (see Table 1).

**Table 1:** Total movement (km) during the cooling period (from 1958–1962 to 1978–1982) and warming period (from 1983–1987 to 2003–2007) for each taxon in each of the two areas

	North-East Atlantic		North Sea	
Taxon	Total movement (km)—cooling period	Total movement (km)—warming period	Total movement (km)—cooling period	Total movement (km)—warming period
*Acartia* spp.	−173.90	184.70	−24.37	20.35
*Calanus finmarchicus*	136.90	222.00	3.70	151.98
*Calanus helgolandicus*	−199.80	94.91	−2.38	51.80
*Centropages typicus*	−17.96	−139.19	−49.95	57.35
*Oithona* spp.	−186.85	−90.65	7.40	−33.30
*Para-Pseudocalanus* spp.	−83.25	146.05	12.03	16.65
*Pseudocalanus* spp.	−142.45	347.80	−12.95	29.60
*Temora longicornis*	−442.15	−101.75	−12.95	55.48

Total movement is calculated as the mean of all three quantiles (25th, 50th, 75th) of latitude for copepod abundance vs. time (five-year periods) and then converted from latitudinal degree (°N) into km. Positive values indicate northward movement and negative values indicate southward movement.

**Fig. 4:**
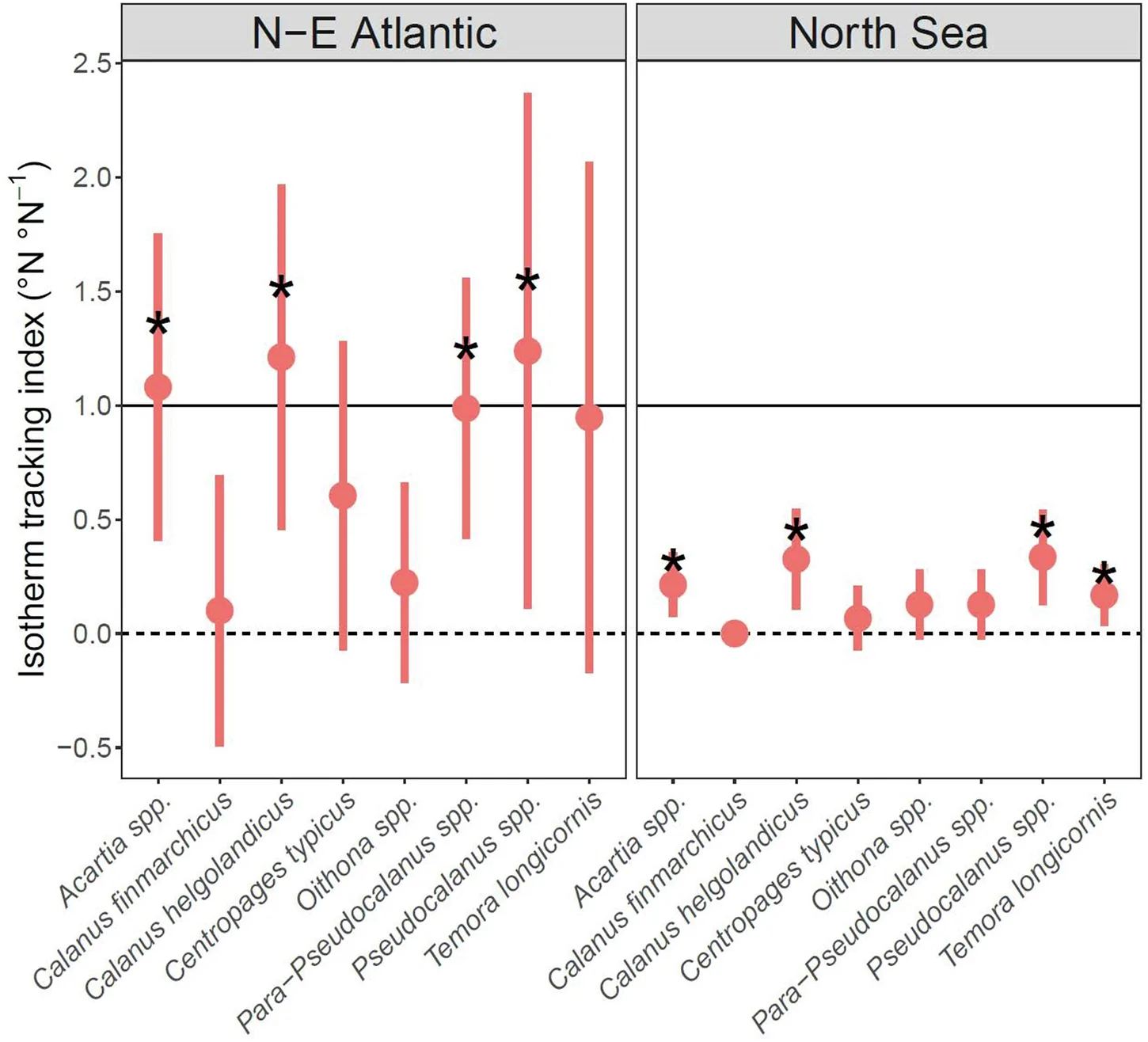
Copepods shift northward in both marine areas, but in the North Sea they track the isotherm shift less well. Summary of the taxon-specific linear regression slopes across twelve 5-year periods between 1958 and 2018 (*n* = 12) for latitude of copepod 50th percentile abundance vs. mean latitude of the 11 °C isotherm (°N °N^−1^) of each area. The 95% C.I. of each slope is represented as vertical error bars in each point. At y (isotherm tracking index) = 0 (dashed horizontal line) copepod abundance shows no latitudinal shift. Asterisks indicate significant increase in latitude of 50% abundance per shift in mean latitude of the 11 °C isotherm (*p ≤ 0.05). At y = 1 (solid horizontal line) the latitudinal shift of copepod abundance matches the latitudinal shift of the isotherm (indicating thermal niche tracking).

As the 11 °C isotherm moved northwards, so did the latitude at which a given percentile abundance of copepods was found, indicating a northern shift in distribution with warming (Fig. 4). However, in the NE Atlantic the copepod abundance more closely tracked the northward-moving isotherms (abundance shifted northward at the same pace as the isotherm) than it did in the North Sea. In half of the taxa (4 out of 8) in the NE Atlantic, the northward shifts were significantly different from 0, but were not significantly different from the isotherm shift, implying thermal niche conservation (copepod abundance moved northward at the same pace as the isotherm, Fig. 4). By contrast, the population abundance in the North Sea shifted northward with warming less intensely in all taxa, and never kept pace with the isotherm movement. Thus, the slopes of the 50th percentile of latitude for abundance against the 11 °C isotherm latitude, were always significantly different from 1, implying no thermal niche conservation (see Fig. 4). This failure to track isotherm shifts in the North Sea was also demonstrated by the decline in percentage of copepod abundance north of the 11 °C isotherm, with warming (Fig. S.9). By contrast, in the NE Atlantic the changes in percentage of copepod abundance north of each isotherm were never significantly negative, implying that copepods more closely tracked the movement of the isotherms with warming. The increase or decrease of abundance with warming throughout the whole latitudinal gradient of all taxa and areas is summarized in Fig. S.10, which shows that *Acartia* spp, *Oithona* spp, *Para-Pseudocalanus* spp., *Pseudocalanus* spp decreased in the south and increased in the north (although more intensely in the NE Atlantic), *C. helgolandicus* and *Centropages typicus* instead increased across the whole latitudinal gradient, but more intensely in the North Sea.

### Phenological shift

**Fig. 5:**
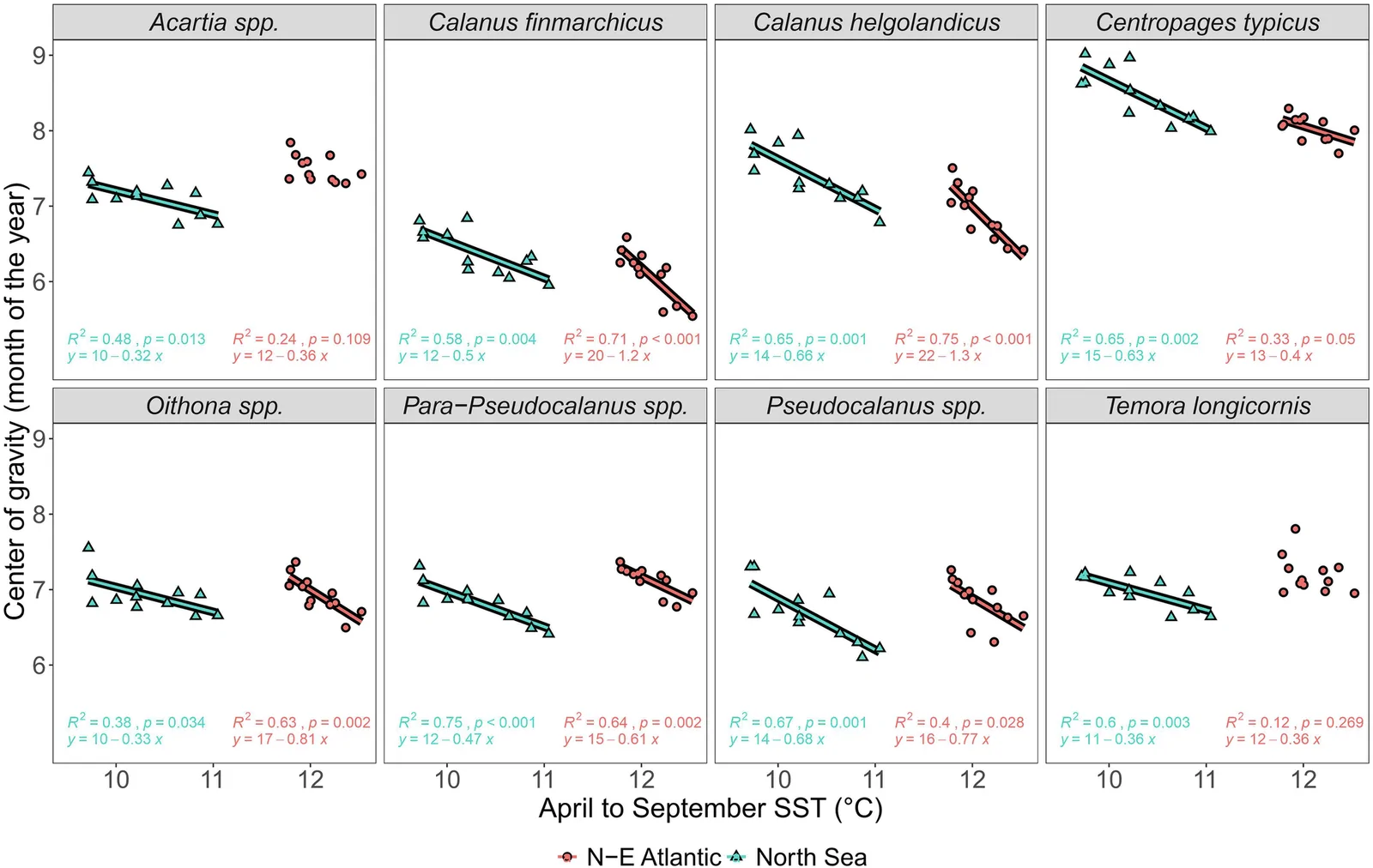
The phenology of copepods tends to shifts earlier in the year in both the North Sea and the North-East Atlantic. Linear regressions between the centre of gravity (COG) of abundance (expressed as Julian month) and the sea surface temperature (SST) between April and September (both included) for all taxa. Each point represents a 5-year period across 60 years. Negative slopes indicate a shift of abundance seasonal distribution toward the early part of the year with warming. At the bottom of each panel R², *p*-value and linear equations for each regression are reported. Fitted lines are only included when the relationship is significant (*p* < 0.05).

**Fig. 6:**
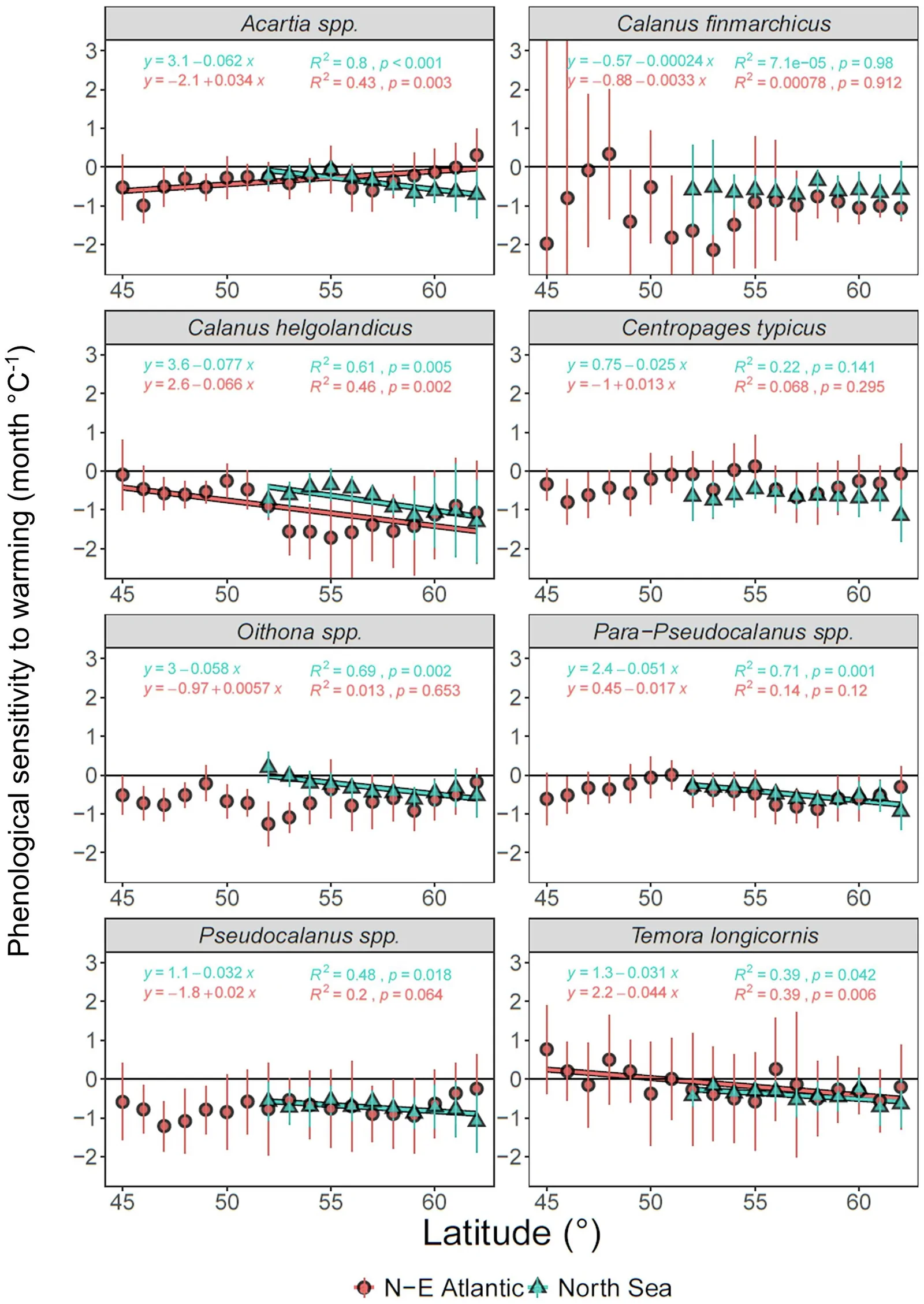
Phenological sensitivity to temperature increases with latitude in the North Sea. Distribution of phenological sensitivity to temperature values (slopes from the regressions between centre of gravity and temperature; with negative values indicating earlier phenology with warming, see Figure 7) on the y-axis at each latitudinal degree on the x-axis. Orange circles refer to the North East Atlantic, while blue triangles refer to the North Sea. Regressions across all data in the plots are fitted and reported when significant (for N-E Atlantic and the North Sea separately). Vertical lines for each point indicate the 95% C.I. of each slope, when these lines do not intersect the horizontal black line they indicate the slope is significant.

We detected a significant and consistent advancement of phenology, with shifts up to ~ 39 days earlier °C^−1^ (Fig. 5). Thus, the temporal centre of gravity (COG) of species abundance became significantly earlier in the year with increasing SST (averaged between the months of April to September inclusive). This advance in seasonal timing was significant for each taxon in both areas (the only exceptions being for *Acartia* spp. and *T. longicornis* in the NE Atlantic). In the North Sea, all taxa, except *C. finmarchicus* and *C. typicus*, showed a positive relationship between phenological sensitivity to temperature (slope of COG vs. SST) and latitude, which indicates greater phenological shifts toward the early part of the year at higher latitudes (Fig. 6). By contrast, in the NE Atlantic, only three taxa showed significant relationships. Thus, *Temora longicornis* and *Calanus helgolandicus* showed greater phenological shifts at northern latitudes, while *Acartia* spp. showed greater phenological shifts in the south (Fig. 6). A significant relationship between phenological and latitudinal range shifts across taxa was found only for the North Sea area (Fig. S.11). Thus, the thermal sensitivity of taxon-specific phenological shifts (slopes of COG vs. SST) negatively correlated with thermal sensitivity of range shifts (latitudinal shift in abundance vs. latitudinal shift of isotherm). In other words, taxa that shift more intensely in range (northward movement with warming) also shift more intensely in phenology (earlier in the year with warming).

## Discussion

Both the North-East Atlantic (NE Atlantic) and the North Sea have warmed between 1958 and 2018. Paradoxically, despite the North Sea warming much more rapidly than the NE Atlantic, the dominant copepods here withstood warming and retained relatively fixed geographic distributions, unlike in the NE Atlantic where they shifted their range substantially northwards, tending to track the shifting isotherms. Phenological shifts, by contrast, appear to be much more consistent both among taxa and areas, with seasonal abundance shifting earlier with warming. Ours is one of very few studies that examines the two of these complementary adjustment mechanisms simultaneously, which is arguably needed for a fuller appreciation of temperature responses. However, the relationship between these two responses is still complex: we detected a positive relationship between phenological- and geographic range shift in the North Sea (meaning that taxa that shifted more intensely in phenology also shifted more in their geographic range, Fig. S.11), however, this is based on 8 taxa, and further work is needed to explore the generality of this outcome for other taxa and areas.

We did not find the expected seasonal shift towards more “early with warming for early season species and later with warming for late season species” as predicted (but not found) by Mackas et al.^21^. Nevertheless, our observed phenological shifts indicate strong tendencies towards thermal niche conservatism^1^. Despite the fact that our results are based on monthly-resolved data, and from the near-surface layer, they are strikingly consistent: copepod seasonal distribution advances earlier in the year with warming. By contrast, phenological studies based on data from fixed stations have usually delivered statistically weaker and more mixed directions of phenological shift^17,25,26^. This lack of clear phenological response at fixed stations may arise from site-specific conditions at these typically inshore locations^17^. Continuous Plankton Recorder (CPR) data covers a much greater area of offshore regions and for much longer time series, possibly leading to better visibility of macroscale patterns such as phenology and range shifts.

A key finding of our study was the great contrast in temperature responses for the same taxa between adjacent sea areas at equivalent latitudes. Contrary to our expectation that the rapidly-warming North Sea would be a hotspot for geographical range shifts or phenological adjustment^1,27^, range shifts in the NE Atlantic were much stronger in both magnitude and consistency among taxa. Likewise, another recent study by Holland et al.^28^, comparing these same two areas but focused on long-term plankton abundance trends instead of geographical range shifts, found much more stable abundances for major phyto- and zooplankton functional groups in the North Sea compared to the general declines seen in the NE Atlantic.

What is the cause of these counter-intuitive responses to rapid warming? One possible explanation involves the differences in circulation and advective supply between the NE Atlantic and the North Sea. The northward shift of copepod abundance in the North Sea may be countered by the inflows of relatively cool, species-rich waters via the Norwegian Coastal Current, cold outflows from the Norwegian Trench, and inflows from the Scottish Coastal Current^29^. Such advective southward inputs may counteract surface warming signals and stabilize population structure, analogous to the well-documented role of subarctic inflows from the Labrador Current and Scotian Shelf Current in sustaining *Calanus finmarchicus* populations in the Gulf of Maine^30,31^. In contrast, the NE Atlantic is more directly exposed to the prevailing northward-flowing surface currents that tend to reinforce poleward thermal displacement. These regionally specific circulation regimes may therefore modulate the extent to which populations track isotherms, potentially buffering North Sea copepods from rapid redistribution.

Another, not mutually exclusive, explanation for contrasting shifts between the NE Atlantic and North Sea may align with the predictions of^32^: when resources increase, the optimal temperature and breadth of thermal performance also increase, leading to greater thermal resilience. Consequently, increased food resources in the North Sea may reduce the need for extensive adjustments in geographical range and phenology. The NE Atlantic has a more homogeneous hydrography and distribution of chlorophyll, which can sometimes even increase with increasing latitude^33^. The North Sea, by contrast, has a greater chlorophyll concentration in its southern part^34^, due to the different hydrographic zones^35^. This is also in agreement with^36^ and^37^, who found a greater copepod production in the southern North Sea than in the northern part. Satellite data show that the highest chlorophyll *a* concentrations reside between 52° and 54° N in the North Sea (Fig. S.12). At these latitudes, the decreases in copepod abundance with warming were less intense for most taxa in the North Sea than in the NE Atlantic (Fig. S.10), implying that having a greater availability of food in the North Sea, may compensate for the increased food demand caused by warming^38^. We speculate that the greater chlorophyll *a* concentration in the southern North Sea (associated with greater phytoplankton biomass) leads to smaller phenological shifts, whereas the low food presence in the northern part corresponds with stronger phenological shifts, supporting the resource compensation hypothesis. Comparable mechanisms have already been observed in the Gulf of Maine^31,39^ and reinforce the broader relevance of resource-driven phenological plasticity across temperate ecosystems. However, the connection between resources and thermal sensitivity still remains to be investigated. For example, in the North Sea, phenological sensitivity to temperature becomes gradually more intense with increasing latitude (Fig. 6), whereas chlorophyll *a* shows an abrupt decrease from south to north (Fig. S.12). Furthermore, chlorophyll *a* is only a crude proxy for food availability, and given the debate over whether copepods are resilient or not to warming^5,13^ more work is clearly needed to explore such interactions.

In conclusion, we show highly contrasting degrees of resilience to climate warming in the eight most abundant copepod taxa over six decades. This adds a major caveat to those models that predict responses to global warming based on fixed responses to the thermal environment, and adds to a growing body of literature suggesting variable degrees of resilience of taxa to warming^13–15,40^. Models of change hotspots for biota, based only on topography and the pace of temperature change^27^ may be inadequate to capture the dynamics of populations in productive areas. It is exactly these productive shelf areas, such as the North Sea, that tend to be most heavily exploited by humans, and which in our study are hotspots of apparent greater thermal resilience rather than hotspots of species replacement. “Climate smart” marine planning aims to provide protection that accounts for climate change, for instance protecting important species or habitats in climate change refugia or “bright spots” where conditions may improve^41,42^. Our study emphasises the need for caution in such approaches^43^, especially in the crucial detail of how resilience and sensitivity to warming are defined. Our results underscore that such resilience may emerge from regionally specific interactions among circulation, advective supply and resource availability, suggesting that temperature-centric models may underrepresent the complexity of future biogeographic change.

## Methods

### Data collection

Copepod abundance data were obtained by the Continuous Plankton Recorder (CPR) survey, based currently at the Marine Biological Association of the UK (MBA) and published by The Archive for Marine Species and Habitats Data (https://www.dassh.ac.uk/)^44^. The CPR devices have been in use since 1931, making it the longest-running plankton sampling survey in the world^45^. The CPR samples plankton while being towed behind ships of opportunity at a depth of ~ 6 to 7 m. Plankton are filtered by a scrolling mesh of 270 µm and sandwiched by a second mesh, before being fixed in 4% formaldehyde. Data are not collected less than 10 km from the coastline to avoid interference from local land conditions. For our study, we used the abundance values (measured as individuals m^−3^) of eight copepod taxa (these values refer to the late copepodite stages, see ref. ^46^ for more information). Four of these were identified to the species level: *Calanus finmarchicus, Calanus helgolandicus, Centropages typicus, Temora longicornis*; three taxa were identified to genus level: *Acartia* spp., *Oithona* spp., *Pseudocalanus* spp., comprising a single dominant species (respectively *Acartia clausi*, *Oithona similis* and *Pseudocalanus elongatus*); and another was a mix of *Paracalanus* spp. and *Pseudocalanus* spp. (generally referred to as “*Para-Pseudocalanus* spp.”). The analysis was applied to a geographical area between 20° W and 8° E longitude and between 45° N and 64° N latitude (Fig. 1), over a 60-year time span from 1958 to 2018. Sea surface temperature (SST) values were obtained by the Hadley Centre of the UK Meteorological Office (http://www.metoffice.gov.uk/hadobs/hadisst/). These monthly temperature values were taken during daylight, for a 1° latitude-longitude grid and are the product of a mix of observation and interpolation. For a more detailed description of the SST dataset and its production process see ref. ^47^. Lastly, we used the chlorophyll *a* concentration time-series in order to test the possible effect of food on the copepod phenological sensitivity change across latitudes. Chlorophyll data were extracted for the same marine areas as the CPR survey. The regional monthly chlorophyll climatology maps are calculated over the 1997–2018 period by averaging monthly chlorophyll values on a pixel-by-pixel basis. Both the time series and the climatology maps were calculated using the global merged multi-sensor product OCEANCOLOUR_GLO_CHL_L3_REP_OBSERVATIONS_009_065 distributed by the Copernicus Marine Environment Monitoring Service (https://marine.copernicus.eu/). This product was derived from the OC-CCI v4.0 data set produced by the ESA Ocean Colour Climate Change Initiative (ESA OC-CCI^48^).

### Data extraction

Data analysis followed the general approach of^12^. Copepod abundance and SST data were averaged by year and by each 1° latitude ×1° longitude section from the area between 45°N and 64°N latitude, and between 20°W and 8°E longitude. The number of samples (equal for each taxon) was very similar for each month of the year (mean count: ~18892; SD: 1203) and each hour of the day (mean count: ~9491; SD: 292), hence showing no major biases toward a certain part of the year, or time of the day (see Fig. S.13). We interpolated copepod abundance by kriging the five-year averages of ln(Abundance + 1) values for each taxon (following the method in 15). To avoid potential biases, we only considered grid cells that were sampled in more than 25% of all sampling months over the whole time series (for geographical data coverage, see Fig. S.14). Kriging was performed using packages “sp”^49^, “gstat”^50^ and “automap”^51^ in R. The former package was used to determine the best fitting variogram by using the autovariogram function for each species and each five-year period. The sea surface temperature (SST) values were used to calculate the latitude of the isotherms at 10, 11, 12 and 13 °C for each 1° longitude in each five-year period (these isotherms were chosen to straddle the centre of the geographic area and taxon ranges sampled, see Fig. 2). To do this, we performed polynomial regressions between the averaged SST against latitude at each longitude degree. We subdivided the area into two parts: the North-East Atlantic (NE Atlantic), which comprises the CPR areas B4, B5, C3, C4, C5, D4, D5, E4, E5, and the North Sea (North Sea), which comprises the CPR areas B1, B2, C1, C2, D1, D2 (see Fig. S.15). The same kriging method was applied to the chlorophyll *a* dataset as that used for copepod abundance. We log_10_-transformed the concentration values (calculated as mg m^−3^) and averaged the overall (across all years from 1998 to 2018) abundance between and including April and September, for each latitudinal degree, in order to test whether the different amount of food across latitudes would match the latitudinal difference in phenological sensitivity.

### Range shift analysis

First, we assessed the latitudinal change in SST over time, by regressing the average latitude of each isotherm against year (averaging across all longitudes). Then, in order to estimate the copepod geographic range shift, we calculated the latitude corresponding to low, medium and high abundance for each copepod taxon (i.e. 25^th^, 50^th^ and 75^th^ quantiles of copepod abundance) in the two distinct marine areas (NE Atlantic and North Sea), across all longitudes, using the function wtd.quantile in R (package: Hmisc^52^). We regressed these latitudes for each measure of copepod abundance against a measure of SST (the latitude of the 11 °C isotherm, chosen because it covers both the NE Atlantic and the North Sea). We then calculated the average percentage copepod abundance north of each isotherm (relative to the abundance south of the same isotherm) for each five-year period and for each taxon, following^12^. These percentage values were linearly regressed against the mean latitude of each corresponding isotherm, to determine whether copepods changed their latitudinal distribution at the same rate as the isotherms shift with warming. To avoid potential biases related to the different latitudinal ranges between the two marine areas, we repeated these analyses by considering the same latitudinal range (between 52° and 63° N) for both areas.

### Phenology analysis

To calculate the phenological shift, we first applied kriging interpolation for the abundance of each taxon for each five-year period for each month. From these values we then calculated the centre of gravity (measured as month of the year) of abundance for each year across the whole geographical range. The centre of gravity method allows us to determine in which part of the year the copepod abundance is centred, i.e. earlier or later in the year (see^21,24,53^). The centre of gravity *C* (in Julian months) was calculated using the following formula:

$$C=\sum ({N}_{M}* M)/\sum {N}_{M}$$

Where *N*_*M*_ is the abundance of each taxon at each month *M*. We regressed the centre of gravity indices against yearly SST (average between April and September) to measure the long-term phenological shift (we than repeated the same analysis for each latitudinal degree). We then regressed taxon-specific latitudinal shifts against phenological shifts, in order to detect possible correlations between the two responses to climate change (latitudinal and phenological shifts).

### Reporting summary

Further information on research design is available in the Nature Portfolio Reporting Summary linked to this article.

## Supplementary information

Supplementary information

Description of Additional Supplementary Files

Supplementary Data 1

Reporting Summary

Transparent Peer Review file

## Data Availability

The data that support the findings of this study can be found in the Supplementary Data file and are also publicly available upon request at the following websites: 1. The plankton population data are available from The Uk Archive for Marine Species and Habitats Data (DASSH): https://dassh.ac.uk/. 2. The temperature data are available from the Hadley Centre Sea Ice and Sea Surface Temperature data set (HadISST): http://www.metoffice.gov.uk/hadobs/hadisst/. 3. The Chlorophyll *a* data are available from the Copernicus Marine Environment Monitoring Service (CMEMS): https://marine.copernicus.eu/it.
