# Supplementary material for: Paradoxical regional contrasts in the resilience of marine zooplankton to climatic warming
Source: Commun Biol. 2026 Jul 17;9:1490. doi: 10.1038/s42003-026-10618-w (PMC13748652; doi:10.1038/s42003-026-10618-w)
Supplement: Supplementary file 1 [file 42003_2026_10618_MOESM1_ESM.pdf]

SUPPLEMENTARY INFORMATION

Table S.1: Slopes (β), p and R^2 values of each linear regression of mean abundance [i.e. ln(x+1)] vs. time (5-year periods, n=12).

Taxon	Marine area	β	*p*	R^2
Acartia spp.	N-E Atlantic	**-0.25**	**0.00**	**0.69**
Calanus finmarchicus	N-E Atlantic	-0.03	0.10	0.24
Calanus helgolandicus	N-E Atlantic	0.05	0.13	0.22
Centropages typicus	N-E Atlantic	0.06	0.19	0.17
Oithona spp.	N-E Atlantic	**-0.21**	**0.00**	**0.69**
Para-Pseudocalanus spp.	N-E Atlantic	**-0.14**	**0.00**	**0.61**
Pseudocalanus spp.	N-E Atlantic	**-0.10**	**0.01**	**0.52**
Temora longicornis	N-E Atlantic	0.00	0.96	0.00
Acartia spp.	North Sea	-0.09	0.10	0.24
Calanus finmarchicus	North Sea	**-0.20**	**0.00**	**0.74**
Calanus helgolandicus	North Sea	**0.27**	**0.00**	**0.72**
Centropages typicus	North Sea	**0.21**	**0.01**	**0.51**
Oithona spp.	North Sea	**-0.19**	**0.01**	**0.54**
Para-Pseudocalanus spp.	North Sea	**-0.15**	**0.01**	**0.53**
Pseudocalanus spp.	North Sea	**-0.11**	**0.02**	**0.42**
Temora longicornis	North Sea	0.02	0.21	0.15

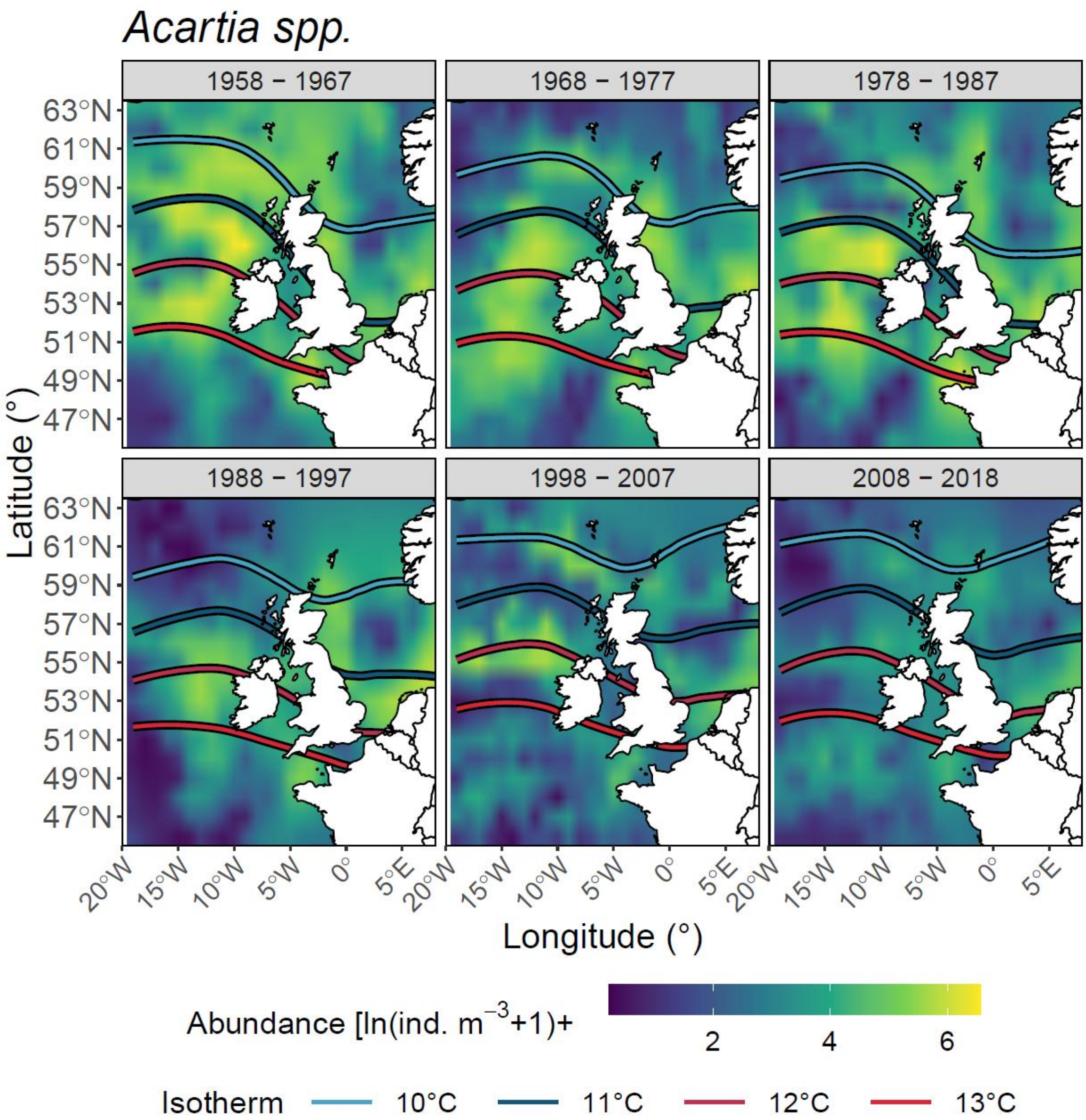

Figure S.1: Mean copepod abundance [i.e. ln(ind. m^{-3} + 1)] of combined pairs of five-year periods. Lines indicate the four different isotherms, colour indexed according to the legend at the bottom.

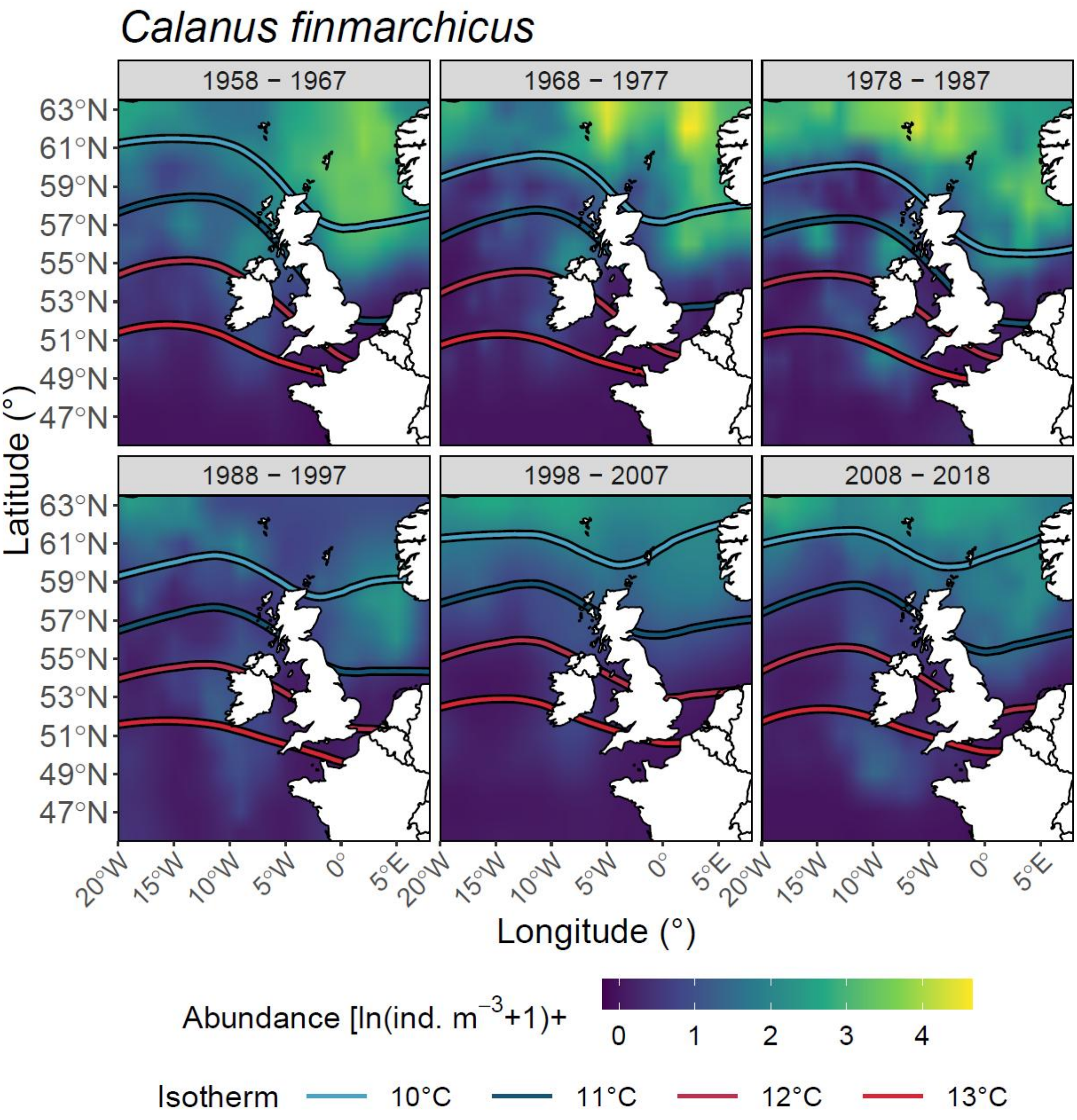

Figure S.2*:* Mean copepod abundance [i.e. ln(ind. m^{-3} + 1)] of combined pairs of five-year periods. Lines indicate the four different isotherms, colour indexed according to the legend at the bottom.

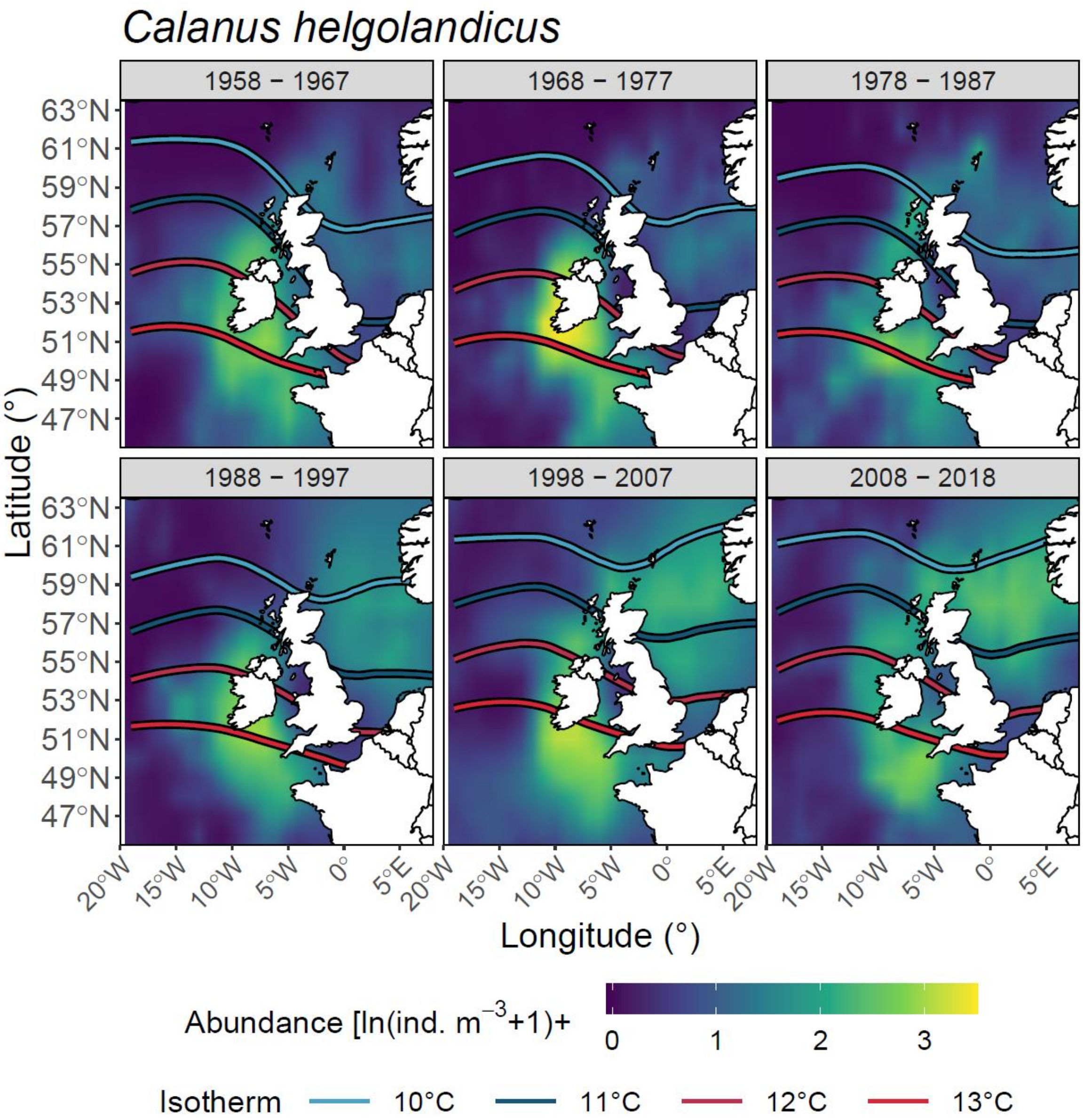

Figure S.3: Mean copepod abundance [i.e. ln(ind. m^{-3} + 1)] of combined pairs of five-year periods. Lines indicate the four different isotherms, colour indexed according to the legend at the bottom.

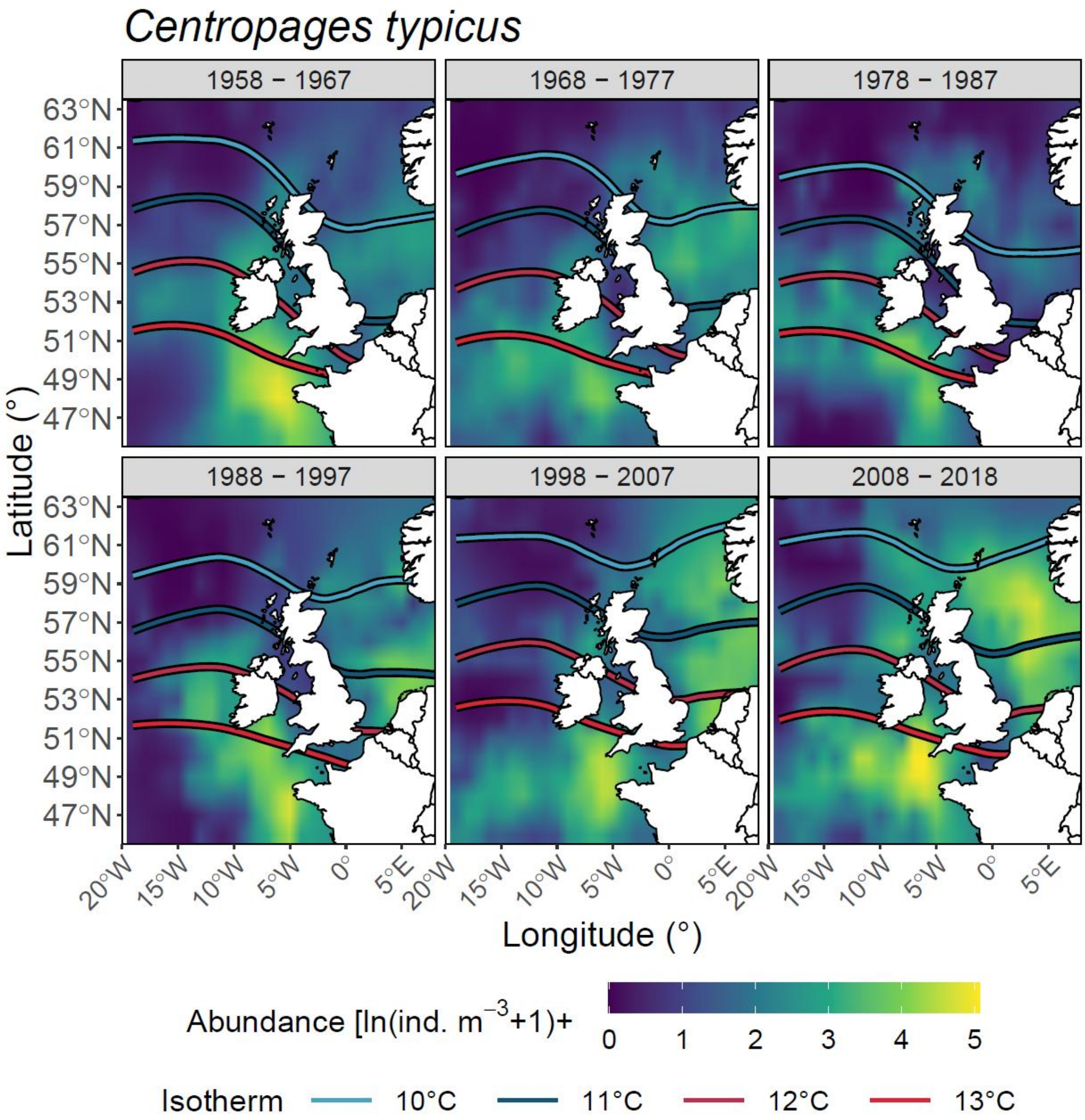

Figure S.4*:* Mean copepod abundance [i.e. ln(ind. m^{-3} + 1)] of combined pairs of five-year periods. Lines indicate the four different isotherms, colour indexed according to the legend at the bottom.

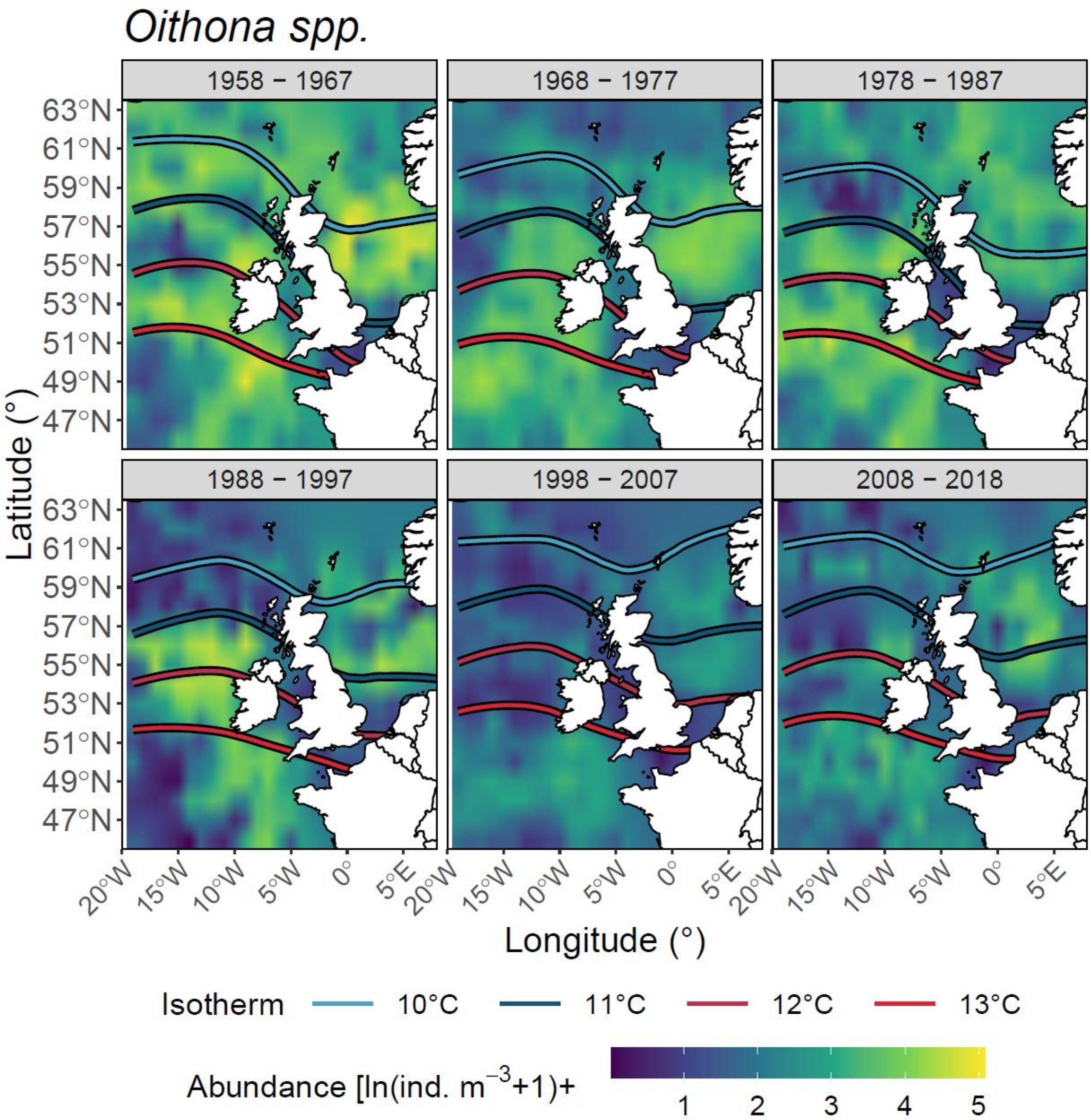

Figure S.5: Mean copepod abundance [i.e. ln(ind. m^{-3} + 1)] of combined pairs of five-year periods. Lines indicate the four different isotherms, colour indexed according to the legend at the bottom.

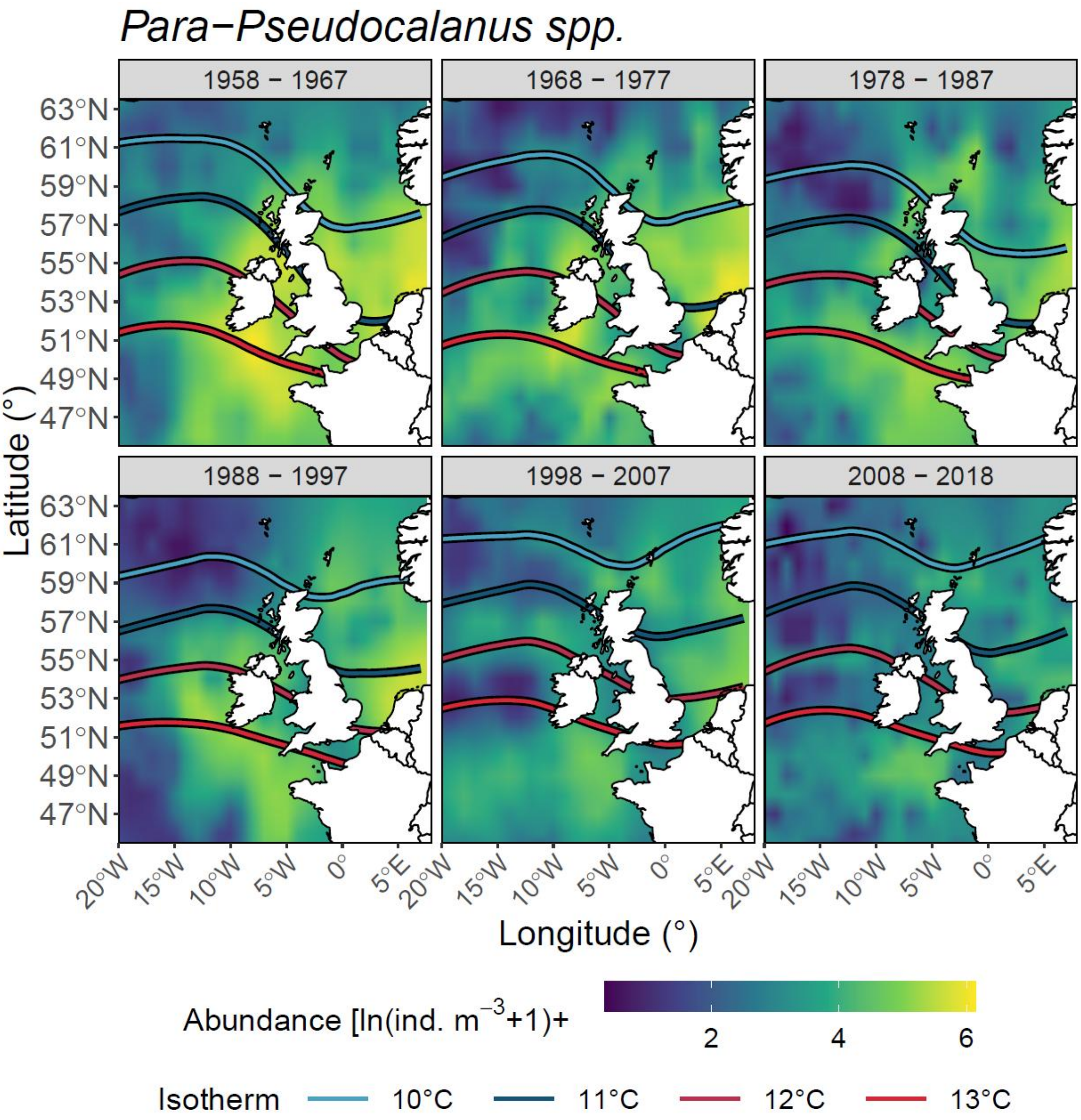

Figure S.6: Mean copepod abundance [i.e. ln(ind. m^{-3} + 1)] of combined pairs of five-year periods. Lines indicate the four different isotherms, colour indexed according to the legend at the bottom.

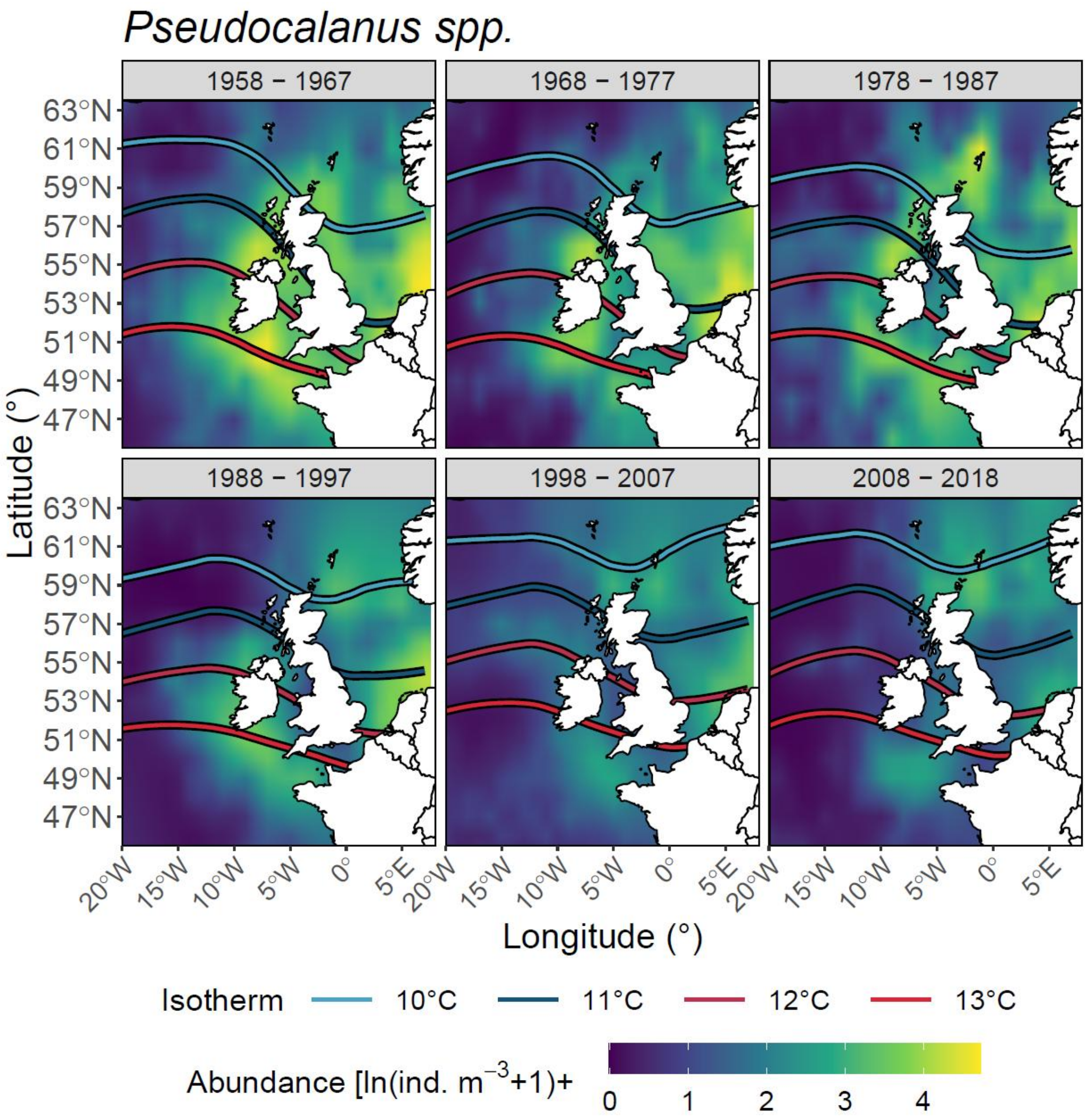

Figure S.7: Mean copepod abundance [i.e. ln(ind. m^{-3} + 1)] of combined pairs of five-year periods. Lines indicate the four different isotherms, colour indexed according to the legend at the bottom.

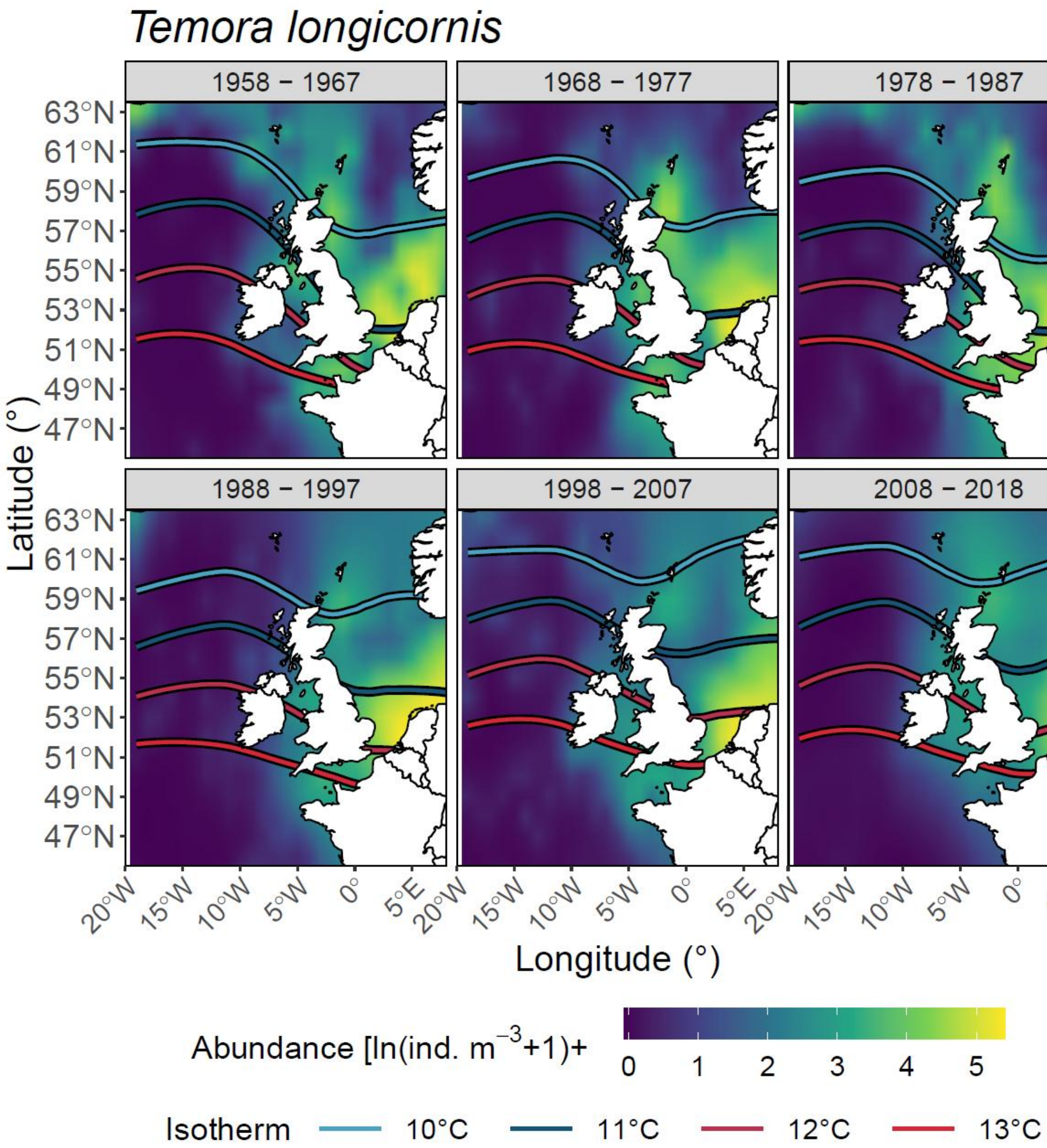

Figure S.8: Mean copepod abundance [i.e. ln(ind. m^{-3} + 1)] of combined pairs of five-year periods. Lines indicate the four different isotherms, colour indexed according to the legend at the bottom.

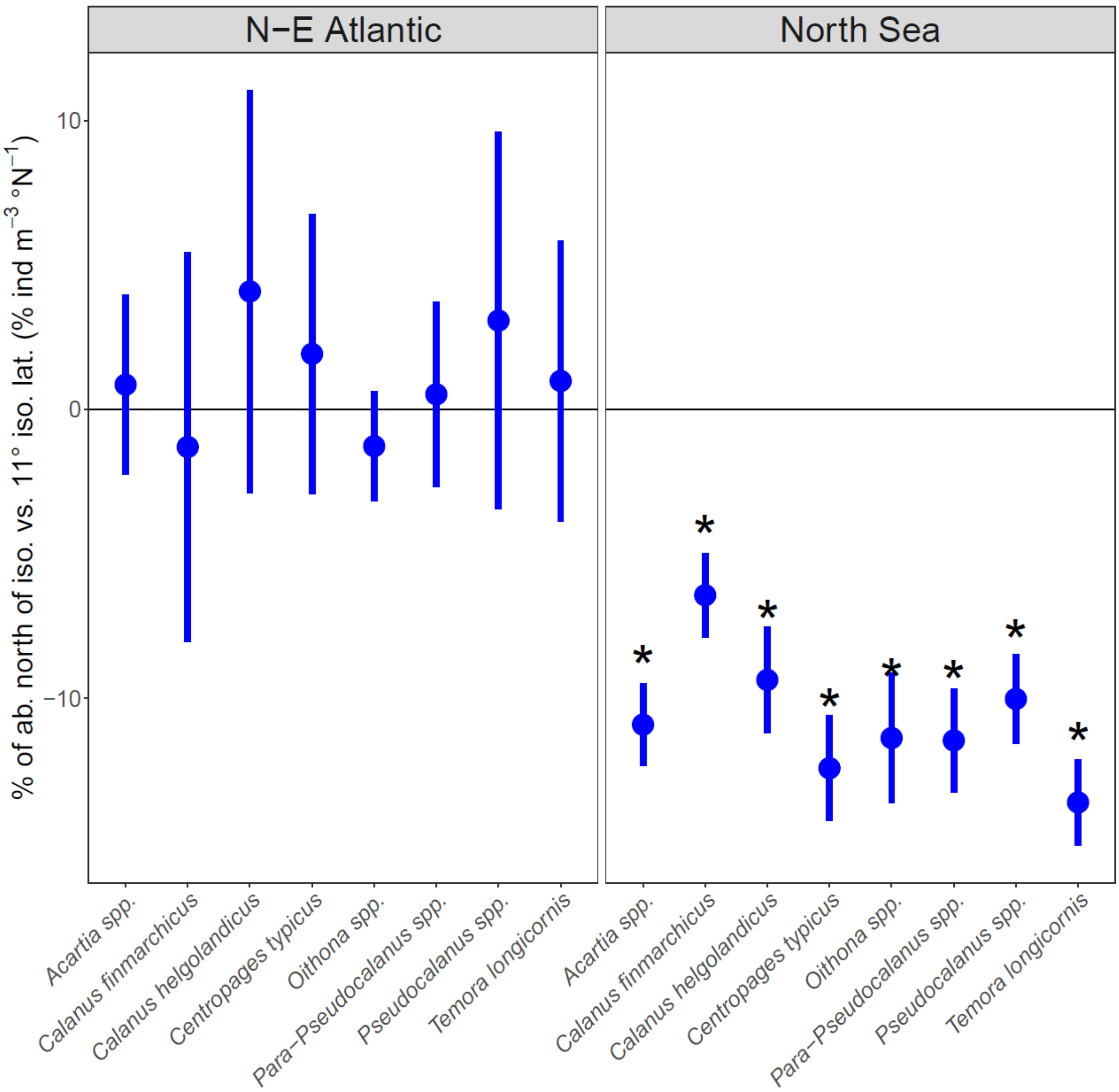

Figure S.9: Change of copepod abundance percentage north of the isotherm with warming. Taxon-specific linear regression slopes of percentage abundance north of the 11°C isotherm vs. mean 11°C isotherm latitude (% ind. m^{-3} $°N^{-1}$) across twelve 5-year periods (n = 12). Vertical error bars indicate the 95% confidence interval, an asterisk next to the symbol denotes a significant slope (* = p ≤ 0.05).

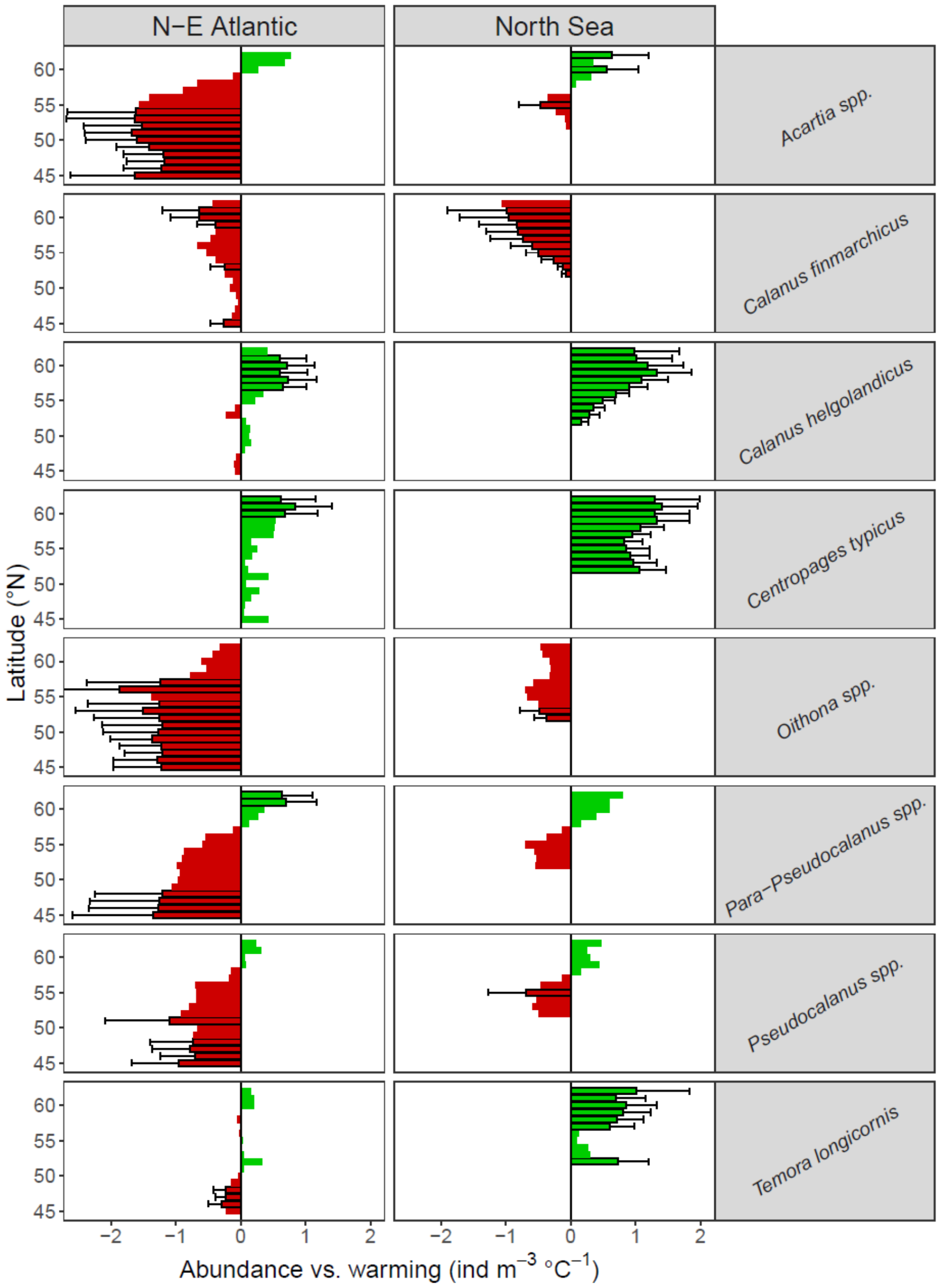

Figure S.10: Latitudinal variation in sensitivity to warming of copepod abundance between 5-year means across the period 1958 to 2018 : Bars indicate the slope values (x-axis) from the linear regressions of mean copepod abundance (log values) vs. 11°C isotherm latitude for each latitudinal degree (y-axis) of each 5-year period. Green bars on the right of the vertical black line indicate an increase, while red bars on the left indicate a decrease of copepod abundance with warming within that latitude. Horizontal error bars indicate the 95% confidence interval and are reported only for significant slopes. Grey areas represent missing values due to land presence at the given latitude.

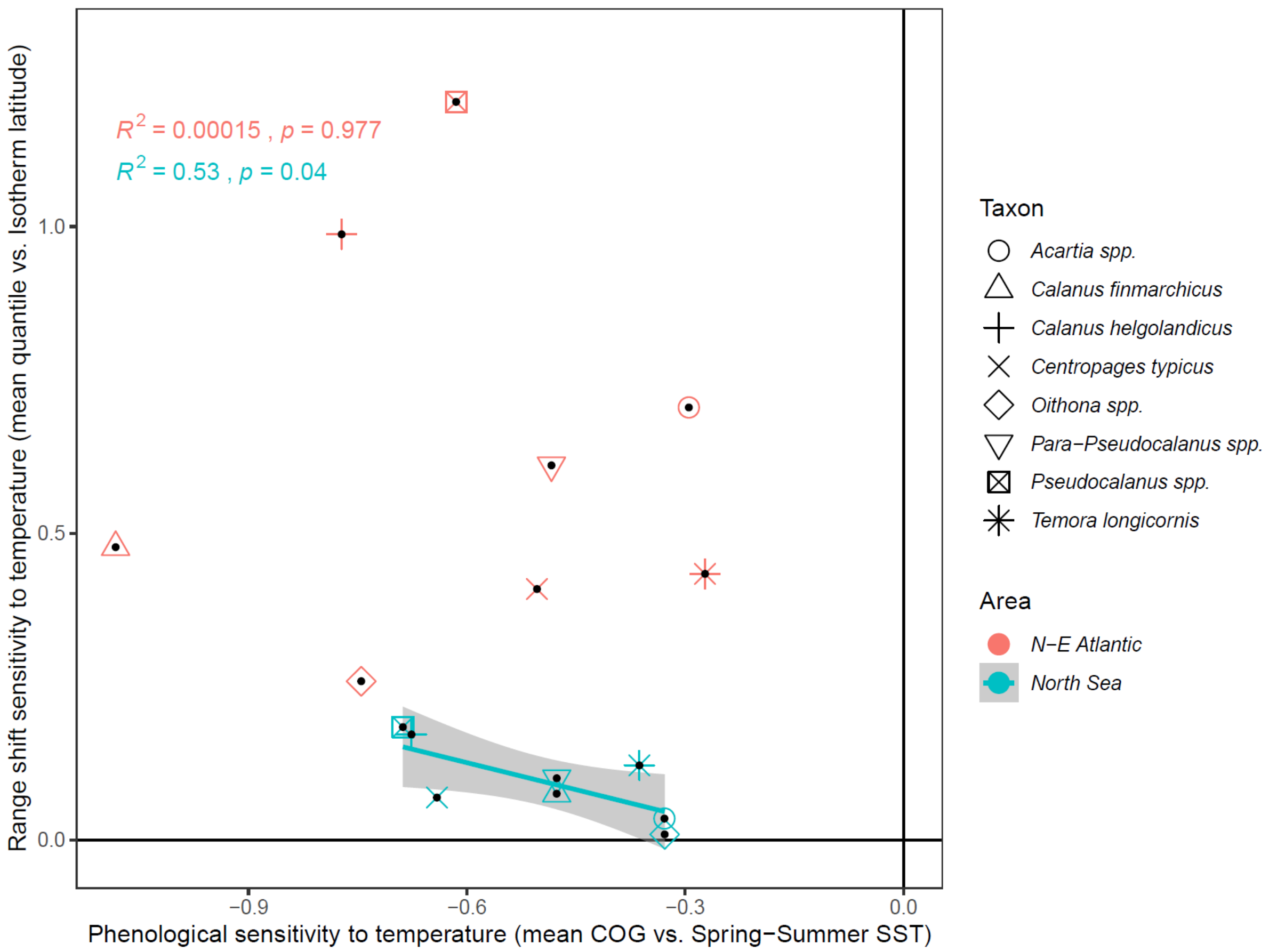

Figure S.11: **Relationship between range shift sensitivity to warming and phenological sensitivity to warming**. The y-axis indicates the thermal sensitivity of range shift: movement of quantiles (average across the 25th, the 50th, and the 75th) of latitude for copepod abundance with isotherm latitude change (N° N°$^{-1}$), meaning that values above the 0 indicate a northward movement with warming. The x-axis indicates the thermal sensitivity of phenology shift: change of COG month with Spring-Summer sea surface temperature (Julian month °C^{-1}), meaning that values < 0 indicate progressively earlier phenology with warming. Regression fit line is fitted only for the significant relationship in blue. On top-left are reported the R^2 and p values for both regressions (for both marine areas). The regression in blue means that there is a correlation between range shift thermal sensitivity and phenological thermal sensitivity, across species, in the North Sea.

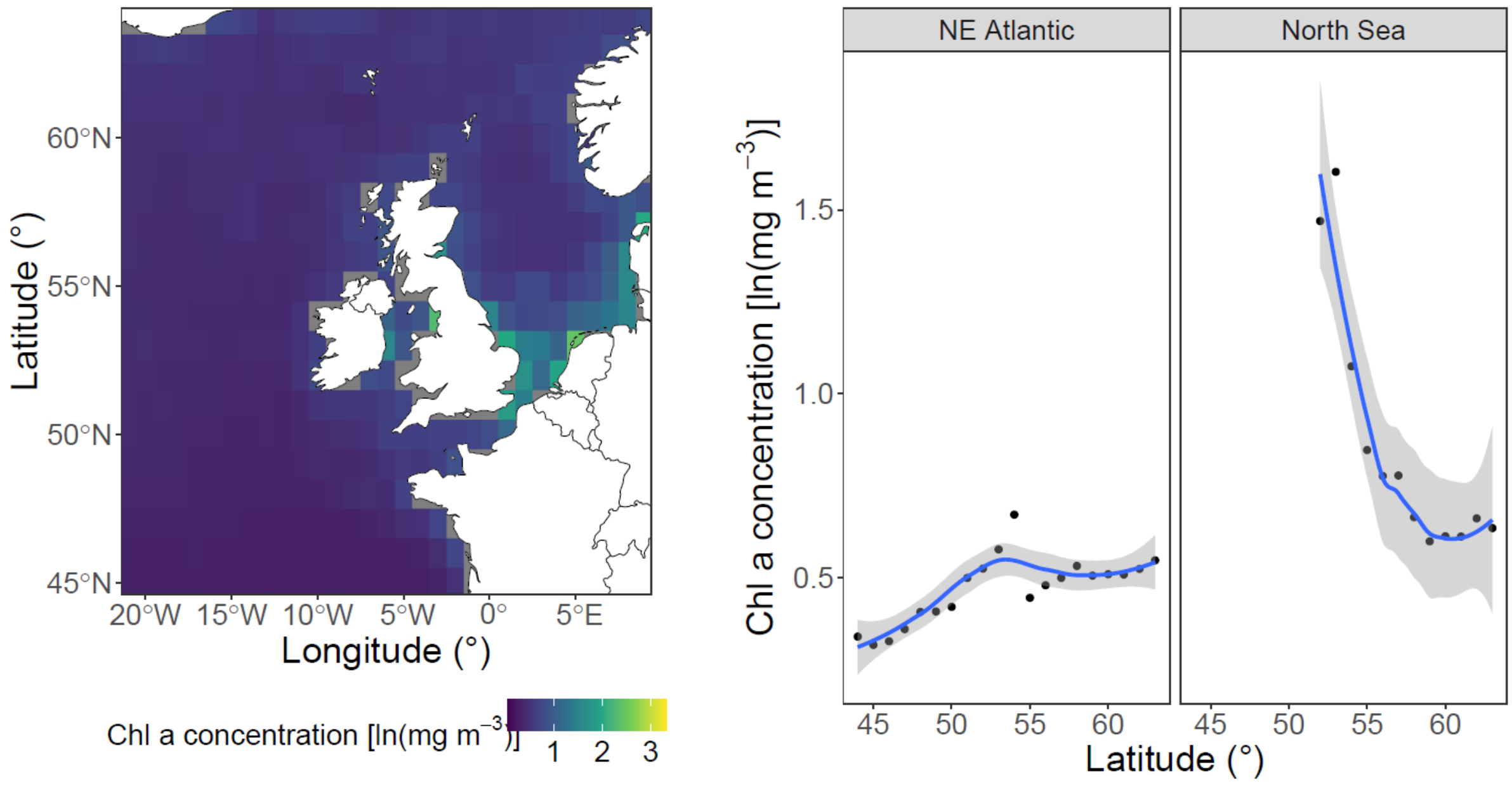

Figure S.12: Chlorophyll *a* concentration. **a)** Satellite-based (OC-CCIv6 data) for surface Chl *a* concentration across the study area; **b)** points indicate the Chl *a* concentration averaged for each 1° of latitude and for each marine area, blue line is the smoothed average (span level = 0.75). In both a) and b) Chl *a* concentration is measured as the mean value between April and September inclusive.

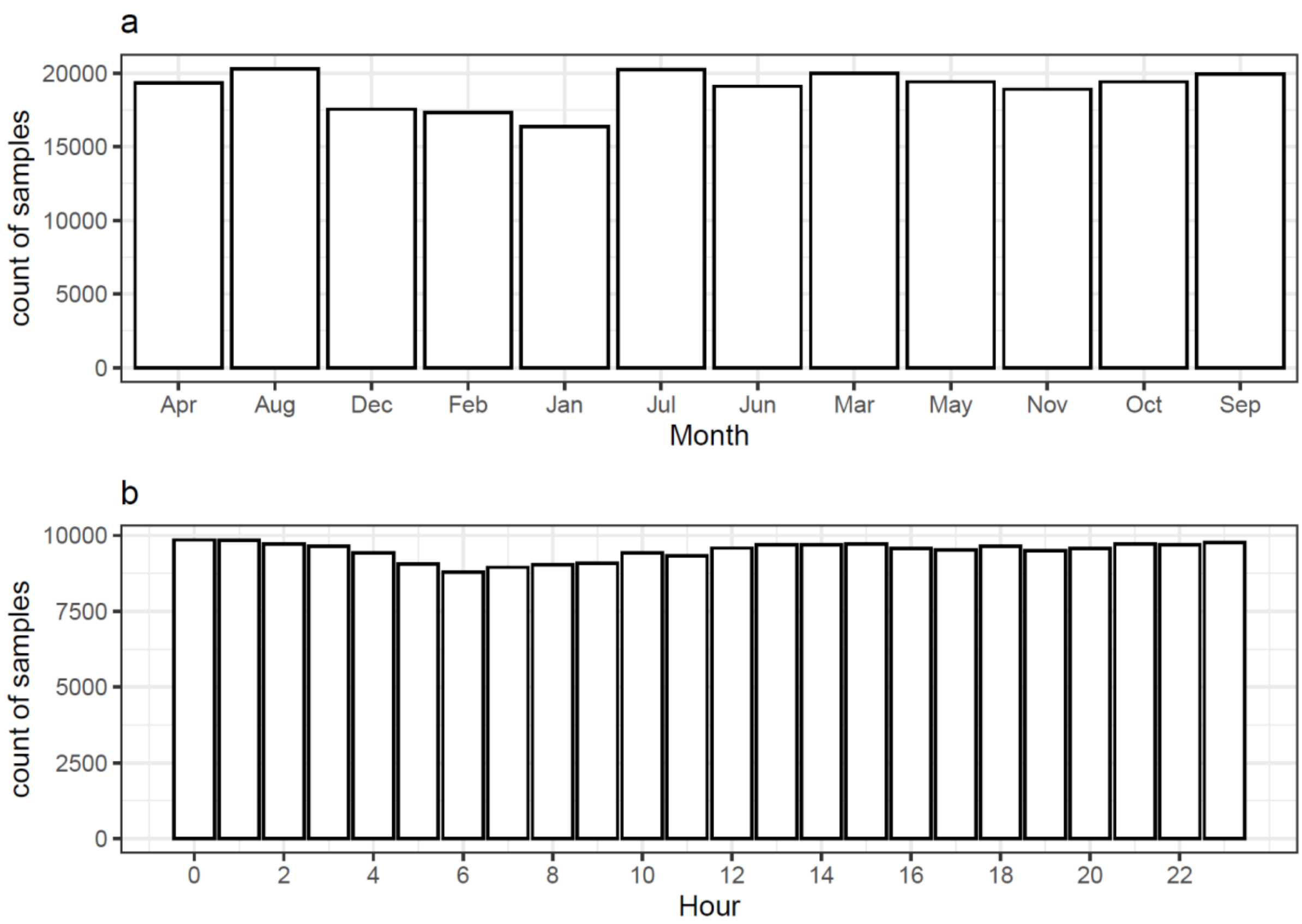

Figure S.13*:* The distribution of number of samples across months of the year (**a**) and across hours of the day (**b**)

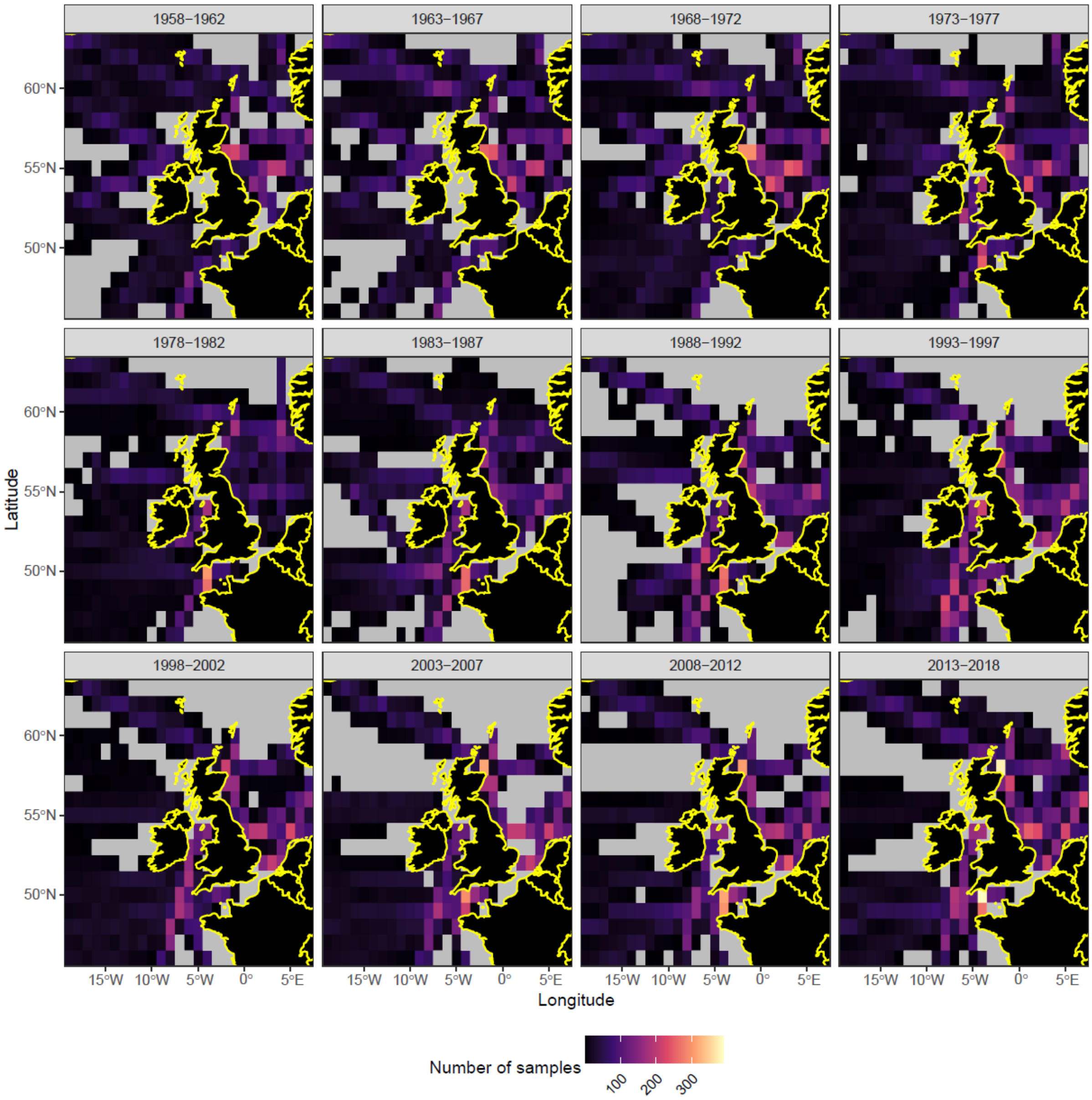

Figure S.14: Number of samples at each 5-year period. Grey cells indicate no samples.

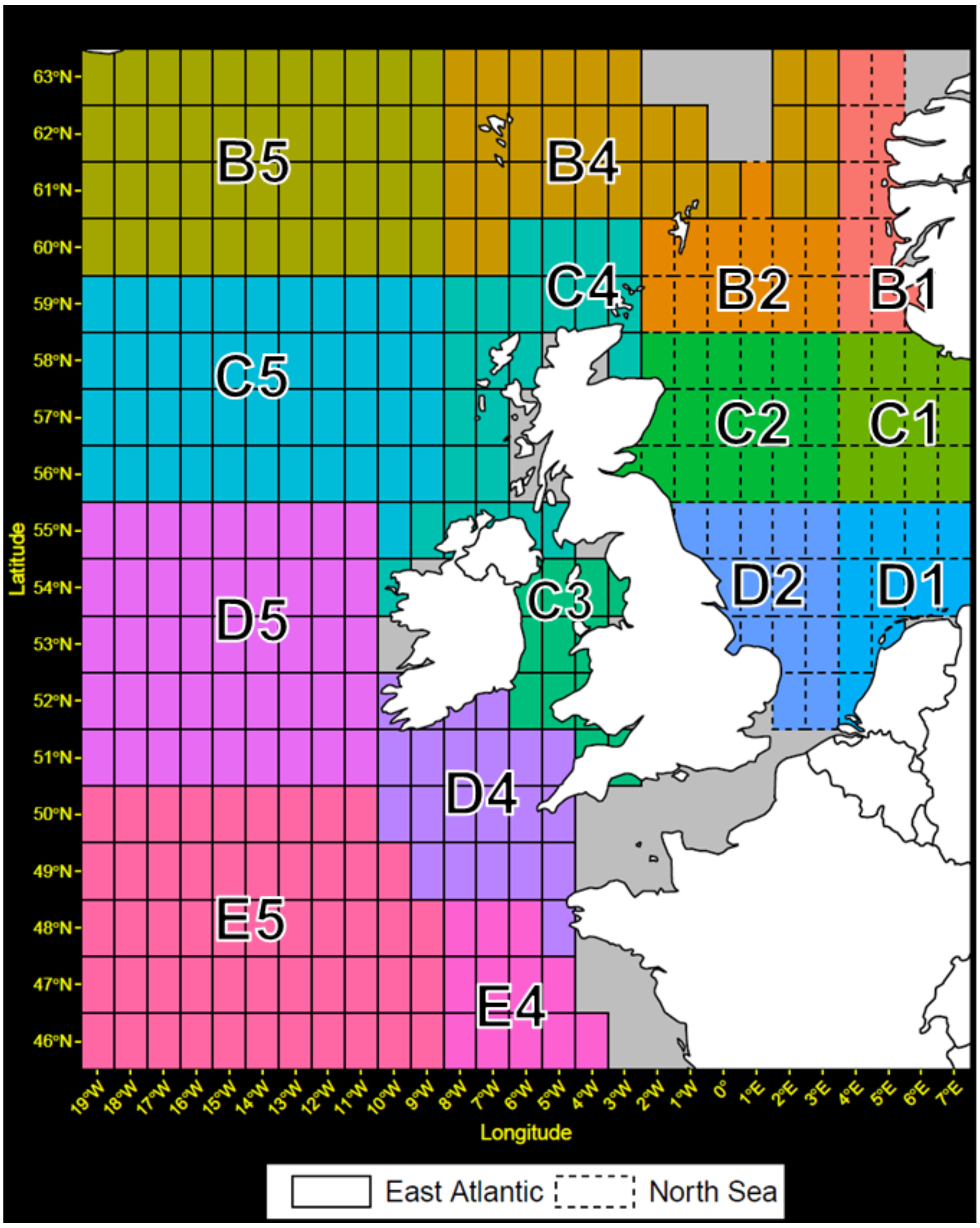

Figure S.15: Continuous plankton recorder standard areas. Straight and dashed lines indicate how we divided the North-East Atlantic and the North Sea.
