# Supplementary material for: Paradoxical regional contrasts in the resilience of marine zooplankton to climatic warming
Source: Commun Biol. 2026 Jul 17;9:1490. doi: 10.1038/s42003-026-10618-w (PMC13748652; doi:10.1038/s42003-026-10618-w)
Supplement: Supplementary file 2 [file 42003_2026_10618_MOESM2_ESM.pdf]

Description of Additional Supplementary Files

File name: Supplementary Data 1

Description: The data that support the findings of this study can be found in the Supplementary Data file
