# Supplementary material for: Paradoxical regional contrasts in the resilience of marine zooplankton to climatic warming
Source: Commun Biol. 2026 Jul 17;9:1490. doi: 10.1038/s42003-026-10618-w (PMC13748652; doi:10.1038/s42003-026-10618-w)
Supplement: Supplementary file 4 [file 42003_2026_10618_MOESM5_ESM.pdf]

nature portfolio

Peer Review File

Paradoxical regional contrasts in the resilience of marine zooplankton to climatic warming

Corresponding Author: Dr Stefano Corona

This file contains all reviewer reports in order by version, followed by all author rebuttals in order by version.

Attachments originally included by the reviewers as part of their assessment can be found at the end of this file.

Version 0:

Reviewer comments:

Reviewer #1

(Remarks to the Author)
This MS raises an important question by jointly analyzing phenological and distributional range shifts of copepods in the NE Atlantic and North Sea using the CPR dataset. The integration of these two response dimensions is a strength and provides insights of broad interest. The analyses are generally convincing, and the long-term dataset is highly appropriate for the questions posed.
However, some interpretations appear stronger than what the figures support, particularly in the discussion of isotherm tracking and "resilience" in the North Sea. The explanation of resilience as resource-based also reads somewhat single-causal, and it would be helpful to acknowledge alternative mechanisms. Overall, the study is clearly valuable, but the manuscript would benefit from refining the language to align more closely with the results, which would further strengthen its contribution.

Reviewer #2

(Remarks to the Author)
This manuscript is a well written and skillfully executed analysis of the extensive CPR dataset on copepod abundance in the Northeast Atlantic. The suggestion that both range and phenological shifts in population abundance should be analysed in concert, and the hypothesis that greater food availability in the North Sea serves to uncouple range shift in copepod abundance with shifting thermal isoclines resonates with me. This concept definitely merits consideration and would advance understanding of responses of plankton to climate warming. I support publication of the manuscript, which will stimulate to further analysis. I therefore recommend publication, but only after revision in which the authors address the following comments.

1. The role of advection.
Alternative hypotheses, not necessarily mutually exclusive, should be at least acknowledged, and I would urge also explored as explanations for differences between the NE Atlantic and North Sea. While I am not familiar with circulation patterns in detail, it appears cold water currents from the north, as well as the Scottish Coastal Current, would transport cold water species into the North Sea, counteracting the warming trend. In some ways this is analogous to the situation in the Gulf of Maine, which receives subarctic species, notably C. finmarchicus, from southerly flowing Labrador Currrent and Scotian Shelf Current carrying Gulf of St. Lawrence water (Runge et al. 2015: JPR 37(1): 221–232; Runge et al. 2025: ICES JMS: 2025, 82(4), https://doi.org/10.1093/icesjms/fsaf037). The surface circulation in the NE Atlantic, in contrast, would seem to support the northward movement of isotherms. Because of the English Channel flow of warm water into the s North Sea, I would expect that the southern N Sea to be warming much faster than the northern N. Sea and also introduce warmer species (e.g. C. helgolandicus) into the s. North Sea.

2. The role of food availability in phenological shifts
That change in food availability results in shifts in timing of seasonal abundance resonates with findings in the gulf of Maine, where there is evidence that increasing fall-winter chlorophyll concentrations and/or earlier timing of the spring bloom has stimulated earlier copepod reproduction (C. finmarchicus in spring and likely Centropages typicus in fall-winter). See Runge et al. 2025 and Honda et al. 2024: Limnol. Oceanogr: doi: 10.1002/lno.12752). Fig. S12 provides a spatial pattern of chl a

concentration, but no time series for evidence of change in food availability by season. Wouldn't the CPR data on phytoplankton functional groups and the greeness index provide an indication of chl a change, as has been done in the past (e.g. Colebrook et al. 1965; Edwards and Richardson 2004)?

Jeffrey Runge

Version 1:

Reviewer comments:

Reviewer #1

(Remarks to the Author)
Comments for the Author
The authors have responded thoughtfully to my previous comments and have made meaningful revisions throughout the MS.
Overall, the revisions have improved the clarity and balance of the paper, and the main conclusions now align well with the analyses presented. My major concerns have been addressed, and I am satisfied with the revised version of the manuscript. I believe it is suitable for publication.

Reviewer #2

(Remarks to the Author)
I have read the revision. It makes an important point and merits publication in its present form

Open Access This Peer Review File is licensed under a Creative Commons Attribution 4.0 International License, which permits use, sharing, adaptation, distribution and reproduction in any medium or format, as long as you give appropriate credit to the original author(s) and the source, provide a link to the Creative Commons license, and indicate if changes were made.
In cases where reviewers are anonymous, credit should be given to 'Anonymous Referee' and the source.
The images or other third party material in this Peer Review File are included in the article's Creative Commons license, unless indicated otherwise in a credit line to the material. If material is not included in the article's Creative Commons license and your intended use is not permitted by statutory regulation or exceeds the permitted use, you will need to obtain permission directly from the copyright holder.
To view a copy of this license, visit https://creativecommons.org/licenses/by/4.0/

Dear Editors and Reviewers,

Thank you for your thoughtful and positive assessment of our manuscript. We appreciate your time and care you put into reviewing our work. We are grateful for the constructive comments and we believe they have surely improved the quality and clarity of our work. Below, we summarise your main comments (shown in red) and provide our joint response, as they relate to similar points of discussion. More detailed, point-by-point replies to the annotated comments follow afterwards. We hope that our changes address your requests, and we remain available for any further clarification or adjustments.

Reviewer #1 (Remarks to the Author):

This MS raises an important question by jointly analyzing phenological and distributional range shifts of copepods in the NE Atlantic and North Sea using the CPR dataset. The integration of these two response dimensions is a strength and provides insights of broad interest. The analyses are generally convincing, and the long-term dataset is highly appropriate for the questions posed.

However, some interpretations appear stronger than what the figures support, particularly in the discussion of isotherm tracking and "resilience" in the North Sea. The explanation of resilience as resource-based also reads somewhat single-causal, and it would be helpful to acknowledge alternative mechanisms. Overall, the study is clearly valuable, but the manuscript would benefit from refining the language to align more closely with the results, which would further strengthen its contribution.

Reviewer #2 (Remarks to the Author):

This manuscript is a well written and skillfully executed analysis of the extensive CPR dataset on copepod abundance in the Northeast Atlantic. The suggestion that both range and phenological shifts in population abundance should be analysed in concert, and the hypothesis that greater food availability in the North Sea serves to uncouple range shift in copepod abundance with shifting thermal isoclines resonates with me. This concept definitely merits consideration and would advance understanding of responses of plankton to climate warming. I support publication of the manuscript, which will stimulate to further analysis. I therefore recommend publication, but only after revision in which the authors address the following comments.

1. The role of advection.

Alternative hypotheses, not necessarily mutually exclusive, should be at least acknowledged, and I would urge also explored as explanations for differences between the NE Atlantic and North Sea. While I am not familiar with circulation patterns in detail, it appears cold water currents from the north, as well as the Scottish Coastal Current, would transport cold water species into the North Sea, counteracting the warming trend. In some ways this is analogous to the situation in the Gulf of Maine, which receives subarctic species, notably C. finmarchicus, from southerly flowing Labrador Currrent and Scotian Shelf Current carrying Gulf of St. Lawrence water (Runge et al. 2015: JPR 37(1): 221–232; Runge et al. 2025: ICES JMS: 2025, 82(4), https://doi.org/10.1093/icesjms/fsaf037). The surface circulation in the NE Atlantic, in contrast, would seem to support the northward movement of isotherms. Because of the English Channel flow of warm water into the s North Sea, I would expect that the southern N Sea to be warming much faster than the northern N. Sea and also introduce warmer species (e.g. C. helgolandicus) into the s. North Sea.

2. The role of food availability in phenological shifts

That change in food availability results in shifts in timing of seasonal abundance resonates with findings in the gulf of Maine, where there is evidence that increasing fall-winter chlorophyll concentrations and/or earlier timing of the spring bloom has stimulated earlier copepod reproduction (C. finmarchicus in spring and likely Centropages typicus in fall-winter). See Runge et al. 2025 and Honda et al. 2024: Limnol. Oceanogr: doi: 10.1002/lno.12752). Fig. S12 provides a spatial pattern of chl a concentration, but no time series for evidence of change in food availability by season. Wouldn't the CPR data on phytoplankton functional groups and the greeness index provide an indication of chl a change, as has been done in the past (e.g. Colebrook et al. 1965; Edwards and Richardson 2004)?

We strongly agree that adding another possible explanation to the contrasting findings between the North Sea and the NE Atlantic would be not only ideal but necessary. You will see that we have now included advection and currents to the text in the Abstract e in the Discussion (line 191-201 and 243) as a possible explanation and added all the suggested references since they represent valuable supporting literature.

We thank the reviewer for the suggestion to incorporate CPR phytoplankton indices. However, we have chosen not to use the Plankton Colour Index (PCI) for this analysis, as its interpretation as a proxy for phytoplankton biomass is subject to substantial methodological uncertainties. The CPR mesh selectively retains only larger or more structurally complex phytoplankton taxa, and pigment removal to the mesh is influenced by taxon morphology, pigment content and towing speed, all of which have changed over the survey's multidecadal history. These factors introduce variability that is difficult to reconcile with quantitative estimates of food availability. Chlorophyll a, despite its own well-known limitations, is measured consistently across the full phytoplankton community and provides a more spatially and temporally comparable indicator. For this reason, we relied on the satellite-derived chlorophyll climatology presented in Figure S12 and did not extend the analysis to PCI.

Here below you will see our replies and edited new sentences to the specific comments annotated on the manuscript.

Comments at line 58: "The statement that "few studies have considered both range and phenology together" requires clarification." And "The authors argue that few studies consider both range and phenology together. Consider citing recent studies to support this statement."

Unless we are mistaken, only Chivers et al. 2017 has assessed both range and phenology shifts together on copepods, so we have clarified that in the sentence: "Even among copepods, however, studies have tended to focus either on shifts in range (Beaugrand et al. 2002) or in phenology (Mackas et al. 2012), and we are only aware of one study in which copepod phenology and range shifts are both assessed together (Chivers et al. 2017)."

Comment at line 106: "The claim that "most taxa showed an initial southward shift in the first half of the time-series, followed by a northward shift during the last decades" is not consistently supported by

Fig. 3. Please specify explicitly which taxa exhibited southward movement and temper the generalization accordingly."

Yes, that is right and we thank you for noticing this. We have now changed the text accordingly: "Abundance of the copepod taxa showed different latitudinal shift directions across the time-series in the NE Atlantic; some taxa showed an initial southward shift in the first half of the times-series (Acartia spp. and Oithona spp.), and some showed a strong northward shift during the last decades (Calanus helgolandicus, Centropages typicus, and Pseudocalanus pp.; Figure 3), whereas in the North Sea, no clear latitudinal shifts emerge"

Comment at line 116 (now 118): "The statement that 6 out of 8 taxa tracked the isotherm in the NE Atlantic is ambiguous. If "tracked" is meant as any positive northward shift, then six taxa indeed show this pattern. However, if it implies close thermal niche conservation (slope ≈ 1), only about four taxa meet this criterion, with others showing weaker or uncertain responses. Please clarify the intended definition of "tracked" and refine the wording accordingly."

Yes that is absolutely correct, only 4 taxa are significantly different from 0 but not significantly different from 1 (meaning slope =1), so only those 4 can actually be considered as tracking the isotherm. Thank you again for pointing this. We edited the sentence accordingly: "However, in the NE Atlantic the copepod abundance more closely tracked the northward-moving isotherms (abundance shifted northward at the same pace as the isotherm) than it did in the North Sea. In half of the taxa (4 out of 8) in the NE Atlantic, the northward shifts were significantly different from 0 but were not significantly different from the isotherm shift, implying thermal niche conservation (copepod abundance moved northward at the same pace as the isotherm, Figure 4)"

Comment at line 165 (now 169): "Mackas et al. (2012) suggested the idea that early-season species might advance and late-season species might delay, but their results were more mixed and taxon-specific. It would be clearer to state that your finding of uniform advancement contrasts with that expectation, rather than with their actual observations."

We rephrased the sentence as such: "We did not find the expected seasonal shift towards more "early with warming for early season species and later with warming for late season species" as expected (not found) by Mackas et al. (2012)"

Comment to line 180 (now 184): "Both regions showed northward responses, with the NE Atlantic displaying greater magnitude and consistency. Please clarify this nuance."

We rephrased the sentence: "range shifts in the NE Atlantic were much stronger in both magnitude and consistency among taxa"

Comment to line 186 (now 191): "L186–209. In my view, the explanation that higher chlorophyll-a in the southern North Sea accounts for resilience feels too single-causal. It would be stronger to discuss alternative mechanisms alongside the resource argument and indicate how your data support or contradict them."

As reported previously, we now have added the advection as another possible explanation in Discussion (Line 191-201), Abstract and Conclusion.

Comment to line 211 (now 230): “In my opinion, the conclusion may overstate resilience. The North Sea results are intriguing, but with only eight taxa the evidence seems more like potential resilience than proof of a general pattern. It would be clearer to frame this more cautiously.”

We have clarified in the sentence that this is based on just eight taxa: “In conclusion, we show highly contrasting degrees of resilience to climate warming in the eight most abundant copepod taxa over six decades”

All the other comments related to typos and formatting issues have been accounted for.

We thank you sincerely again for your review and your effort. We hope that now you will find our paper suitable for publication in Communications Biology.

With best wishes,

Stefano Corona

On behalf of all authors.

Paradoxical regional contrasts in the resilience of marine zooplankton to climatic warming

ABSTRACT

To investigate the integrated response of plankton to climate warming, we measured simultaneous shifts in abundance, geographic range and phenology of eight copepod taxa across the North Sea and NE Atlantic over 6 decades. The North Sea warmed about twice as rapidly as the NE Atlantic, yet its species showed greater resilience, maintaining more stable abundance and ranges. In contrast, the NE Atlantic experienced declining copepod populations and greater range shifts (up to 139 km northwards per decade). The greater food availability, particularly in the southern North Sea, may have contributed to this resilience. Most taxa exhibited consistent seasonal shifts with warming (up to ~39 days earlier $°C^{-1}$) in both areas. In the North Sea, species with greater range shifts also had more pronounced phenological shifts, suggesting a link between the two responses. The study highlights the need for climate-smart management that accounts for variable ecosystem resilience under rapid warming.

INTRODUCTION

In a warming world we urgently need improved projections of species distributions to better manage marine resources and appreciate how ecosystem functions will change. To predict these future patterns, a fundamental assumption of many species distribution models is that the relationship of a species distribution to specific environmental factors is broadly fixed and predictable (Cheung et al. 2008, 2009, Brun et al. 2016, Beaugrand and Kirby 2018). Thus, a species would be assumed to maintain its distribution, for instance, within the same thermal range. Populations may respond to a warming climate by adjusting their geographic range polewards or their seasonal timing (i.e.

phenology) earlier/later in the year. Shifts in range (Beaugrand et al. 2002; Parmesan and Yohe 2003; Cheung et al. 2009) and phenology (Samplonius et al. 2020) are considered to be two of the three "universal" responses to climate warming, alongside reductions in body size (Atkinson 1994; Gardner et al. 2011; Horne et al. 2017). While there is often a shift poleward or earlier in the year under warming, these shifts are by no means as smooth or predictable as often suggested (Chivers et al. 2017, Tarling et al. 2018, Atkinson et al. 2022). In models projecting future ectothermic species distributions, the fundamental assumption of a fixed thermal envelope often goes untested for the systems under study. Complementary responses, including partial resilience to change, need to be better understood for improved climate projections.

A first potential explanation for why distributional responses in space, or time, do not track climate warming is acclimatization and/or genetic adaptation to warmer conditions (niche plasticity) (Sandford and Kelly 2011; Dam 2013, Saiz et al. 2022). A second, recent, hypothesis (Vinton and Vasseur 2022) predicts that when food resources increase, both the optimal temperature and breadth of thermal performance also increase, leading to greater thermal tolerance, and hence less pressure to shift range and seasonal timing. In this sense the thermal niche is modified by changing resource abundance (e.g. food), rather than by evolution or acclimatization. If the species does not track its thermal optimum in space or time, and the two alternative mechanisms providing resilience are not possible, then the species may become increasingly disadvantaged and suffer a regional decline.

To better understand the multiple processes by which species conserve their thermal niche, or display niche plasticity, it is essential to measure distributional and abundance responses across both space and time over large spatial and temporal scales. Marine copepods are excellent candidates for this approach, since they are ectothermic (hence sensitive to thermal changes), multivoltine (multiple generations in a year) and planktonic, hence being rapidly dispersed. As such, their response can be detected temporally and geographically, often rapidly. Furthermore, most planktonic organisms are

not commercially exploited, so any long-term change that is potentially relatable to global warming is less prone to being obscured by direct human activities such as fishing (Richardson, 2008). Besides being exemplars of climate change responses, copepods comprise up to 80% of the zooplankton biomass (Humes 1994), hence are crucial to the marine trophic web, representing an important energy link between the unicellular primary producers and higher trophic levels (Atkinson, 1996).

Even among copepods, however, studies have tended to focus either on shifts in range (Beaugrand et al. 2002) or in phenology (Mackas et al. 2012), and rarely on both processes together (Chivers et al. 2017). Such an integrated approach is essential to understand how species keep pace with temperature change, both seasonally and spatially. While range shifts among marine plankton have been demonstrated (Beaugrand et al. 2002; Hays et al. 2005, Poloczanska et al. 2013), as have phenology shifts (Edwards and Richardson 2004, Mackas et al. 2012), most studies have not considered whether a species is fully tracking their thermal optimum (exhibiting thermal niche conservatism) or instead exhibiting some plasticity of their thermal niche.

In this study we analysed six decades of spatial and temporal shifts in abundance of multiple copepod species in one of the most intensively-sampled marine regions in the world. This region comprised two contrasting areas at similar latitudes, namely: a) the slowly warming (~0.08°C decade^{-1}) North-East Atlantic, and b) the very rapidly warming (~0.19°C decade^{-1}) North Sea. Comparing multiple copepod species, across two adjacent marine areas with such a different warming rate, provides an excellent natural experiment in climate change responses. By studying geographical range and phenological shifts of a suite of eight ecologically important taxa in these adjacent areas, across a 19° latitudinal range and over 58 years of warming, we aim to understand the mechanisms of thermal adjustment. Our goal is, first, to quantify the absolute change in latitudinal and seasonal distribution of copepod abundance over a multi-decadal period of warming. Second, we test whether the latitudinal distributions of each of the eight copepod taxa keep pace with the northwards shift in

isotherms (as predicted by the thermal niche conservation principle) and how these shifts relate to the phenological shift at different latitudes and in adjacent seas. Our results are paradoxical, in that the greatest geographical range shifts were in the more slowly warming of the NE Atlantic (rather than the North Sea), and we evaluate alternative hypotheses to explain instances where the thermal niche is not conserved.

RESULTS

Warming and isotherm movement

The mean SST of each consecutive five-year period has significantly increased over the years 1958 to 2018 in both the North-East Atlantic (NE Atlantic) and the North Sea (Figure 1a). The rate of increase in SST in the NE Atlantic (~0. 08°C decade^{-1}) has been slower, at around half the rate of the North Sea (0.19°C decade^{-1}). The 10, 11, 12 and 13 °C isotherms all moved north in both marine areas (Figure 1b). In the NE Atlantic, the warmer isotherms (12 and 13°C) significantly increased in latitude over time, whereas the latitude of colder isotherms (10 and 11°C) showed an initial reduction, representing cooling, between 1958 and 1987, and then a latitudinal increase, illustrating a warming period (Figure 1b). In the North Sea, the colder isotherms shifted northward by a greater latitude than did the warmer isotherms: the coldest isotherm (10 °C) shifted northward very rapidly, on average by ~0.09°N latitude degree per year (Figure 1b).

Copepod geographic range shift

Overall, the main pattern was a stronger and more consistent northward range shift response in the NE Atlantic compared to the more rapidly warming North Sea, where the populations did not track their thermal niche. Superimposed on this main pattern, mean copepod abundance tended to decrease significantly over time. Only two taxa (*Centropages typicus* and *Calanus helgolandicus*) showed any significant increase in abundance, and only in the North Sea (see Table S.1). These trends

can also be visualized in Figure 2, which compares the abundance of each taxon between the first and the last half of the whole time-series, with Figure S.1-S.8 showing more detailed maps. Abundance of the copepod taxa showed different latitudinal shift directions across the time-series in the NE Atlantic; most taxa showed an initial southward shift in the first half of the times-series, followed by a northward shift during the last decades (Figure 3). This trend broadly matches the shifting isotherms with cooling temperatures followed warming, so we calculated the latitudinal shift against time for each copepod taxon (latitude corresponding to low, medium and high abundance: quantified as the 25th, 50th and 75th quantiles respectively) during the cooling period, from 1958-1962 to 1978-1982, and during the warming period, from 1983-1987 to 2003-2007; (see Table 1).

As the 11°C isotherm moved northwards, so did the latitude at which a given percentile abundance of copepods was found, indicating a northern shift in distribution with warming (Figure 4). However, in the NE Atlantic the copepod abundance more closely tracked the northward-moving isotherms than they did in the North Sea. In most taxa (6 out of 8) the northward shifts were not significantly different from the isotherm shift in the NE Atlantic, implying thermal niche conservation (copepod abundance moved northward at the same pace as the isotherm). By contrast, the population abundance in the North Sea shifted northward with warming less intensely in all taxa, and never kept pace with the isotherm movement. Thus, the slopes of the 50th percentile of latitude for abundance against the 11°C isotherm latitude were always significantly different from 1, implying no thermal niche conservation; see Figure 4. This failure to track isotherm shifts in the North Sea was also demonstrated by the decline in percentage of copepod abundance north of the 11°C isotherm, with warming (Figure S.9). By contrast, in the NE Atlantic the changes in percentage of copepod abundance north of each isotherm were never significantly negative, implying that copepods more closely tracked the movement of the isotherms with warming. The increase or decrease of abundance with warming throughout the whole latitudinal gradient of all taxa and areas is summarized in Figure S.10, which shows that *Acartia* spp, *Oithona* spp, *Para-Pseudocalanus* spp., *Pseudocalanus* spp decreased in the south and increased in the north (although more intensely in the NE Atlantic), *C.*

helgolandicus and *Centropages typicus* instead increased across the whole latitudinal gradient, but more intensely in the North Sea.

Phenological shift

We detected a significant and consistent advancement of phenology, with shifts up to ~ 39 days earlier °C^{-1} (Figure 5). Thus, the temporal centre of gravity (COG) of species abundance became significantly earlier in the year with increasing SST (averaged between the months of April to September inclusive). This advance in seasonal timing was significant for each taxon in both areas (the only exceptions being for *Acartia* spp. and *T. longicornis* in the NE Atlantic). In the North Sea, all taxa, except *C. finmarchicus* and *C. typicus*, showed a positive relationship between phenological sensitivity to temperature (slope of COG vs. SST) and latitude, which indicates greater phenological shifts toward the early part of the year at higher latitudes (Figure 6). By contrast, in the NE Atlantic, only three taxa showed significant relationships. Thus, *Temora longicornis* and *Calanus helgolandicus* showed greater phenological shifts at northern latitudes, while *Acartia* spp. showed greater phenological shifts in the south; Figure 6. A significant relationship between phenological and latitudinal range shifts across taxa was found only for the North Sea area (Figure S.11). Thus, the thermal sensitivity of taxon-specific phenological shifts (slopes of COG vs. SST) negatively correlated with thermal sensitivity of range shifts (latitudinal shift in abundance vs. latitudinal shift of isotherm). In other words, taxa that shift more intensely in range (northward movement with warming) also shift more intensely in phenology (earlier in the year with warming).

DISCUSSION

Both the North-East Atlantic (NE Atlantic) and the North Sea have warmed between 1958 and 2018. Paradoxically, despite the North Sea warming much more rapidly than the NE Atlantic, the dominant copepods here withstood warming and retained relatively fixed geographic distributions,

unlike in the NE Atlantic where they shifted their range substantially northwards, tending to track the shifting isotherms. Phenological shifts, by contrast, appear to be much more consistent both among taxa and areas, with seasonal abundance shifting earlier with warming. Ours is one of very few studies that examines the two of these complementary adjustment mechanisms simultaneously, which is arguably needed for a fuller appreciation of temperature responses. However, the relationship between these two responses is still complex: we detected a positive relationship between phenological- and geographic range shift in the North Sea (meaning that taxa that shifted more intensely in phenology also shifted more in their geographic range, Figure S.11), however, this is based on 8 taxa, and further work is needed to explore the generality of this outcome for other taxa and areas.

We did not find the expected seasonal shift towards more "early with warming for early season species and later with warming for late season species" as suggested by Mackas et al. (2012). Nevertheless, our observed phenological shifts indicate strong tendencies towards thermal niche conservatism (Beaugrand and Kirby, 2018). Despite the fact that our results are based on monthly-resolved data, and from the near-surface layer, they are strikingly consistent: copepod seasonal distribution advances earlier in the year with warming. By contrast, phenological studies based on data from fixed stations have usually delivered statistically weaker and more mixed directions of phenological shift (Atkinson et al. 2015; Uriarte et al. 2021; Corona et al. 2024). This lack of clear phenological response at fixed stations may arise from site-specific conditions at these typically inshore locations (Uriarte et al. 2021). Continuous Plankton Recorder (CPR) data covers a much greater area of offshore regions and for much longer time series, possibly leading to better visibility of macroscale patterns such as phenology and range shifts.

A key finding of our study was the great contrast in temperature responses for the same taxa between adjacent sea areas at equivalent latitudes. Contrary to our expectation that the rapidly-warming North Sea would be a hotspot for geographical range shifts or phenological adjustment (Burrows et al. 2014, Beaugrand and Kirby, 2018), range shifts were much stronger in the NE Atlantic. Likewise, another recent study by Holland et al. (2023), comparing these same two areas but

focused on long-term plankton abundance trends instead of geographical range shifts, found much more stable abundances for major phyto- and zooplankton functional groups in the North Sea compared to the general declines seen in the NE Atlantic.

What is the cause of these counter-intuitive responses to rapid warming? Our findings of contrasting shifts between the NE Atlantic and North Sea align with the predictions of Vinton and Vasseur (2022): when resources increase, the optimal temperature and breadth of thermal performance also increase, leading to greater thermal resilience. Consequently, increased food resources in the North Sea may reduce the need for extensive adjustments in geographical range and phenology. The NE Atlantic has a more homogeneous hydrography and distribution of chlorophyll, which can sometimes even increase with increasing latitude (Campbell and Aarup, 1992). The North Sea, by contrast, has a greater chlorophyll concentration in its southern part (Quante et al. 2016), due to the different hydrographic zones (Krause et al. 1995). This is also in agreement with Hay et al. (1991) and Pitois and Fox (2006), who found a greater copepod production in the southern North Sea than in the northern part. Satellite data show that the highest chlorophyll *a* concentrations reside between 52° and 54° N in the North Sea (Figure S.12). At these latitudes, the decreases in copepod abundance with warming were less intense for most taxa in the North Sea than in the NE Atlantic (Figure S.10), implying that having a greater availability of food in the North Sea, may compensate for the increased food demand caused by warming (Litchman and Thomas 2023). We speculate that the greater chlorophyll *a* concentration in the southern North Sea leads to smaller phenological shifts, whereas the low food presence in the northern part corresponds with stronger phenological shifts, supporting the resource compensation hypothesis. However, the connection between resources and thermal sensitivity still remains to be investigated. For example, in the North Sea, phenological sensitivity to temperature becomes gradually more intense with increasing latitude, Figure 6, whereas chlorophyll *a* shows an abrupt decrease from south to north; Figure S.12. Furthermore, chlorophyll *a* is only a crude proxy for food availability, and given the debate over whether copepods are resilient or

not to warming (Beaugrand et al. 2002, Tarling et al. 2018) more work is clearly needed to explore such interactions.

In conclusion, we show highly contrasting degrees of copepod resilience to climate warming. This adds a major caveat to those models that predict responses to global warming based on fixed responses to the thermal environment, and adds to a growing body of literature suggesting variable degrees of resilience of taxa to warming (Atkinson et al. 2008, Dam 2013, Tarling et al. 2018, Saiz et al. 2022). Models of change hotspots for biota, based only on topography and the pace of temperature change (Burrows et al. 2014) may be inadequate to capture the dynamics of populations in productive areas. It is exactly these productive shelf areas, such as the North Sea, that tend to be most heavily exploited by humans, and which in our study are hotspots of apparent greater thermal resilience rather than hotspots of species replacement. "Climate smart" marine planning aims to provide protection for areas that may be vulnerable under climate change, and this relies heavily on models of future species distribution and velocities of climate change (Queirós et al. 2015, Brito-Morales et al. 2018). Our study emphasises the need for caution in such approaches (Fernandes et al 2020), especially in productive and exploited shelf regions where the biota may be most resilient to warming.

METHODS

Data collection

Copepod abundance data were obtained by the Continuous Plankton Recorder (CPR) survey, based currently at the Marine Biological Association of the UK (MBA) and published by The Archive for Marine Species and Habitats Data (https://www.dassh.ac.uk/) (Johns, 2021). The CPR devices have been in use since 1931, making it the longest-running plankton sampling survey in the world (Richardson et al. 2006). The CPR samples plankton while being towed behind ships of opportunity at a depth of ~ 6 to 7 m. Plankton are filtered by a scrolling mesh of 270 µm and sandwiched by a second mesh, before being fixed in 4% formaldehyde. Data are not collected less than 10 km from the

coastline to avoid interference from local land conditions. For our study, we used the abundance values (measured as individuals m^{-3}) of eight copepod taxa (these values refer to the late copepodite stages, see Batten et al. 2003 for more information). Four of these were identified to the species level: *Calanus finmarchicus, Calanus helgolandicus, Centropages typicus, Temora longicornis*; three taxa were identified to genus level: *Acartia* spp., *Oithona* spp., *Pseudocalanus* spp., comprising a single dominant species (respectively *Acartia clausi*, *Oithona similis* and *Pseudocalanus elongatus*); and another was a mix of *Paracalanus* spp. and *Pseudocalanus* spp. (generally referred to as "*Para-Pseudocalanus* spp."). The analysis was applied to a geographical area between 20° W and 8° E longitude and between 45° N and 64° N latitude (Figure 1), over a 60-year time span from 1958 to 2018. Sea surface temperature (SST) values were obtained by the Hadley Centre of the UK Meteorological Office (http://www.metoffice.gov.uk/hadobs/hadisst/). These monthly temperature values were taken during daylight, for a 1° latitude-longitude grid and are the product of a mix of observation and interpolation. For a more detailed description of the SST dataset and its production process see Rayner et al. (2003). Lastly, we used the chlorophyll *a* concentration time-series in order to test the possible effect of food on the copepod phenological sensitivity change across latitudes. Chlorophyll data were extracted for the same marine areas as the CPR survey. The regional monthly chlorophyll climatology maps are calculated over the 1997–2018 period by averaging monthly chlorophyll values on a pixel-by-pixel basis. Both the time series and the climatology maps were calculated using the global merged multi-sensor product OCEANCOLOUR_GLO_CHL_L3_REP_OBSERVATIONS_009_065 distributed by the Copernicus Marine Environment Monitoring Service (https://marine.copernicus.eu/). This product was derived from the OC-CCI v4.0 data set produced by the ESA Ocean Colour Climate Change Initiative (ESA OC-CCI, Sathyendranath et al., 2018).

Data extraction

Data analysis followed the general approach of Chivers et al. (2017). Copepod abundance and SST data were averaged by year and by each 1° latitude ×1° longitude section from the area between

45°N and 64°N latitude, and between 20°W and 8°E longitude. The number of samples (equal for each taxon) was very similar for each month of the year (mean count: ~18892; SD: 1203) and each hour of the day (mean count: ~9491; SD: 292), hence showing no major biases toward a certain part of the year, or time of the day (see Figure S.13). We interpolated copepod abundance by kriging the five-year averages of ln(Abundance + 1) values for each taxon (following the method in Chivers et al. 2017). To avoid potential biases, we only considered grid cells that were sampled in more than 25% of all sampling months over the whole time series (for geographical data coverage, see Figure S.14). Kriging was performed using packages "sp" (Pebesma and Bivand 2005), "gstat" (Pebesma 2004) and "automap" (Hiemstra et al. 2009) in R. The former package was used to determine the best fitting variogram by using the autovariogram function for each species and each five-year period. The sea surface temperature (SST) values were used to calculate the latitude of the isotherms at 10, 11, 12 and 13 °C for each 1° longitude in each five-year period (these isotherms were chosen to straddle the centre of the geographic area and taxon ranges sampled, see Figure 2). To do this, we performed polynomial regressions between the averaged SST against latitude at each longitude degree. We subdivided the area into two parts: the North-East Atlantic (NE Atlantic), which comprises the CPR areas B4, B5, C3, C4, C5, D4, D5, E4, E5, and the North Sea (North Sea), which comprises the CPR areas B1, B2, C1, C2, D1, D2 (see Figure S.15). The same kriging method was applied to the chlorophyll *a* dataset as that used for copepod abundance. We $\log_{10}$-transformed the concentration values (calculated as mg m^{-3}) and averaged the overall (across all years from 1998 to 2018) abundance between and including April and September, for each latitudinal degree, in order to test whether the different amount of food across latitudes would match the latitudinal difference in phenological sensitivity.

Range shift analysis

First, we assessed the latitudinal change in SST over time, by regressing the average latitude of each isotherm against year (averaging across all longitudes). Then, in order to estimate the copepod

geographic range shift, we calculated the latitude corresponding to low, medium and high abundance for each copepod taxon (i.e. 25th, 50th and 75th quantiles of copepod abundance) in the two distinct marine areas (NE Atlantic and North Sea), across all longitudes, using the function wtd.quantile in R (package: Hmisc (Harrell 2021)). We regressed these latitudes for each measure of copepod abundance against a measure of SST (the latitude of the 11°C isotherm, chosen because it covers both the NE Atlantic and the North Sea). We then calculated the average percentage copepod abundance north of each isotherm (relative to the abundance south of the same isotherm) for each five-year period and for each taxon, following Chivers et al., (2017). These percentage values were linearly regressed against the mean latitude of each corresponding isotherm, to determine whether copepods changed their latitudinal distribution at the same rate as the isotherms shift with warming. To avoid potential biases related to the different latitudinal ranges between the two marine areas, we repeated these analyses by considering the same latitudinal range (between 52° and 63° N) for both areas.

Phenology analysis

To calculate the phenological shift, we first applied kriging interpolation for the abundance of each taxon for each five-year period for each month. From these values we then calculated the centre of gravity (measured as month of the year) of abundance for each year across the whole geographical range. The centre of gravity method allows us to determine in which part of the year the copepod abundance is centred, i.e. earlier or later in the year; see Colebrook & Robinson, 1963; Edwards & Richardson, 2004; Mackas et al. 2012). The centre of gravity C (in Julian months) was calculated using the following formula:

$$C = \sum(N_M * M) / \sum N_M$$

Where N_M is the abundance of each taxon at each month M. We regressed the centre of gravity indices against yearly SST (average between April and September) to measure the long-term phenological shift (we than repeated the same analysis for each latitudinal degree). We then regressed taxon-

specific latitudinal shifts against phenological shifts, in order to detect possible correlations between the two responses to climate change (latitudinal and phenological shifts).

REFERENCE LIST

1. Atkinson, A. (1996). Subantarctic copepods in an oceanic, low chlorophyll environment: ciliate predation, food selectivity, and impact on prey populations. *Marine Ecology Progress Series*, 130, 85–96.
2. Atkinson, A., Siegel, V., Pakhomov, E. A., Rothery, P., Loeb, V., Ross, R. M., Quetin, L. B., Fretwell, P., Schmidt, K., Tarling, G. A., Murphy, E. J., Fleming, A. (2008). Oceanic circumpolar habitats of Antarctic krill. *Marine Ecology Progress Series*, 362, 1–23.
3. Atkinson, A., Harmer, R. A., Widdicombe, C. E., McEvoy, A. J., Smyth, T. J., Cummings, D. G., Somerfield, P. J., Maud, J. L., McConville, K. (2015). Questioning the role of phenology shifts and trophic mismatching in a planktonic food web. *Progress in Oceanography*, 137, 498–512. https://doi.org/10.1016/j.pocean.2015.04.023
4. Atkinson, A., et al. (2022). Stepping stones towards Antarctica: Switch to southern spawning grounds explains an abrupt range shift in krill. *Global Change Biology*, 28(4), 1359–1375. https://doi.org/10.1111/gcb.16009
5. Atkinson, D. (1994). Temperature and organism size—A biological law for ectotherms? *Advances in Ecological Research*, 25(C), 1–58. https://doi.org/10.1016/S0065-2504(08)60212-3
6. Batten, S. D., et al. (2003). CPR sampling: The technical background, materials and methods, consistency and comparability. *Progress in Oceanography*, 58, 193–215.
7. Beaugrand, G., Reid, P. C., Ibanez, F., Lindley, J., Alistair, Edwards, M. (2002). Reorganization of North Atlantic marine copepod biodiversity and climate. *Science*, 296(5573). https://doi.org/10.1126/science.1071329
8. Beaugrand, G. & Kirby, R. R. (2018). How do marine pelagic species respond to climate change? Theories and observations. *Annual Review of Marine Science*, 10(1), 169–197. https://doi.org/10.1146/annurev-marine-121916-063304
9. Brito-Morales, I, et a. (2018). Climate velocity can inform conservation in a warming world. *Trends in Ecology and Evolution*, 33, 441–457. https://doi.org/10.1016/j.tree.2018.03.009
10. Brun, P., Payne, M. R., Kiørboe, T. (2016). Trait biogeography of marine copepods—An analysis across scales. *Ecology Letters*, 19, 1403–1413. https://doi.org/10.1111/ele.12688
11. Burrows, M. T., Schoeman, D. S., Richardson, A. J., Molinos, J. G., Hoffmann, A., Buckley, L. B., Moore, P. J., Brown, C. J., Bruno, J. F., Duarte, C. M., Halpern, B. S. (2014). Geographical limits to species-range shifts are suggested by climate velocity. *Nature*, 507(7493), 492–495.
12. Campbell, J. W. & Aarup, T. (1992). New production in the North Atlantic derived from seasonal patterns of surface chlorophyll. *Deep Sea Research Part A:*

Oceanographic Research Papers, 39(10), 1669–1694. https://doi.org/10.1016/0198-0149(92)90023-M
13. Cheung, W. W. L., Close, C., Lam, V., Watson, R., Pauly, D. (2008). Application of macroecological theory to predict effects of climate change on global fisheries potential. *Marine Ecology Progress Series*, 365, 187–197. https://doi.org/10.3354/meps07414
14. Cheung, W. W. L., Lam, V. W. Y., Sarmiento, J. L., Kearney, K., Watson, R., Pauly, D. (2009). Projecting global marine biodiversity impacts under climate change scenarios. *Fish and Fisheries*, 10(3), 235–251. https://doi.org/10.1111/j.1467-2979.2008.00315.x
15. Chivers, W. J., Walne, A. W., Hays, G. C. (2017). Mismatch between marine plankton range movements and the velocity of climate change. *Nature Communications*, 8. https://doi.org/10.1038/ncomms14434
16. Colebrook, J. M. & Robinson, G. A. (1963). Continuous plankton recorder records: Seasonal cycles of phytoplankton and copepods in the northeastern Atlantic and the North Sea. *Bulletins Marine Ecology*, 6, 123–139.
17. Corona, S., Hirst, A. G., Atkinson, D., Renz, J., Boersma, M., Atkinson, A. (2024). Long-term shifts in phenology, thermal niche, population size, and their interactions in marine pelagic copepods. *Limnology and Oceanography*, 9999, 1–16. https://doi.org/10.1002/lno.12499
18. Johns, D. (2021). Selected CPR zooplankton data—All standard areas 1958 to 2018. *The Archive for Marine Species and Habitats Data (DASSH)*. https://doi.org/10.17031/1767
19. Dam, H. G. (2013). Evolutionary adaptation of marine zooplankton to global change. *Annual Review of Marine Science*, 5, 349–370. https://doi.org/10.1146/annurev-marine-121211-172229
20. Edwards, M. & Richardson, A. J. (2004). Impact of climate change on marine pelagic phenology and trophic mismatch. *Nature*, 430(7002), 881–884. https://doi.org/10.1038/nature02808
21. Fernandes, J. A., Rutterford, L., Simpson, S. D., Butenschön, M., Frölicher, T. L., Yool, A., Cheung, W. W. L., Grant, A. (2020). Can we project changes in fish abundance and distribution in response to climate? *Global Change Biology*, 26, 3891–3905. https://doi.org/10.1111/gcb.15081
22. Gardner, J. L., Peters, A., Kearney, M. R., Joseph, L., Heinsohn, R. (2011). Declining body size: A third universal response to warming? *Trends in Ecology and Evolution*, 26(6), 285–291. https://doi.org/10.1016/j.tree.2011.03.005
23. Harrell, F. E. Jr. (2021). Hmisc: Harrell Miscellaneous. *R package version 4.6-0*. https://CRAN.R-project.org/package=Hmisc
24. Hay, S. J., Kiørboe, T., Matthews, A. (1991). Zooplankton biomass and production in the North Sea during the Autumn Circulation Experiment, October 1987–March 1988. *Continental Shelf Research*, 11(12), 1453–1476. https://doi.org/10.1016/0278-4343(91)90021-W
25. Hays, G. C., Richardson, A. J., Robinson, C. (2005). Climate change and marine plankton. *Trends in Ecology and Evolution*, 20(6). https://doi.org/10.1016/j.tree.2005.03.004
26. Hiemstra, P. H., Pebesma, E. J., Twenhöfel, C. J. W., Heuvelink, G. B. M. (2009). Real-time automatic interpolation of ambient gamma dose rates from the Dutch radioactivity monitoring network. *Computers & Geosciences*, 35, 1711–1721.

27. Holland, M. M., et al. (2023). Major declines in NE Atlantic plankton contrast with more stable populations in the rapidly warming North Sea. *Science of the Total Environment*, 898, 165505. https://doi.org/10.1016/j.scitotenv.2023.165505
28. Horne, C. R., Hirst, A. G., Atkinson, D. (2017). Seasonal body size reductions with warming covary with major body size gradients in arthropod species. *Proceedings of the Royal Society B*, 284(1855), 20170704. https://doi.org/10.1098/rspb.2017.0704
29. Humes, A. G. (1994). How many copepods? *Hydrobiologia*, 292–293(1), 1–7. https://doi.org/10.1007/BF00229916
30. Krause, M., Dippner, J. W., & Beil, J., (1995). A review of hydrographic controls on the distribution of zooplankton biomass and species in the North Sea with particular reference to a survey conducted in January-March 1987. *Progress in Oceanography*, 35(2), 81–152. https://doi.org/10.1016/0079-6611(95)00006-3
31. Litchman, E. & Thomas, M.K., (2023). Are we underestimating the ecological and evolutionary effects of warming? Interactions with other environmental drivers may increase species vulnerability to high temperatures. *OIKOS*. 2023(2). https://doi.org/10.1111/oik.09155
32. Mackas, D. L., et al. (2012). Changing zooplankton seasonality in a changing ocean: Comparing time series of zooplankton phenology. *Progress in Oceanography*, 97–100, 31–62. https://doi.org/10.1016/j.pocean.2011.11.005
33. Parmesan, C., & Yohe, G. (2003). A globally coherent fingerprint of climate change. Nature, 421, 37–42. https://doi.org/10.1038/nature01286
34. Pebesma, E. J. (2004). Multivariable geostatistics in S: the gstat package. *Computers & geosciences*, 30(7), 683-691.
35. Pebesma, E., & Bivand, R. S. (2005). S classes and methods for spatial data: the sp package. *R news*, *5*(2), 9-13.
36. Pitois, S. G., Fox, C. J., (2006). Long-term changes in zooplankton biomass concentration and mean size over the Northwest European shelf inferred from Continuous Plankton Recorder data. *ICES Journal of Marine Science*, 63(5), 785–798. https://doi.org/10.1016/j.icesjms.2006.03.009
37. Poloczanska, E. S., et al. (2013). Global imprint of climate change on marine life. *Nature Climate Change*, 3(10), 919–925. https://doi.org/10.1038/nclimate1958
38. Quante, M., & Colijin, F. (2016). North Sea Region Climate Assessment, NOSCCA(October). https://doi.org/10.1007/978-3-319-39745-0
39. Queiros, S. M., et al. (2015). The long-term dynamics of marine ecosystems. *Environmental Monitoring and Assessment*, 187(7), 123-134. https://doi.org/10.1007/s10661-015-4730-3
40. Rayner, N. A. et al. (2003). Global analyses of sea surface temperature, sea ice, and night marine air temperature since the late nineteenth century. *J. Geophys. Res. Atmos,* 108, 4407.
41. Richardson, A. J., et al. (2006). Using continuous plankton recorder data. *Progress in Oceanography*, 68(1), 27–74. https://doi.org/10.1016/j.pocean.2005.09.011. https://doi.org/10.1126/science.1095339
42. Richardson, A. J. (2008). In hot water: zooplankton and climate change. *ICES Journal of Marine Science*, 65(3), 279–295. https://doi.org/10.1093/icesjms/fsn028
43. Saiz, E., Griffell, K., Olivares, M., Solé, M., Theodorou, I., & Calbet, A. (2022). Reduction in thermal stress of marine copepods after physiological acclimation. *Journal of Plankton Research*, 44(3), 427–442. https://doi.org/10.1093/plankt/fbac017
44. Samplonius J.M., et al. (2020). Strengthening the evidence base for temperature-mediated phenological asynchrony and its impacts. *Nature Ecology and Evolution* 5, 155–164. https://doi.org/10.1038/s41559-020-01357-0

45. Sanford, E., & Kelly, M. W. (2011). Local adaptation in marine invertebrates. *Annual Review of Marine Science*, 3, 509–535. https://doi.org/10.1146/annurev-marine-120709-142756
46. Sathyendranath, S., et al. (2018): ESA Ocean Colour Climate Change Initiative (Ocean_Colour_cci): Version 3.1 Data. Centre for Environmental Data Analysis, 04 July 2018. doi:10.5285/9c334fbe6d424a708cf3c4cf0c6a53f5.
47. Tarling, A., Ward, P., Thorpe, E. S. (2018). Spatial distributions of Southern Ocean mesozooplankton communities have been resilient to long-term surface warming. *Global Change Biology,* 24(1), 132-142. https://doi.org/10.1111/gcb.13834
48. Vinton, A. C., & Vasseur, D. A. (2022). Resource limitation determines realized thermal performance of consumers in trophodynamic models. *Ecology Letters*, 25(10), 2142–2155. https://doi.org/10.1111/ele.14086

Acknowledgements

We thank the owners and crews that have towed the CPRs on a voluntary basis for over 80 years contributing to one of the world's largest and longest ongoing ecological experiments as well as the countless active and retired analysts. We thank David Johns for supplying these CPR data, and Ben Howey, who kindly provided the OC-CCI gridded Chlorophyll *a* products.

Competing interests

The authors declare no competing financial interests.

Funding: S.C. was funded by a PhD award from the School of Environmental Sciences, University of Liverpool to AGH; UK Natural Environment Research Council (NERC) FOCUS and AtlantiS programmes (A.A)

Material & Correspondence

For any material request refer to the corresponding author.

Data availability.

The data that support the findings of this study are publicly available upon request:

1. The plankton population data are available from The Uk Archive for Marine Species and Habitats Data (DASSH): https://dassh.ac.uk/

2. The temperature data are available from the Hadley Centre Sea Ice and Sea Surface Temperature data set (HadISST): http://www.metoffice.gov.uk/hadobs/hadisst/

3. The Chlorophyll *a* data are available from the Copernicus Marine Environment Monitoring Service (CMEMS): https://marine.copernicus.eu/it

Tables

Table 1: Total movement (km) during the cooling period (from 1958-1962 to 1978-1982) and warming period (from 1983-1987 to 2003-2007) for each taxon in each of the two areas. Total movement is calculated as the mean of all three quantiles (25th, 50th, 75th) of latitude for copepod abundance vs. time (five-year periods) and then converted from latitudinal degree (°N) into km. Positive values indicate northward movement and negative values indicate southward movement.

	**North-East Atlantic**		**North Sea**	
**Taxon**	**Total movement (km) - cooling period**	**Total movement (km) - warming period**	**Total movement (km) - cooling period**	**Total movement (km) - warming period**
Acartia spp.	-173.90	184.70	-24.37	20.35
Calanus finmarchicus	136.90	222.00	3.70	151.98
Calanus helgolandicus	-199.80	94.91	-2.38	51.80
Centropages typicus	-17.96	-139.19	-49.95	57.35
Oithona spp.	-186.85	-90.65	7.40	-33.30
Para-Pseudocalanus spp.	-83.25	146.05	12.03	16.65
Pseudocalanus spp.	-142.45	347.80	-12.95	29.60
Temora longicornis	-442.15	-101.75	-12.95	55.48

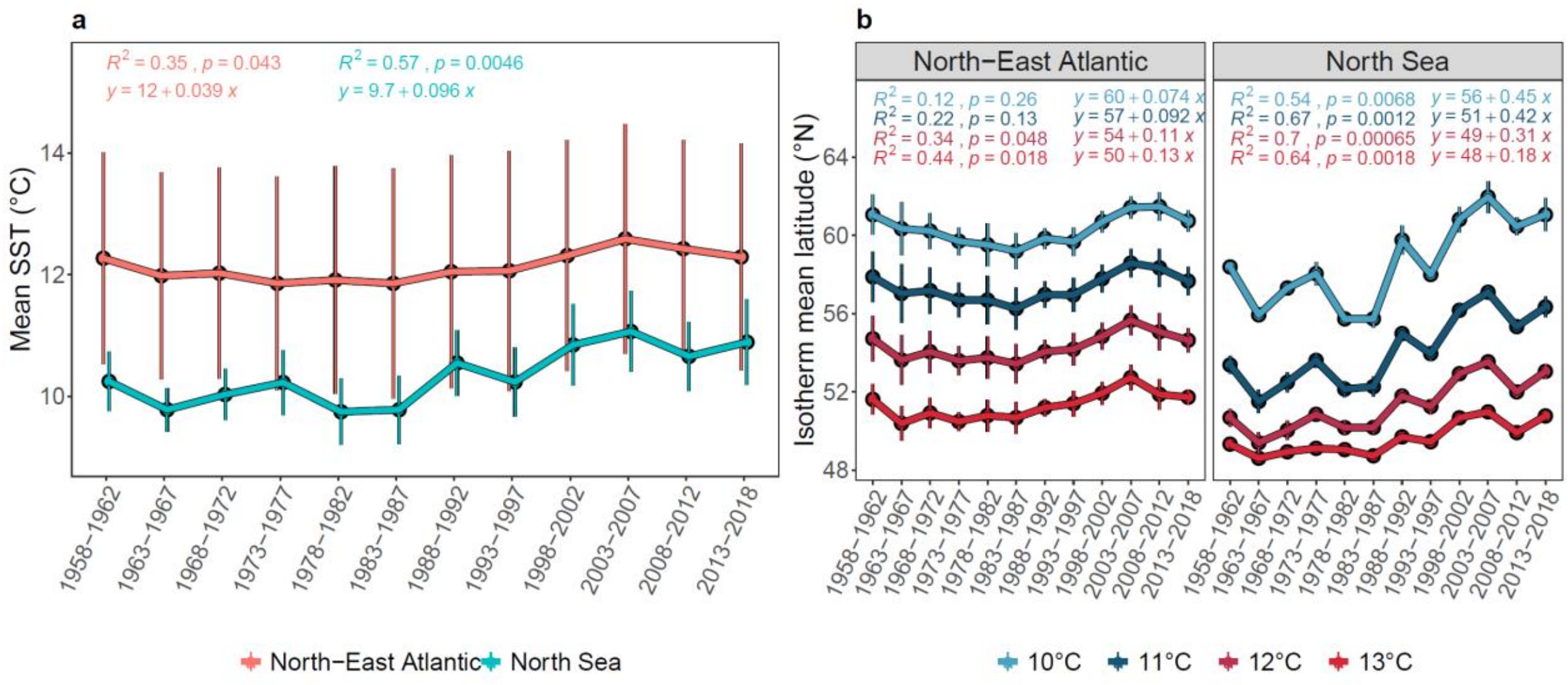

Figure 1: The North Sea has warmed much faster than the NE Atlantic. **a**) Mean of the estimated SST values over the twelve five-year periods. Colours refer to the two different marine areas considered as indicated by the legend at the bottom. **b**) Mean latitudes of the 10, 11, 12 and 13 °C isotherms (colour indexed as indicated by the legend at the bottom) for each five-year period. The R^2, p-value and the equation from the linear regressions between the temperature on the y-axis (indexed by mean SST (°C) in **a)** and mean isotherm latitude (°N) in **b)**) and time on the x-axis (indexed by 5-year groups).

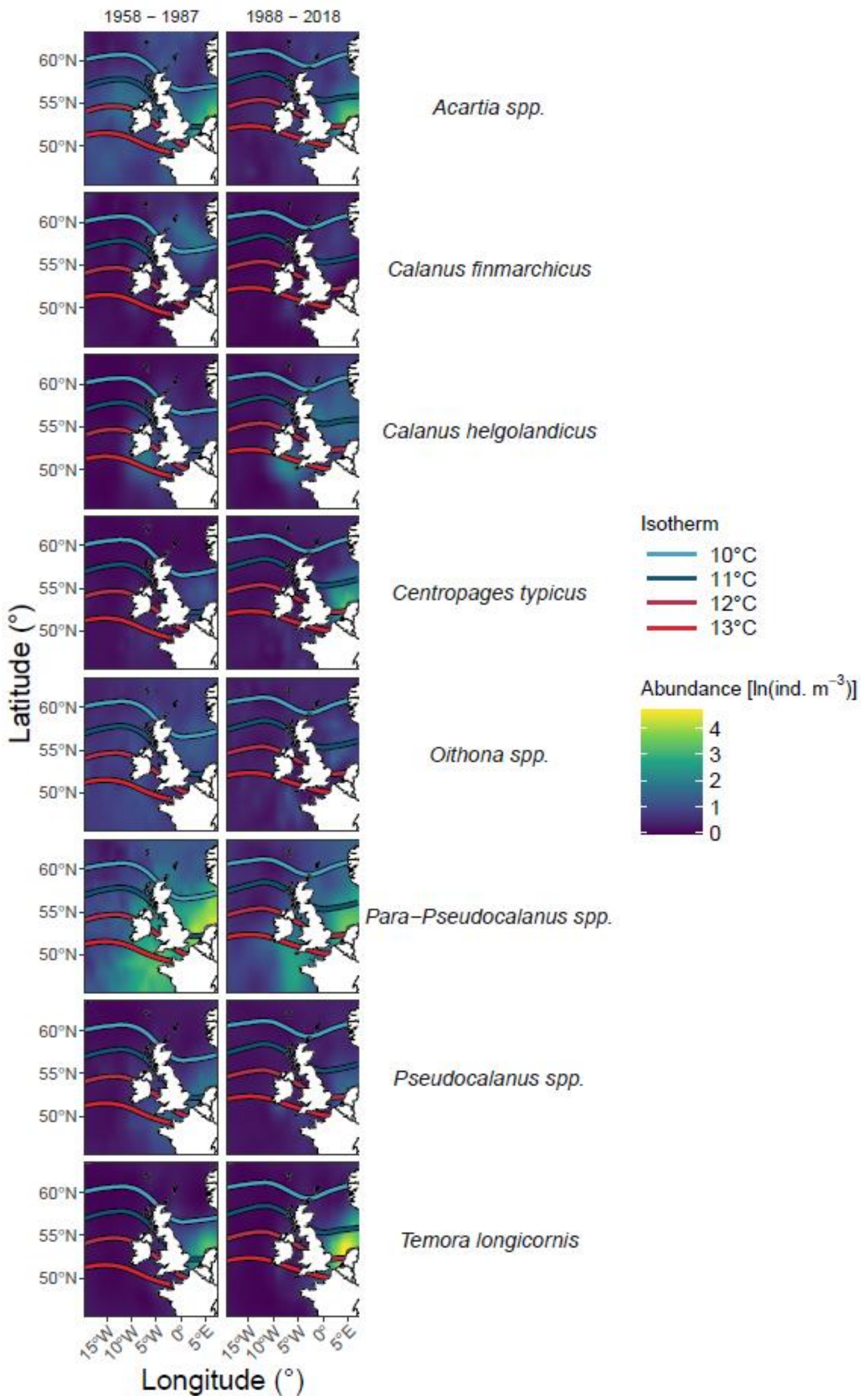

Figure 2: Geographic range shifts vary greatly between taxa. Geographic distribution of each copepod taxon in the whole area during the first (1958 – 1987) and last (1988 – 2018) 30 years of the time series. Bright areas (yellows and greens) indicate high copepod abundance (ln(x+1)) as indicated

by the legend on bottom-right. Coloured lines indicate the four isotherms at different temperatures (10, 11, 12, 13 °C) as indicated by the legend on top-right.

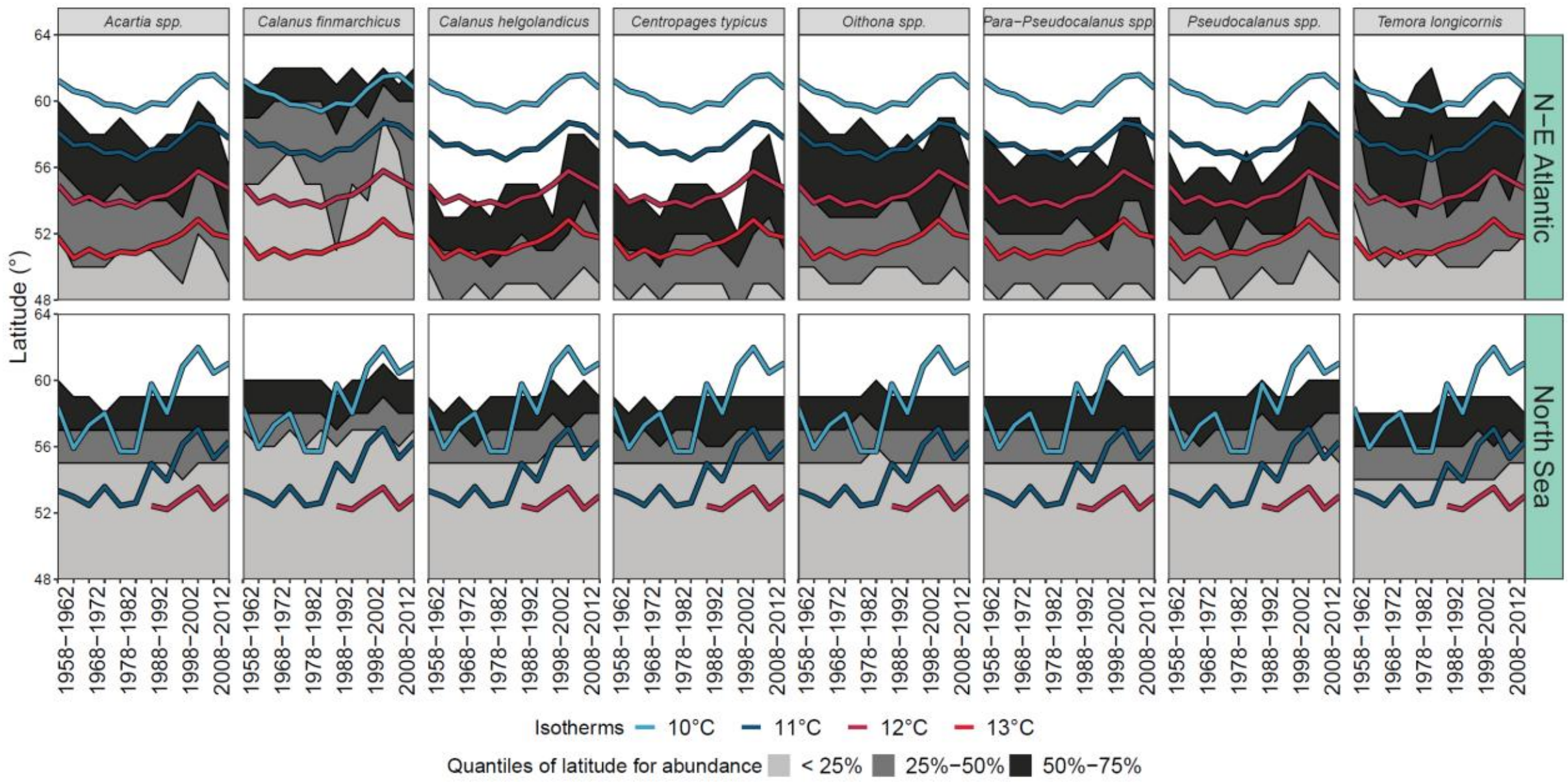

Figure 3: Copepod distributions in the NE Atlantic have shifted with the isotherms, but in the North Sea they have remained relatively fixed despite its faster warming. Long-term trends of the 25%, 50% and 75% quantiles of latitude for copepod abundance indicated by the three coloured bands. Superimposed lines indicate the long-term trend in the latitude of isotherms (colour indexed for four different temperatures as indicated by the legend on the bottom).

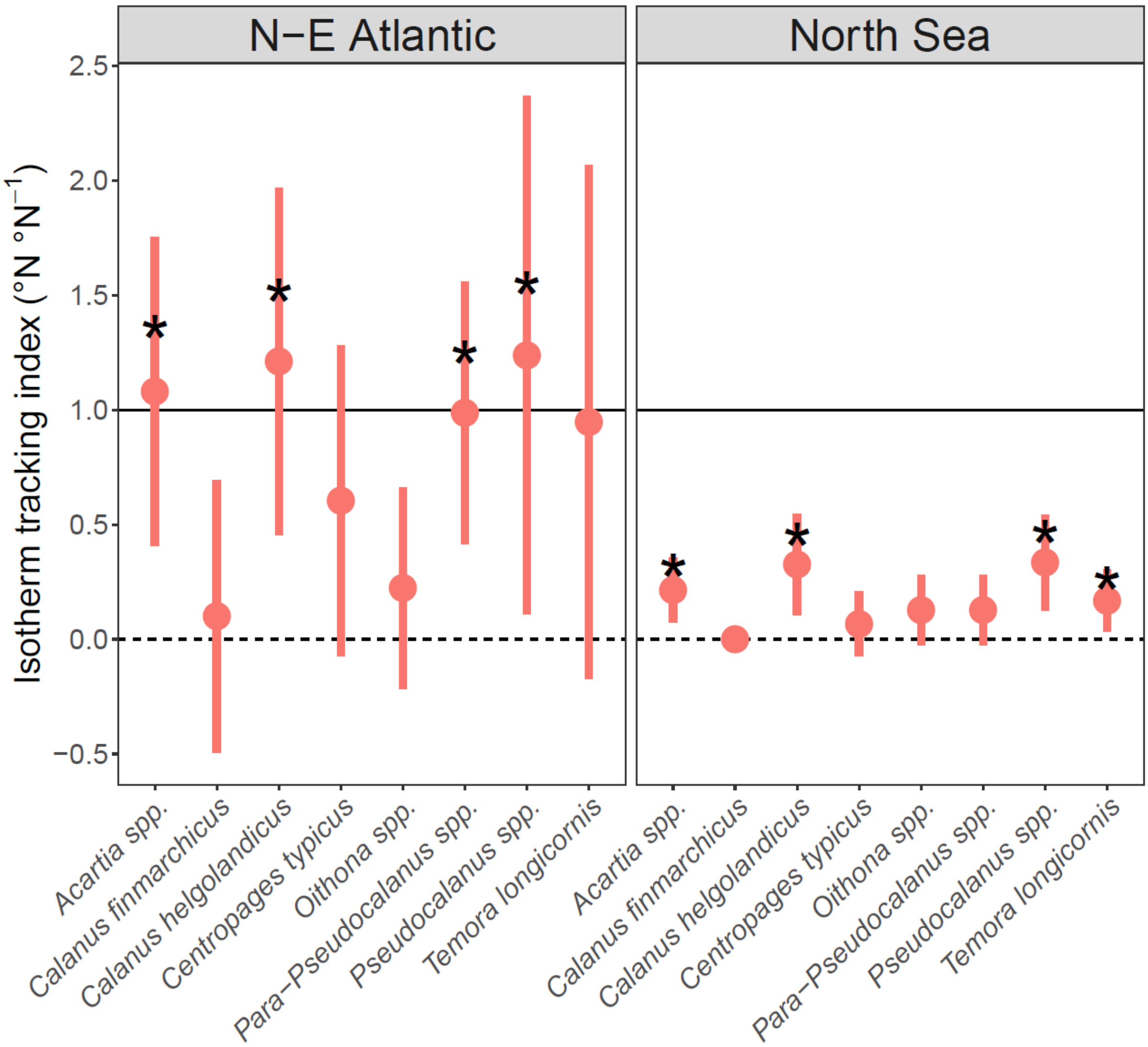

Figure 4: Copepods shift northward in both marine areas, but in the North Sea they track the isotherm shift less well. Summary of the taxon-specific linear regression slopes across twelve 5-year periods between 1958 and 2018 (n = 12) for latitude of copepod 50th percentile abundance vs. mean latitude of the 11°C isotherm (°N °N^{-1}) of each area. The 95% C.I. of each slope is represented as vertical error bars in each point. At y (isotherm tracking index) = 0- (dashed horizontal line) copepod abundance shows no latitudinal shift. Asterisks indicate significant increase in latitude of 50% abundance per shift in mean latitude of the 11 °C isotherm (* = $p \leq 0.05$). Aty = 1 (solid horizontal line) the latitudinal shift of copepod abundance matches the latitudinal shift of the isotherm (indicating thermal niche tracking).

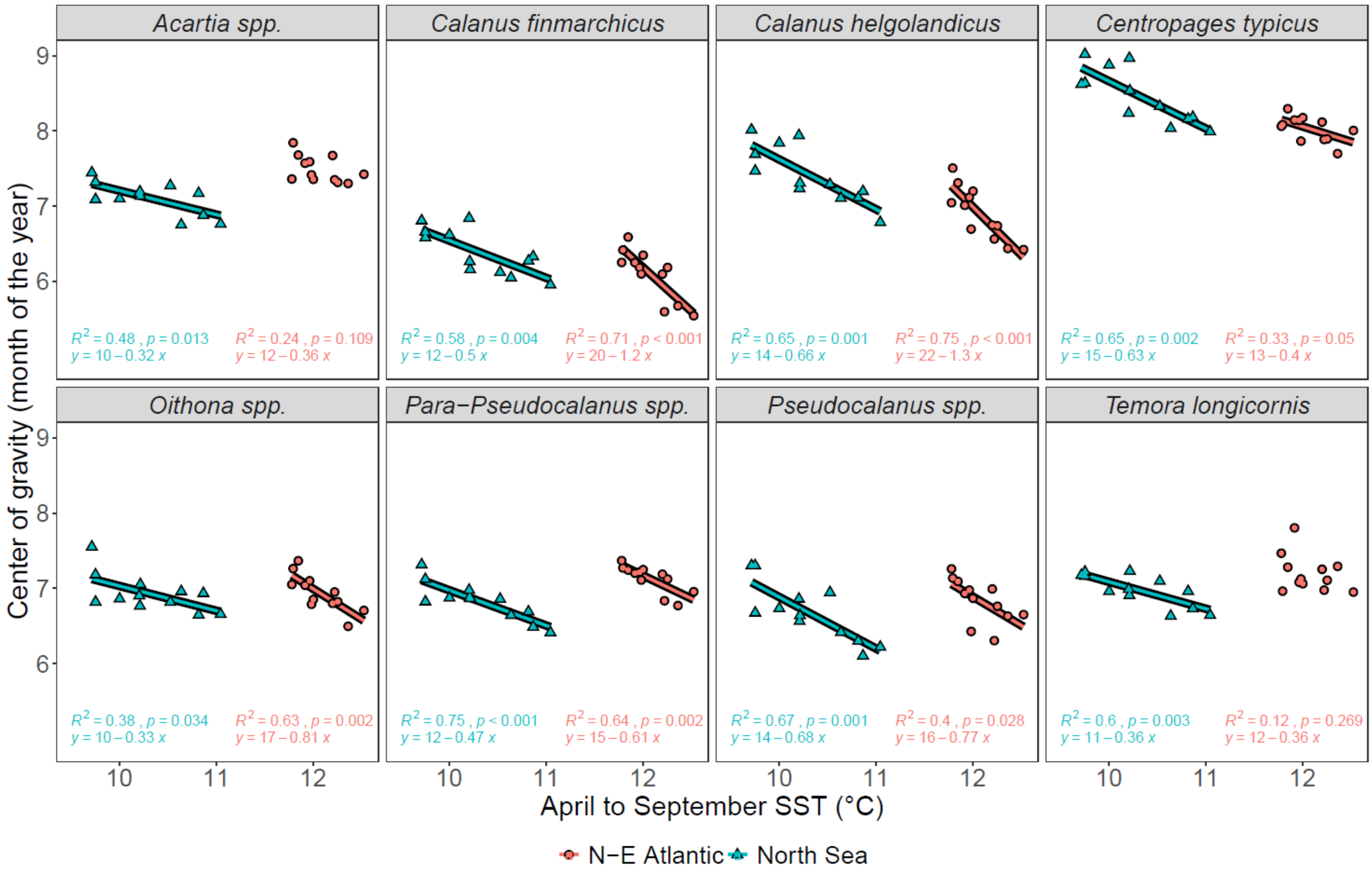

Figure 5: The phenology of copepods tends to shifts earlier in the year in both the North Sea and the North-East Atlantic. Linear regressions between the centre of gravity (COG) of abundance (expressed as Julian month) and the sea surface temperature (SST) between April and September (both included) for all taxa. Each point represents a 5-year period across 60 years. Negative slopes indicate a shift of abundance seasonal distribution toward the early part of the year with warming. At the bottom of each panel R^2, p-value and linear equations for each regression are reported. Fitted lines are only included when the relationship is significant ($p<0.05$).

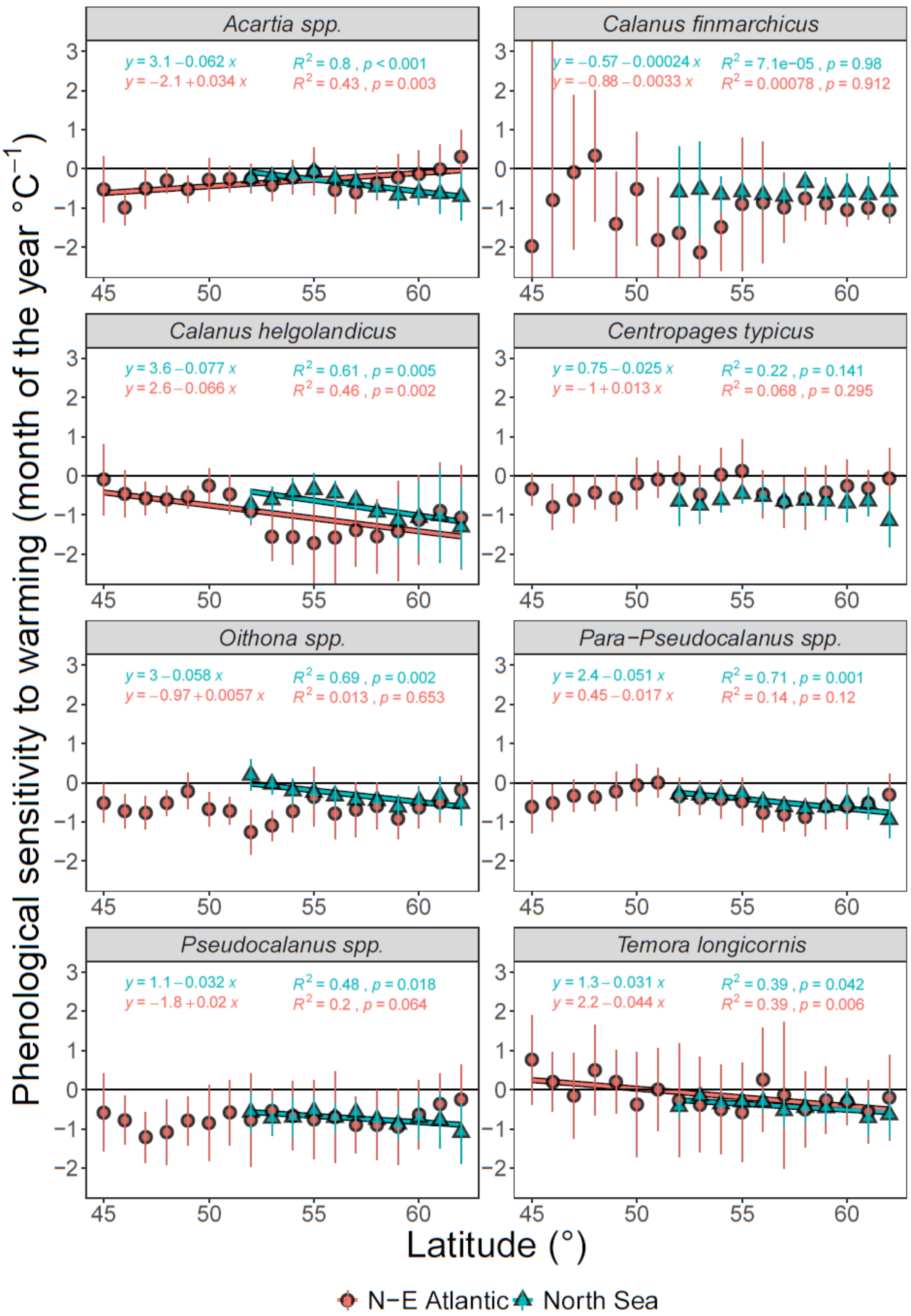

Figure 6: Phenological sensitivity to temperature increases with latitude in the North Sea. Distribution of phenological sensitivity to temperature values (slopes from the regressions between centre of gravity and temperature; with negative values indicating earlier phenology with warming, see Figure 7) on the y-axis at each latitudinal degree on the x-axis. Colours and shape of points refer

to the two marine areas as indicated by the legend at the bottom. Regressions across all data in the plots are fitted and reported when significant (for N-E Atlantic and the North Sea separately). Vertical lines for each point indicate the 95% C.I. of each slope, when these lines do not intersect the horizontal black line they indicate the slope is significant.

SUPPLEMENTARY INFORMATION

Table S.1: Slopes (β), p and R^2 values of each linear regression of mean abundance [i.e. ln(x+1)] vs. time (5-year periods, n=12).

Taxon	Marine area	β	*p*	R^2
Acartia spp.	N-E Atlantic	**-0.25**	**0.00**	**0.69**
Calanus finmarchicus	N-E Atlantic	-0.03	0.10	0.24
Calanus helgolandicus	N-E Atlantic	0.05	0.13	0.22
Centropages typicus	N-E Atlantic	0.06	0.19	0.17
Oithona spp.	N-E Atlantic	**-0.21**	**0.00**	**0.69**
Para-Pseudocalanus spp.	N-E Atlantic	**-0.14**	**0.00**	**0.61**
Pseudocalanus spp.	N-E Atlantic	**-0.10**	**0.01**	**0.52**
Temora longicornis	N-E Atlantic	0.00	0.96	0.00
Acartia spp.	North Sea	-0.09	0.10	0.24
Calanus finmarchicus	North Sea	**-0.20**	**0.00**	**0.74**
Calanus helgolandicus	North Sea	**0.27**	**0.00**	**0.72**
Centropages typicus	North Sea	**0.21**	**0.01**	**0.51**
Oithona spp.	North Sea	**-0.19**	**0.01**	**0.54**
Para-Pseudocalanus spp.	North Sea	**-0.15**	**0.01**	**0.53**
Pseudocalanus spp.	North Sea	**-0.11**	**0.02**	**0.42**
Temora longicornis	North Sea	0.02	0.21	0.15

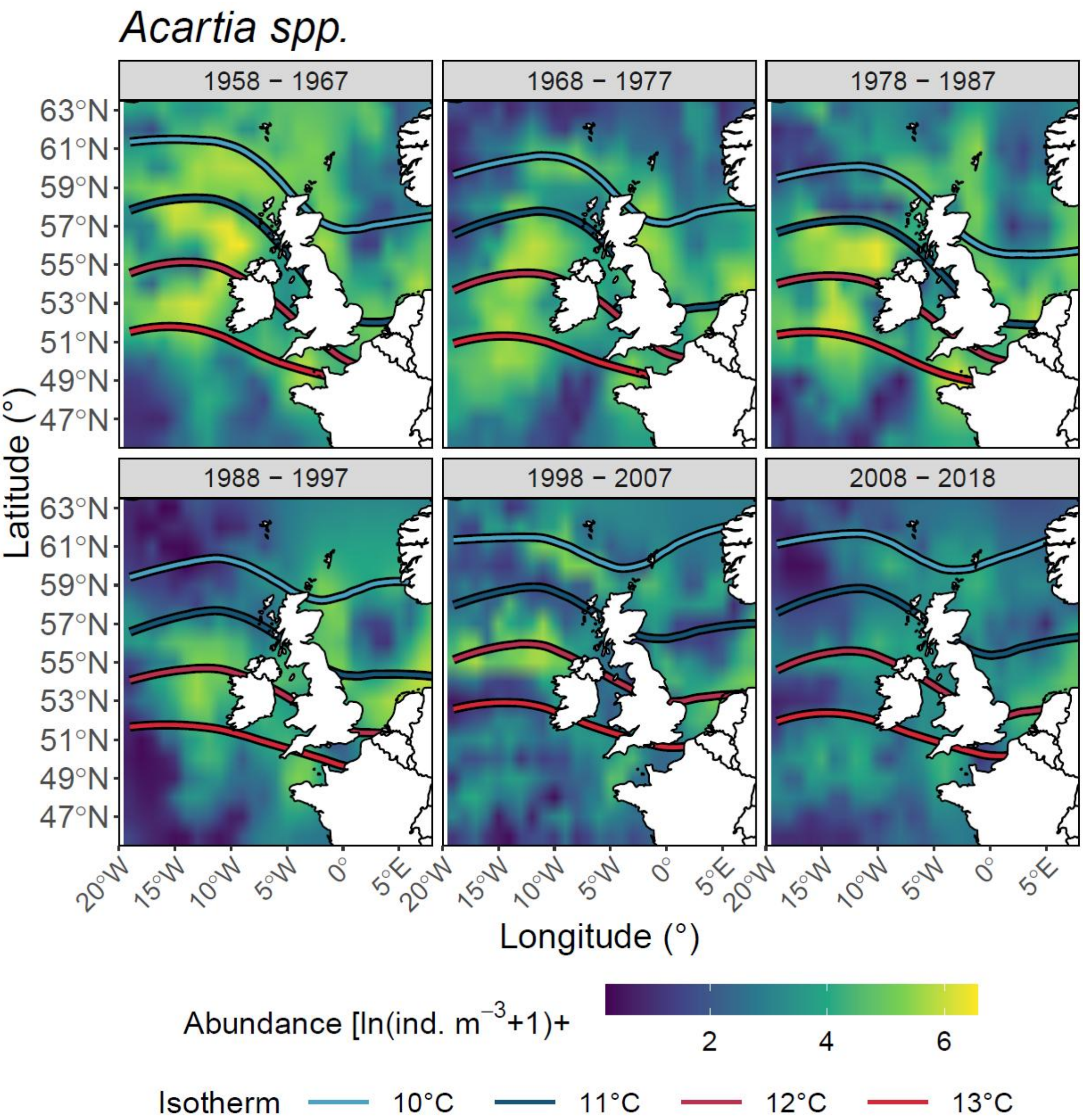

Figure S.1: Mean copepod abundance [i.e. ln(ind. m^{-3} + 1)] of combined pairs of five-year periods. Lines indicate the four different isotherms, colour indexed according to the legend at the bottom.

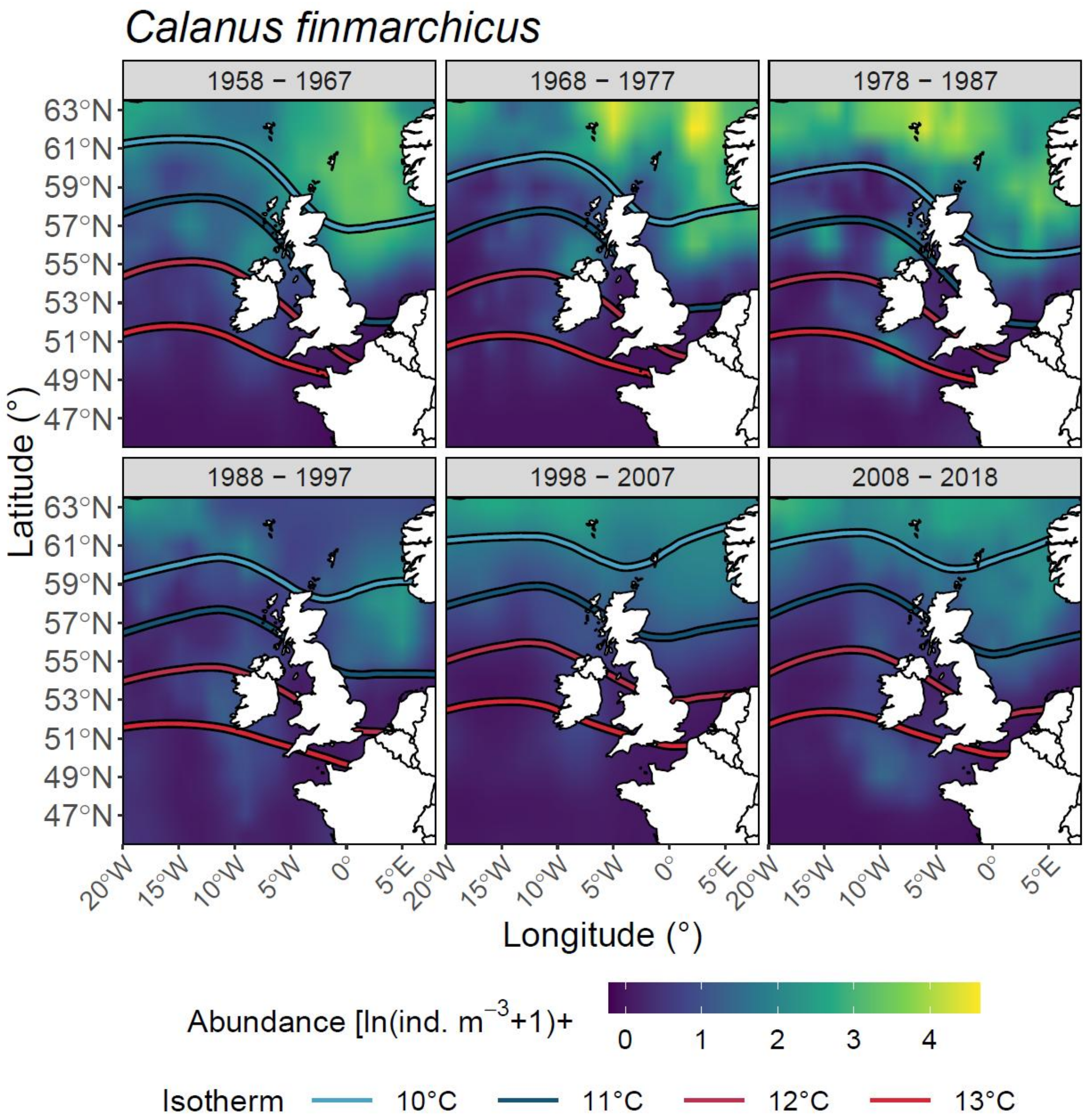

Figure S.2: Mean copepod abundance [i.e. ln(ind. m^{-3} + 1)] of combined pairs of five-year periods. Lines indicate the four different isotherms, colour indexed according to the legend at the bottom.

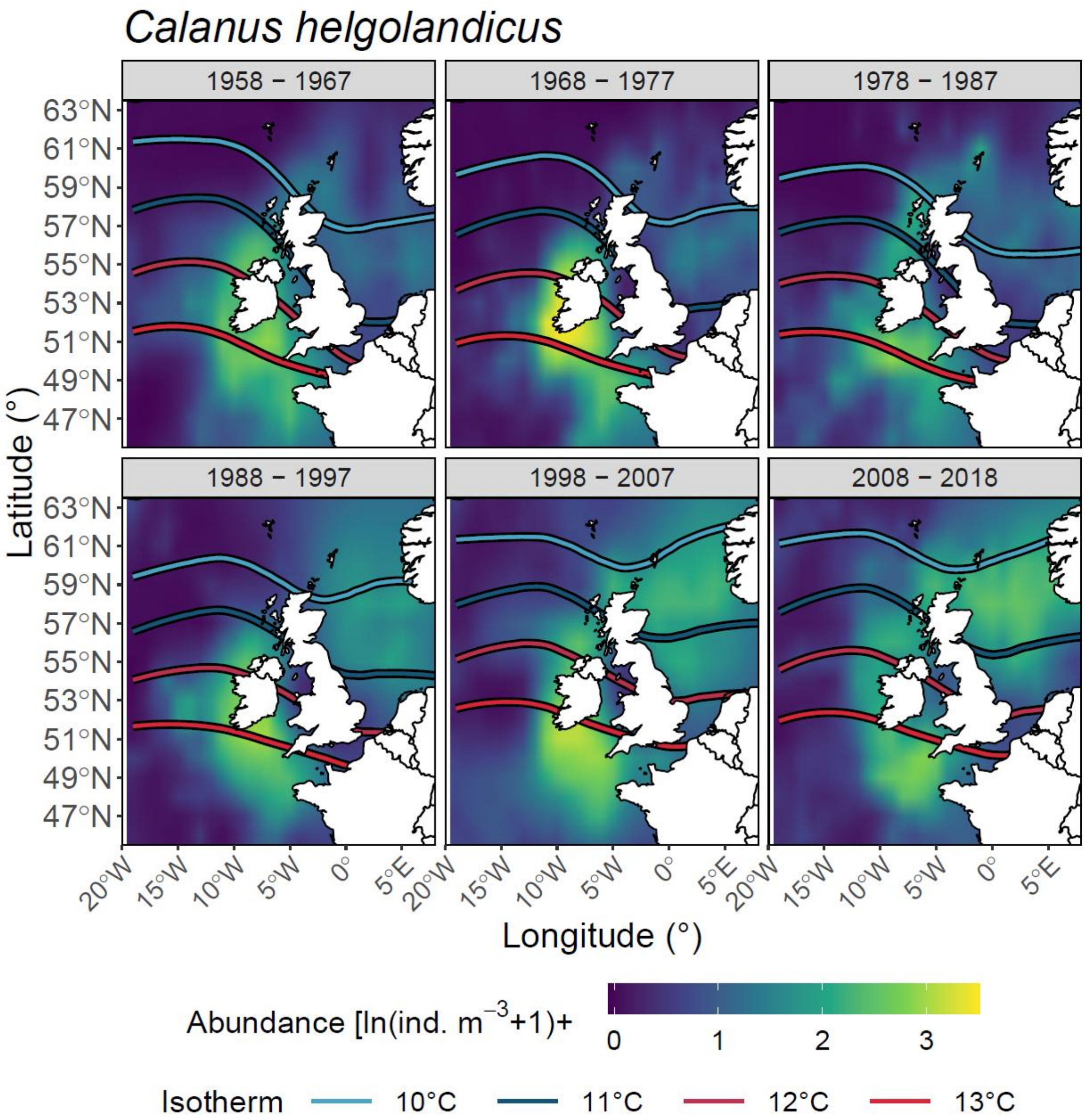

Figure S.3: Mean copepod abundance [i.e. ln(ind. m^{-3} + 1)] of combined pairs of five-year periods. Lines indicate the four different isotherms, colour indexed according to the legend at the bottom.

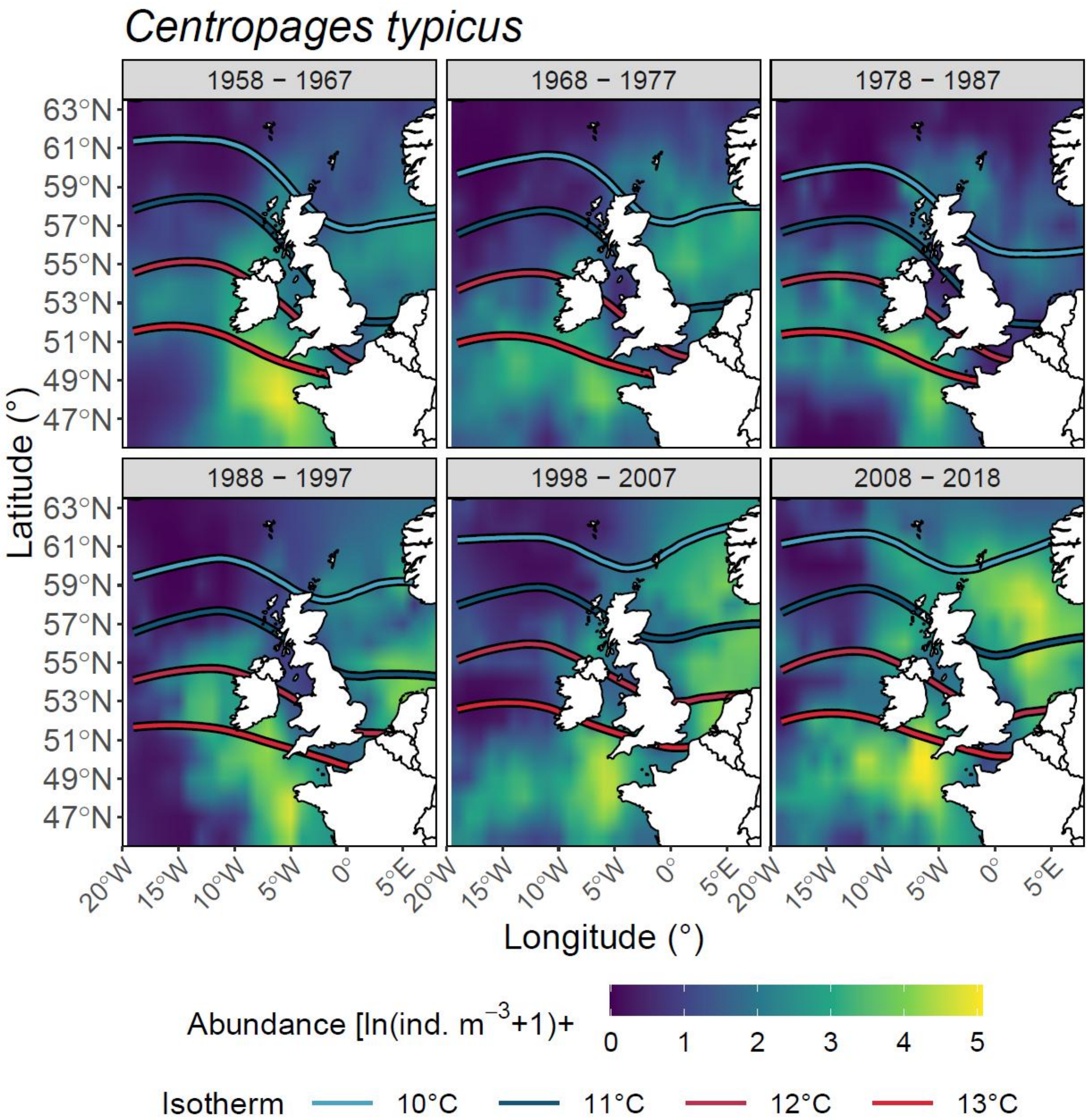

Figure S.4: Mean copepod abundance [i.e. ln(ind. m^{-3} + 1)] of combined pairs of five-year periods. Lines indicate the four different isotherms, colour indexed according to the legend at the bottom.

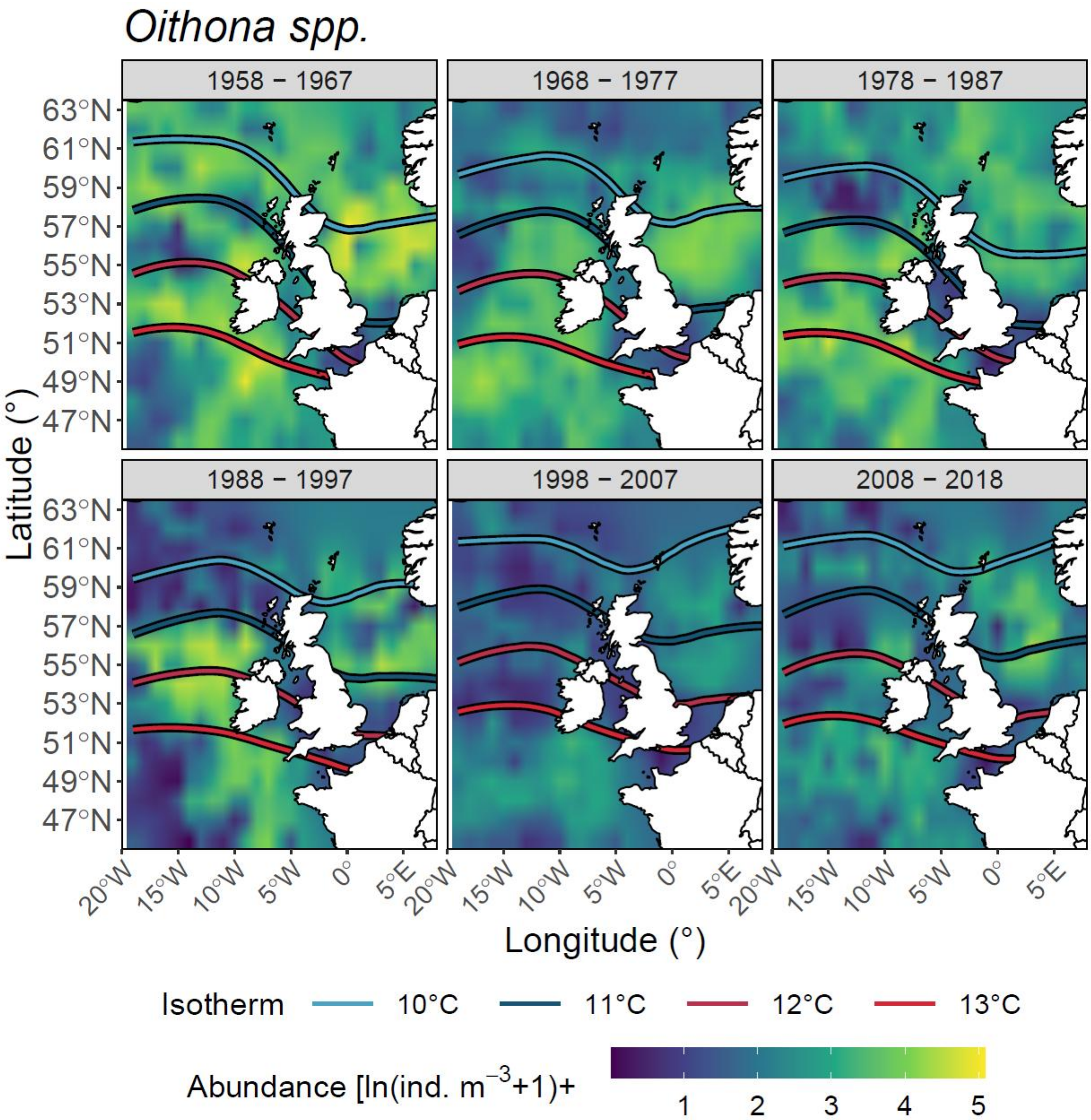

Figure S.5: Mean copepod abundance [i.e. ln(ind. m^{-3} + 1)] of combined pairs of five-year periods. Lines indicate the four different isotherms, colour indexed according to the legend at the bottom.

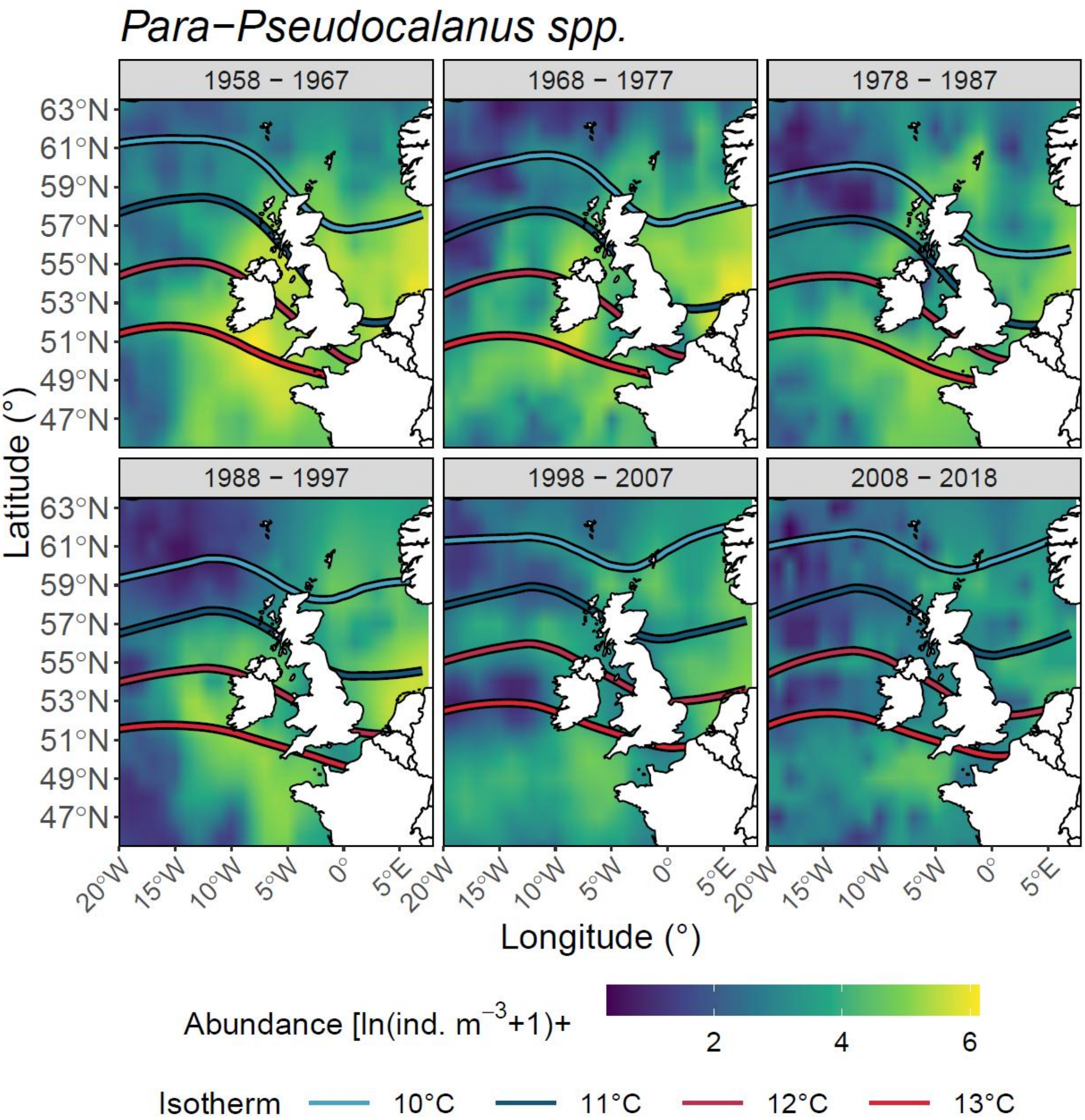

Figure S.6: Mean copepod abundance [i.e. ln(ind. m^{-3} + 1)] of combined pairs of five-year periods. Lines indicate the four different isotherms, colour indexed according to the legend at the bottom.

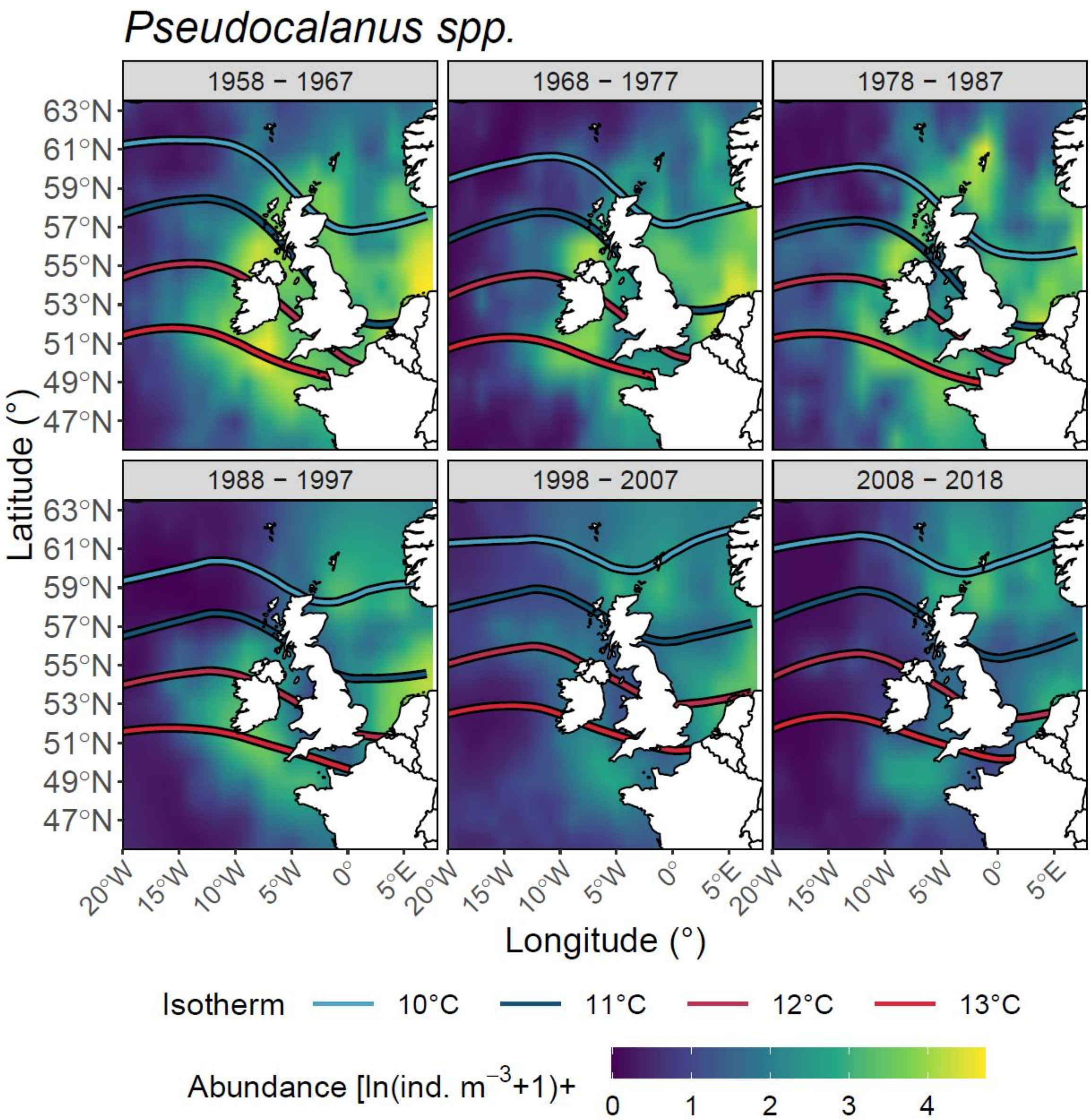

Figure S.7: Mean copepod abundance [i.e. ln(ind. m^{-3} + 1)] of combined pairs of five-year periods. Lines indicate the four different isotherms, colour indexed according to the legend at the bottom.

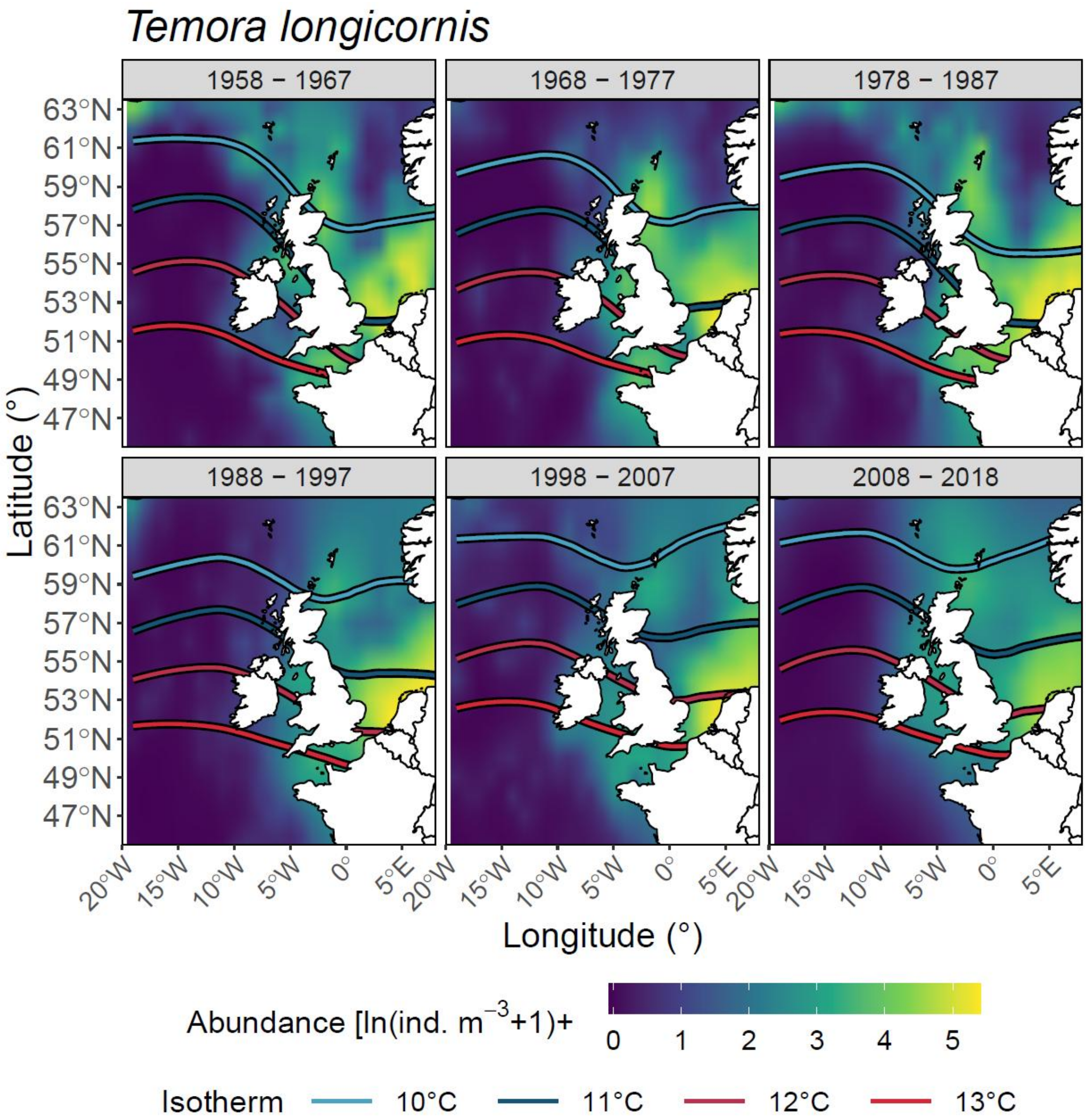

Figure S.8: Mean copepod abundance [i.e. ln(ind. m^{-3} + 1)] of combined pairs of five-year periods. Lines indicate the four different isotherms, colour indexed according to the legend at the bottom.

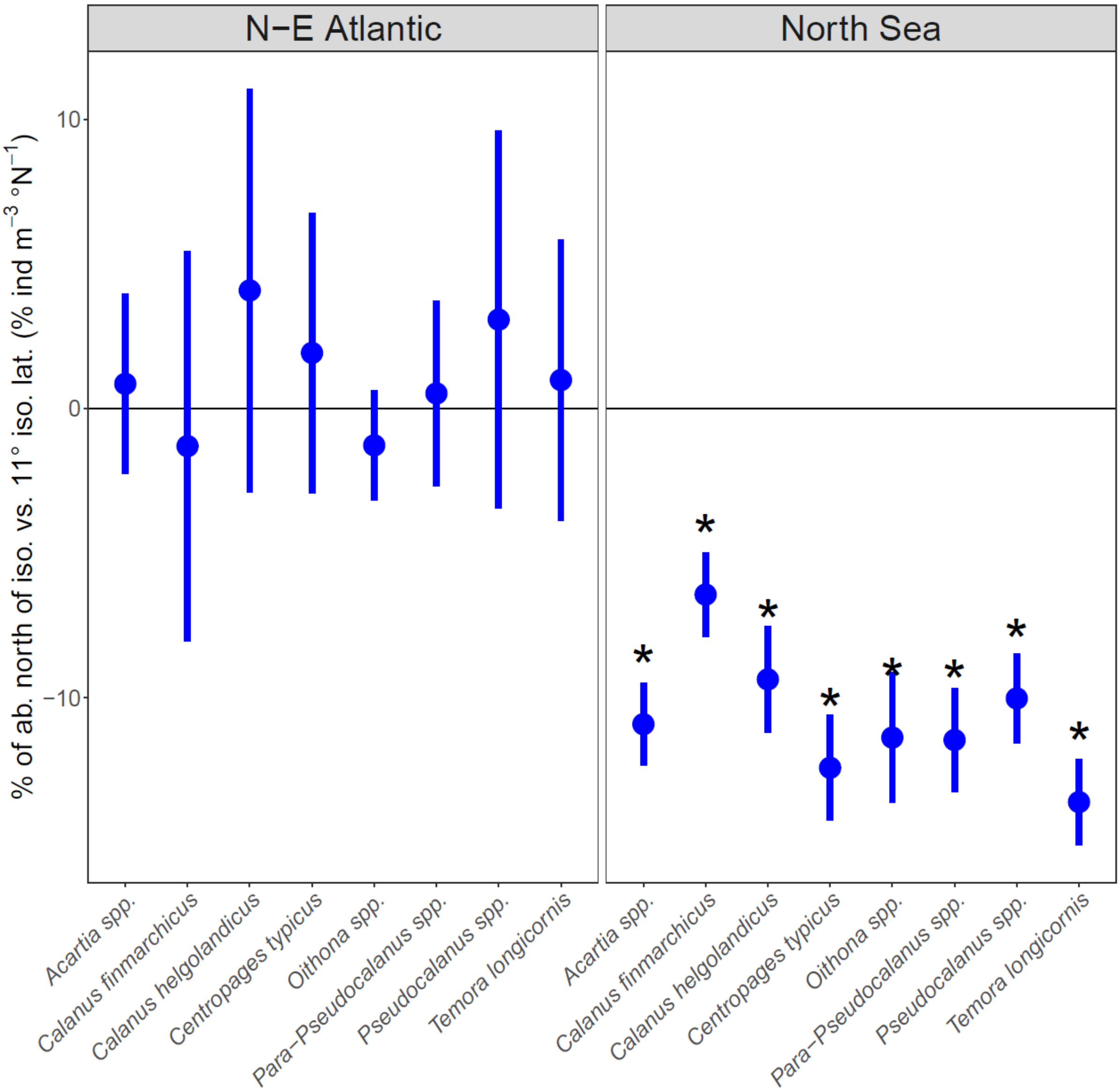

Figure S.9: Change of copepod abundance percentage north of the isotherm with warming. Taxon-specific linear regression slopes of percentage abundance north of the 11°C isotherm vs. mean 11°C isotherm latitude (% ind. m^{-3} °N^{-1}) across twelve 5-year periods (n = 12). Vertical error bars indicate the 95% confidence interval, an asterisk next to the symbol denotes a significant slope (* = $p \leq 0.05$).

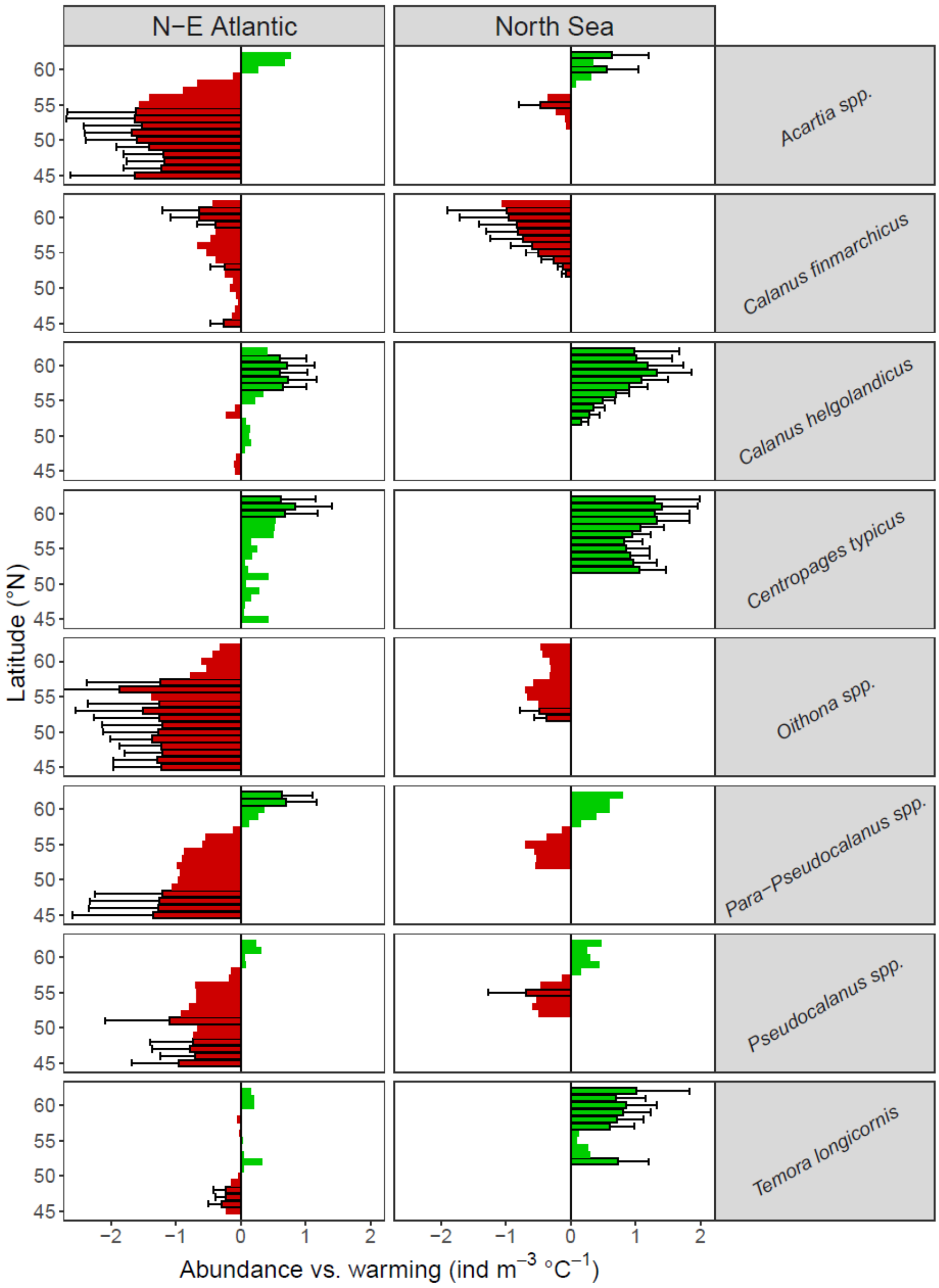

Figure S.10: Latitudinal variation in sensitivity to warming of copepod abundance between 5-year means across the period 1958 to 2018 : Bars indicate the slope values (x-axis) from the linear regressions of mean copepod abundance (log values) vs. 11°C isotherm latitude for each latitudinal degree (y-axis) of each 5-year period. Green bars on the right of the vertical black line indicate an increase, while red bars on the left indicate a decrease of copepod abundance with warming within that latitude. Horizontal error bars indicate the 95% confidence interval and are reported only for significant slopes. Grey areas represent missing values due to land presence at the given latitude.

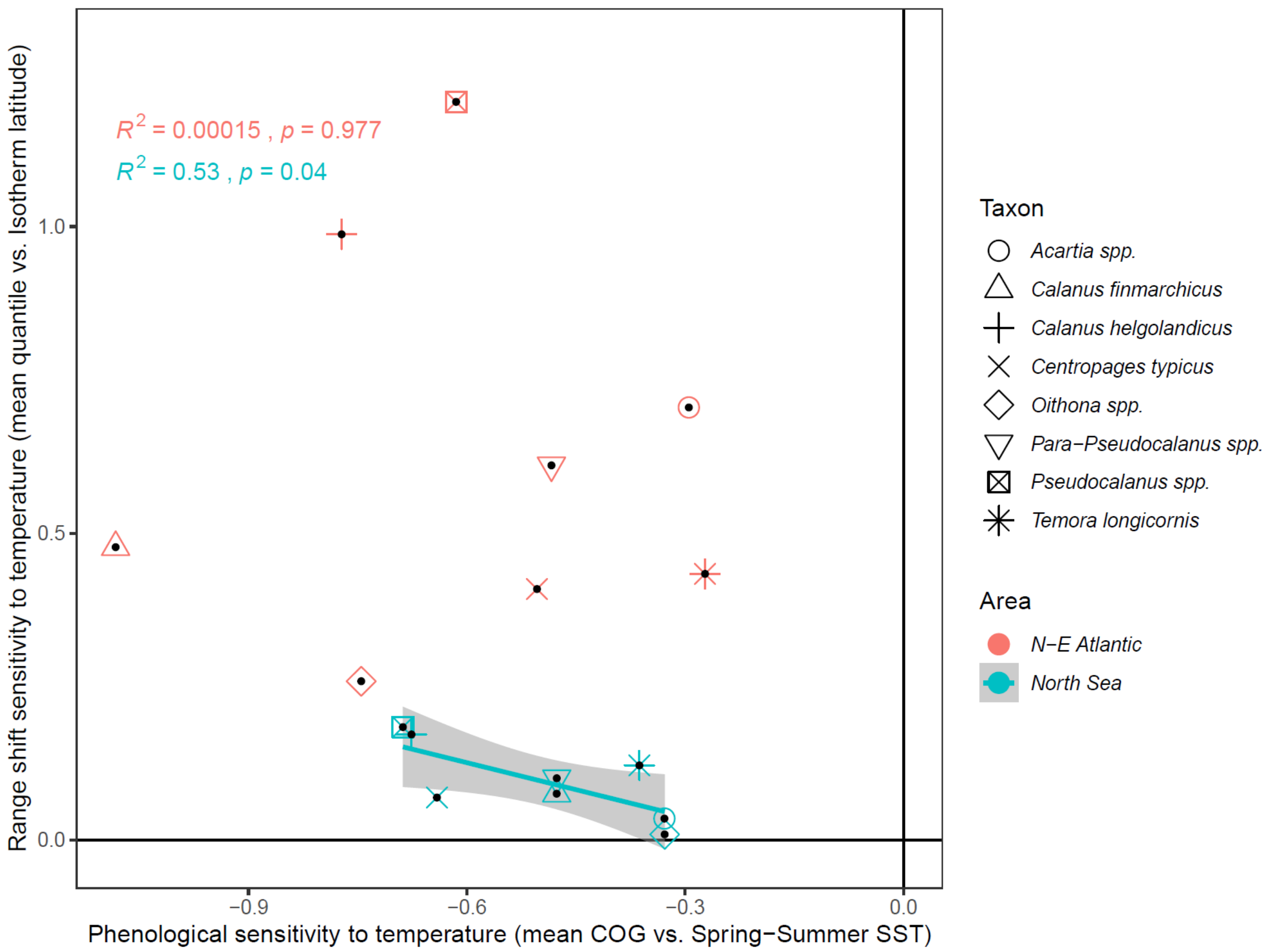

Figure S.11: **Relationship between range shift sensitivity to warming and phenological sensitivity to warming**. The y-axis indicates the thermal sensitivity of range shift: movement of quantiles (average across the 25th, the 50th, and the 75th) of latitude for copepod abundance with isotherm latitude change ($N°\ N°^{-1}$), meaning that values above the 0 indicate a northward movement with warming. The x-axis indicates the thermal sensitivity of phenology shift: change of COG month with Spring-Summer sea surface temperature (Julian month $°C^{-1}$), meaning that values < 0 indicate progressively earlier phenology with warming. Regression fit line is fitted only for the significant relationship in blue. On top-left are reported the R^2 and p values for both regressions (for both marine areas). The regression in blue means that there is a correlation between range shift thermal sensitivity and phenological thermal sensitivity, across species, in the North Sea.

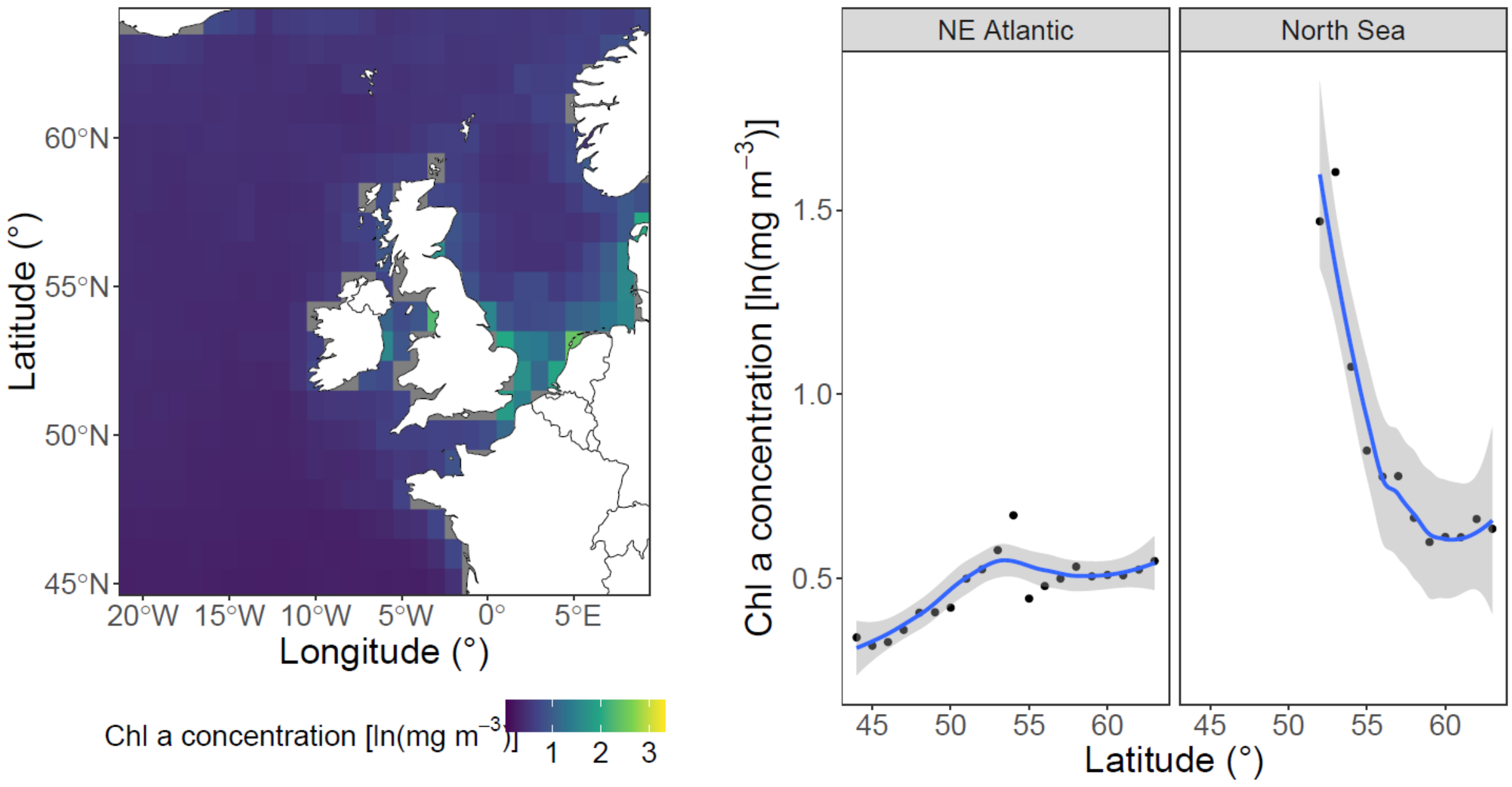

Figure S.12: Chlorophyll *a* concentration. **a)** Satellite-based (OC-CCIv6 data) for surface Chl *a* concentration across the study area; **b)** points indicate the Chl *a* concentration averaged for each 1° of latitude and for each marine area, blue line is the smoothed average (span level = 0.75). In both a) and b) Chl *a* concentration is measured as the mean value between April and September inclusive.

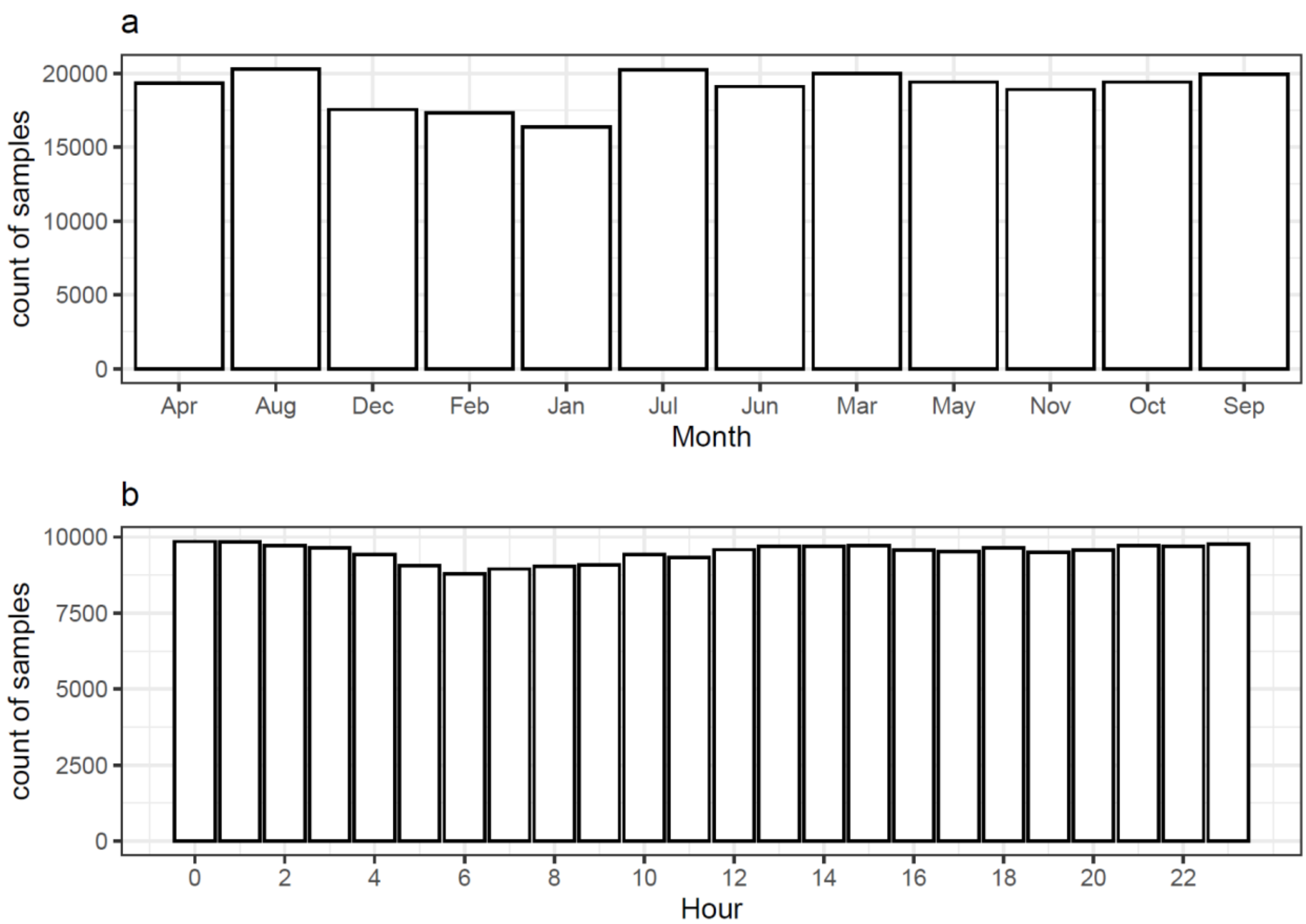

Figure S.23: The distribution of number of samples across months of the year (**a**) and across hours of the day (**b**)

643

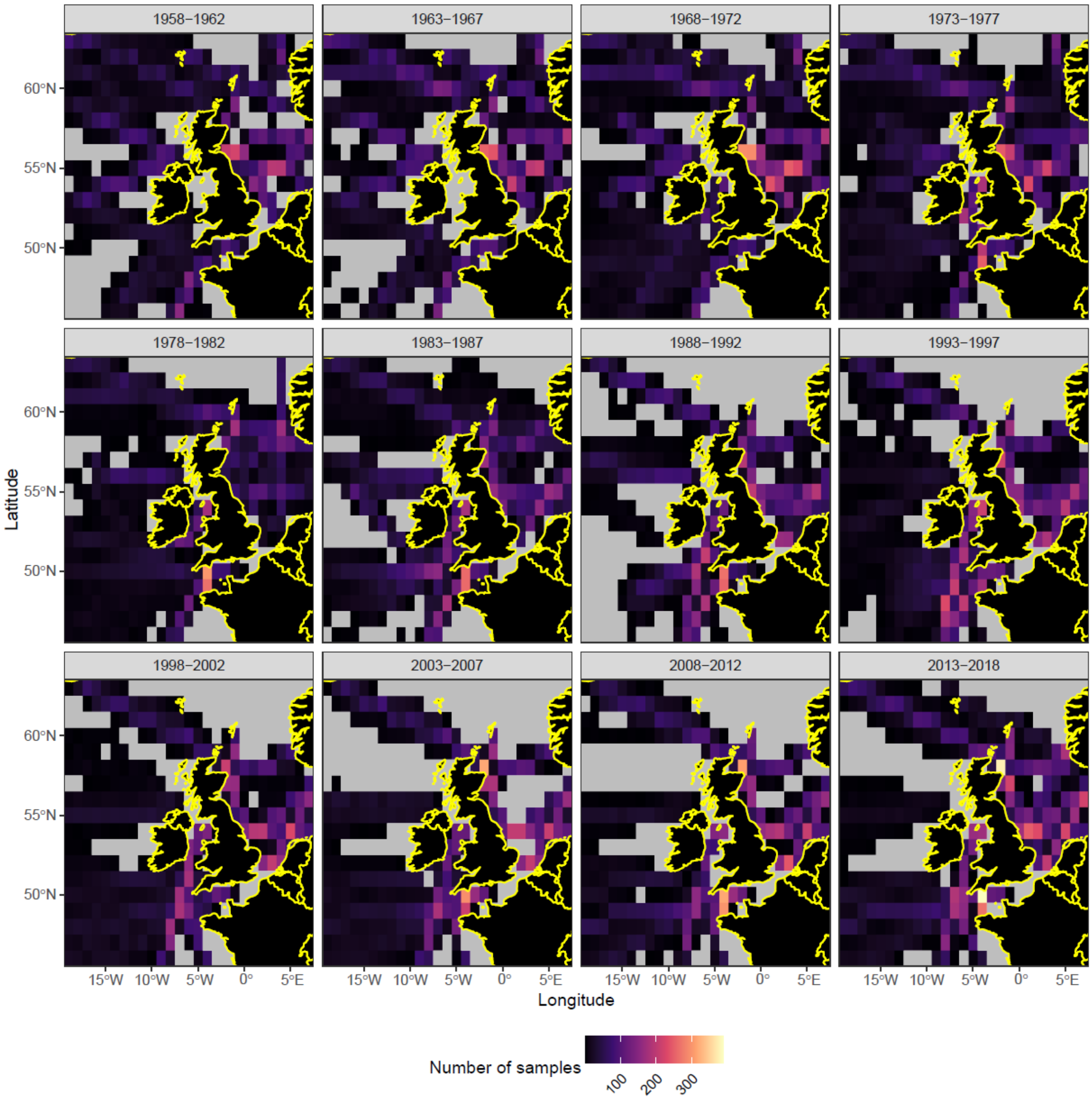

644 **Figure S.14**: Number of samples at each 5-year period. Grey cells indicate no samples.

645

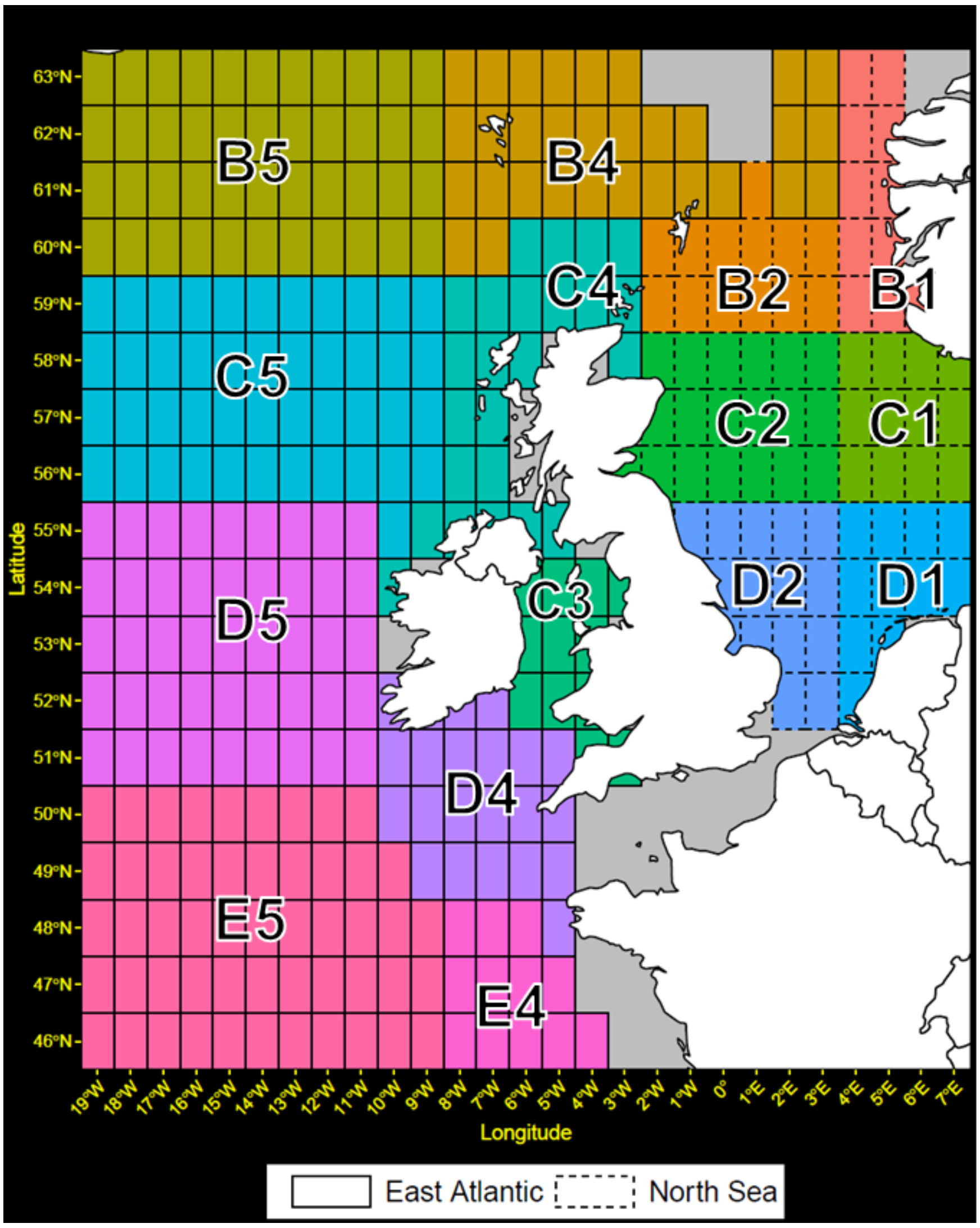

Figure S.15: Continuous plankton recorder standard areas. Straight and dashed lines indicate how we divided the North East Atlantic and the North Sea.
